# The Impact of Stakeholder Preferences on Service User Adherence to Treatments for Schizophrenia and Metabolic Comorbidities

**DOI:** 10.1371/journal.pone.0166171

**Published:** 2016-11-16

**Authors:** Daniel Poremski, Vathsala D/O Sagayadevan, Peizhi Wang, Alvin Lum, Mythily Subramaniam, Chong Siow Ann

**Affiliations:** Research Division, Institute of Mental Health, Singapore, Singapore; University of Texas Health Science Center at San Antonio Cancer Therapy and Research Center at Houston, UNITED STATES

## Abstract

**Objective:**

To determine how stakeholder opinions of treatments influence service user decisions to adhere to courses of actions necessary to treat metabolic conditions.

**Methods:**

Qualitative open-ended interviews were conducted with 20 service providers, 25 service users, and 9 caregivers. Grounded theory was used to generate an understanding that linked preferences of care with adherence to follow-up treatments.

**Results:**

Participants spoke about several considerations when discussing adherence: Resource limitations were the predominant consideration. Social considerations such as stigma and support surfaced in caregiver and service-user interviews. The influence of symptoms, especially their absence could reduce adherence, and organizational considerations related to the opinions they had about the qualifications of professionals.

**Discussion:**

A rational patient model partially organizes our findings, but emotional components related to stigma and the opinion of service providers do not fit well into such a model. If service providers do not consider components of the decision making process which fall outside of the rational patient model, they may incorrectly be leveraging suboptimal values to bring about adherence to treatment plans. Being sensitive to the values of service users and their caregivers may allow service providers to better act on points that may bring about change in non-compliant service users with schizophrenia and metabolic comorbidities.

## Introduction

Monitoring for the development of metabolic comorbidities in people receiving psychopharmacological treatments may be as basic as taking blood pressure, or completing blood tests to check for elevated glucose levels and elevated lipid levels. These routine investigations may raise red flags if abnormalities are found, red flags that may alter the care-plan to a medication with a better side-effect profile [1]. Since these tests may be time sensitive, it is important that they be completed regularly [2] especially in the early stages of treatment [3]. However, the rate at which people with diagnoses of schizophrenia and related psychoses receive these routine assessments is low [1, 4–8], pointing to an important area of potential improvement. Because monitoring and treatment rates have been low, this segment of the population is beginning to show increased incidence of untreated comorbidities associated with long-term exposure to specific medications [1, 8–11]. Additionally, and possibly as a result, people with schizophrenia have shorter life expectancies than their healthy peers [8, 12]. In the UK, people with schizophrenia lived 12.7 years fewer (14.6 for men, 9.8 for women) [13] than the average population. A greater awareness to this situation has led to initiatives to increase adherence with medications needed to treat comorbidities [5], but, as Gorczynski and colleagues note, more research is needed to expand our understanding of the way in which people deal with their comorbidities and associated treatments.

Problems of adherence and treatment retention are ubiquitous in mental health treatments [14]. In the case of treatments for metabolic comorbidities several factors may account for the low rate of monitoring and the subsequent large treatment gap. First, the nature of the illness may prevent people from seeking help for symptoms they may not easily identify as abnormal. Awareness of one’s personal health may be altered or diminished as a result of mental illness [15]. Second, the impetus to seek care may be limited by patient preference. Some service users have difficulty establishing rapport or working alliance [16, 17], and consequently feel most comfortable dealing with one service provider, shying away or dropping out from settings like group practices or polyclinics (Polyclinics provide general and specialist outpatient services and treatments either by appointment or same-day appointment) where they may not be seen by the same practitioner at each visit. Furthermore, people with schizophrenia may, compared to people with other illnesses, have a higher tolerance for symptoms and lower preference for treatments [18] which affect their motivation to follow-up with certain treatments [19]. Third, practitioners may not feel comfortable following up with patients treated by tertiary care and who may be difficult to engage because of their mental illness, despite evidence that General Practitioners may be effective at fulfilling this role with minimal support from psychiatrists [20]. Because the source of this treatment gap is not fully known, additional research is needed to determine which barriers to care may be preventing people with schizophrenia from obtaining necessary medical examinations, and what may be preventing physicians from actively providing the necessary screening even when prompted by automated reminders on patient electronic records.

### Purpose

We sought to understand what stakeholders considered when discussing service user adherence to courses of actions necessary to treat metabolic conditions. Our investigation will shed light on which preferences influence people’s decision to follow-up with treatment for metabolic comorbidities (hypertension, diabetes mellitus, and hyperlipidemia), and their understanding of psychiatric treatment and its impacts on physical health.

## Methods

Our qualitative research project and the content of this manuscript followed the Consolidated Criteria for Reporting Qualitative Research (COREQ): a 32-item checklist for interviews and focus groups. This adherence is intended to increase the transparency of the qualitative process, and increase confidence in the methodological process, its rigor, and its findings [21].

### Setting

Singapore’s health care system is divided between hospitals (specialty and institutional care), publicly subsidized polyclinics (multidisciplinary treatment centers) and private practices. Polyclinics represent a readily accessible source of care integrated into the community as part of civil planning. The mental health institute where the research was conducted represents the only national level source of specialized tertiary mental health care in Singapore. This institute has a capacity of approximately 2000 inpatient beds and runs outpatient and emergency services for the entire country of 5.5 million residents. In cases with comorbidities, it is habitual for service seekers to visit the institute for psychiatric care and visit a polyclinic for physical health care.

The cost of care is not automatically subsidized and fees are borne by the service users directly. In cases of limited financial means (household income less than $1,800 SGD/ month/ person) government subsidies are available to cover a certain portion of the fees [22]. In cases of severe financial need (people already receiving subsidies who continue to be unable to pay for essential services) further means-tested financial assistance is available through Medifund [23]. Subsidies are only available at public health care facilities, and Medifund eligibility is assessed on a case-by-case basis at the health center where care is delivered, with the implication that medifund eligibility in one location does not guarantee eligibility elsewhere.

### Participants

We spoke with three groups of participants, in sequence, for the purpose of providing several perspectives on the development of treatment preferences. First, we interviewed service providers, then service users, and completed the study by interviewing caregivers.

### Recruitment

The recruitment target was 20–30 service provider and user participants, and 10–15 caregiver participants. These targets were based on previous recommendations for grounded theory studies [24]. Recruitment ceased once the content of the interviews began to repeat itself, and no new elements related to the treatment of comorbidities begin to emerge. This is the point of theoretical saturation [25].

However, the initial target goal for caregiver participants assumed that a caregiver would accompany a greater number of service users. Many service users were independent and therefore the target for caregiver recruitment was not reached. This is not a limitation of the study, but simply a sign that our initial sampling expectations were based on the erroneous assumption that this segment of the service user population would be heavily dependent on caregivers and family. This was not the case and caregivers played less of a role in determining service user adherence than expected.

Outpatient clinic physicians who dealt with service seekers with a diagnosis of schizophrenia and any one of the metabolic comorbidities helped recruit service users by referring them to the study team. Theoretical sampling was used to recruit equal number of service users who had been successfully referred to outpatient care for their metabolic comorbidity and service users who had preferred to be treated for their metabolic comorbidity by their psychiatrist.

We used a snowball method to recruit service providers, beginning with senior management. The senior management referred us to other members of the staff who subsequently referred others. This method was chosen to help develop a picture of the organization’s policy that followed the hierarchy of roles. It is likely to provide a more systematic exploration of the content [26] and provides a better way of gaining entry into the sample pool [27].

#### Eligibility

Service users were eligible if they spoke and read either Chinese or English, had a diagnosis of schizophrenia or psychotic disorder, a diagnosis of one or multiple metabolic conditions, and were above the age of 21. Caregivers looking after service users who fit the criteria above, either on a part-time or full-time basis, were eligible for the interview if they could speak and read either Chinese or English. Service providers were eligible if they routinely provided services to people with schizophrenia or psychotic disorders.

### Procedure

For this qualitative study, two co-authors, (any combination of DP VS and PW) jointly conducted the service user and caregiver interviews. DP conducted the majority of the service provider interviews alone. The pair debriefed after each interview and noted unique and repeating themes of the interviews. We used these notes to construct the preliminary coding list used in the data analysis. The pair asked open-ended questions arranged in a structured manner to guide the interview along the narrative of the individual’s service use history, querying their choices and the underlying reasons behind these choices as the interview progressed. Interviews were conducted in English or Chinese. Interviews lasted an average of 43 minutes (SD 11). All participants gave written informed consent in either English or Chinese depending on their language of preference. The Institute of Mental Health Clinical Review Committee and the National Health Group Domain Specific Review Board Domain F2 Population Health approved the study (NHG DSRB Ref: 2015/00809).

### Analysis

We chose the grounded theory methodology to approach the data collection and analysis [28]. DP VS and PW transcribed verbatim and coded the audio-recorded interviews in Nvivo 10 [29] separately in their original language. Three of the 25 service user participants declined audio recording as did one of the nine caregivers. We took notes during these four interviews and compiled them immediately after the interview. The team generated codes and discussed their relevance at periodic meetings over the course of the study. A constant comparative method was used to adjust interview questions based on emerging themes [25]. Stakeholder groups were coded in sequence to avoid confusing the content of each code. Service provider participants were coded first. We used the same coding list for all three types of participants. When a new code emerged, we discussed its merit and recoded previous interviews with the specific goal of searching for content relevant to the new code. We coded the interviews in their original language. We did not include content from the Chinese interviews in the results below. The quotes below have been edited for language clarity as most of the service user and caregiver participants were not native English speakers (For example: “maybe within 3 months I get relapse” became “Maybe within three months I’ll have a relapse”). All anonymized codes used to construct the themes reported below are available in the Supporting Information Files in their original form (S1 File).

#### Reflexivity

The question of whether psychopharmacological agents directly lead to various comorbidities is still contentious, especially given the range of side effects. Some conditions such as weight gain may be physiologically linked to antipsychotic compounds via their dopaminergic effect on the tuberoinfundibular pathway [30], but other comorbidities like diabetes have other causative factors. Given that the link remains contentious, it is relevant to note that psychiatrists hold differing opinions, even within research teams. The interviewer (DP) is of the opinion that the links between the comorbidities and exposure to antipsychotic agents is sufficient to be considered causal. This opinion likely surfaced in the way questions were asked and probed, especially when discussing who should be responsible for monitoring and treating such conditions. This was likely more pronounced in the interviews with service providers. However, the fact that psychiatrists may not have clearly explained the link to service users and caregivers was an important consideration when speaking with service user participants.

A second point of consideration is that the ethnicity of the lead interviewer did not resemble that of the participants. This may have had some impact on the responses of certain participants, especially those whose first language was not English. Three interviews were conducted entirely in Chinese, with several other service user participants using Singlish colloquialisms during the interviews. Singlish is a creole language which mixes Chinese Malay loanwords with English. These colloquialisms often emphasize emotion and punctuation. For example “lah” is a Chinese particle of speech colloquially used in Singapore to give emphasis. It is the only Singlish colloquialism included in the quotes presented below. As a result, the lead interviewer, who has basic understanding of Singlish but not Chinese, may not have probed some participants at opportune moments. The team, however, does not believe that this negatively influenced the content of the interviews or the rapport created between the interviewers and the participants.

## Results

We interviewed 20 service providers, 25 service users, and 9 caregivers. Recruiting caregivers was particularly challenging. Several caregivers declined participation because of time constraints. Such participants were offered the opportunity to return on a day that was more convenient, but these offers were not accepted. Others declined because they did not “want to get involved with institutional things” like research.

Participants’ believed that adherence depended on several considerations, some of which usually hindered and others that facilitated adherence.

### Service user explanation for metabolic conditions

Service users, by virtue of the screening of participants, were all aware of their diagnosis of schizophrenia and the metabolic comorbidity, but the extent to which they understood the link between the two varied. Given that, for some, the choice to begin anti-psychotic medication was made during psychiatric emergencies, they did not always receive a full explanation of the potential side effects. Often the psychiatrist responsible for the follow-up care after their discharge was responsible for the task. According to the accounts of the service users, psychiatrists used simple language to make the link between anti-psychotic medications and weight gain, but did not always explain the metabolic comorbidities that may accompany weight gain. As a result, the service users’ explanation of why they developed the conditions remained relatively simple, often only relating to the foods they consumed “I can’t eat too much sugar anymore” Service user 133.

### Resource constraints

Cost and time constraints are expected, and surfaced in every interview as a main element. Service provider participants believed that the frequency with which service users and caregivers had to attend appointments, and the resulting time commitments, played an important part in determining if service users would engage with treatment. This was in concordance with caregiver and service user accounts. However, the account of service users was not unanimous. Some service users worried about relapses if the frequency of their medical appointments was reduced to ease the time burden:

The doctor asked whether I would like to have three months’ worth of medicine, instead of having to come every month. I say “I cannot lah”. Like that? I tend to… after [such a period of time], I might forget everything. That’s why I don’t dare to try it. […] But I said “I cannot accept it. I’m scared the feelings will come back”, maybe within 3 months I will have a relapse. I cannot try. Service user 138

For these service seekers, attending the appointment with their psychiatrist was as important to the efficacy of their treatment as taking the prescribed medication. This is especially surprising given that the appointments were brief and did not provide therapy, such as CBT.

### Social considerations

#### Social supports

A minority of our service user participants mentioned relying on social support groups and informal peers to help them understand their illness and deal with the burden of their complex treatment plans. This social support played an important role in increasing their adherence:

I changed because of my social networks. So I’ve been taking his [peer specialist’s] advice. I meet him, just for one hour. Because he’s been suffering, I think more than 40-over years. 50 -over years […] So, it is kind of like mentoring. […] You know, have a coffee for a one hour session. Just to ask him “what are your fears?, what are your paranoia?, what are your conditions? And what…what can you”…what can I gain from him. The knowledge, basically. Service user 140

These participants tended to be younger and actively sought these types of connections. Older participant spoke less of being engaged with other service users unless they lived in supported housing where the presence of other service users was ubiquitous. Families offered varying degrees of support and could be entirely uninvolved: “I didn’t tell my family because I don’t think they…I don’t think they can actually help me lah. Because…part of the stress comes from the family” (Service user 134). For those with higher needs, family played an indispensable role, however. It is caregivers from this latter group which made up our caregiver sample.

#### Stigma

Stigma features heavily in the way caregiver and service user participants spoke about their preferences for treatments. For treatments which require regular visits to an institute, coming frequently was seen as potentially exposing the family to societal stigma. This was particularly challenging for people who came more frequently for auxiliary services such as occupational therapy and vocational training. Stigma was usually associated with the way the caregivers believed they would be perceived:

I think [my mother] just worries about, like, people judging us. Because some people, they don’t like people with mental illness, or they think bad things about them and they say bad things, and I think she just wants to protect me from that. Service user 149

When families and caregivers had such feelings, it elicited conflicting emotions in the service users:

I feel happy that she wants to protect me, but also I feel like… there will come a time when I will, when someone will be prejudice against me because of my mental illness and I will have to deal with it, and what is important is not what they think, but my mental health. That is the important thing. Service user 149

Beliefs about the symbolism of attending mental health services heavily influenced whether caregivers would be willing to accompany their kin to appointments. This was especially relevant during the early stages of the illness: “They don’t like me to go IMH because IMH is very old, and has many very complicated people. So they decided I should see doctors through Mount Elizabeth’s private psychiatrist” (Service user 137). But as noted above, the cost barriers of private care meant that sustaining preferred source of action could be difficult and families had to think differently about what it meant to attend services.

Service provider participants believed that their service users may experience stigma from service providers who were unfamiliar with mental illness:

Yes, I think there is this bias amongst, out there in the medical community, about patients with mental illness. They sometimes think they invent their symptoms, their physical symptoms especially. And just because they have a mental illness, they think their physical illness is not as important. So I think, I am concerned that some patients are seen in the community, and their physical illnesses are not take seriously enough. Service provider 096

They understood that these experiences may alienate service users from certain professionals, reducing their desire to engage with the necessary care and ultimately reducing their willingness to follow-up with treatment for their metabolic conditions.

### Illness considerations

#### Symptom interference, or lack of interference

Service provider participants were cognizant of the fact that metabolic conditions tended to be forgotten, and therefore neglected, because the physical manifestations of the illness could easily go unnoticed. As a result, service providers worried that service users could easily forgo the follow-ups and referrals necessary for the treatment and monitoring of the metabolic conditions. Service user accounts support their concerns. Several service user participants explaining that they had not followed-up with the treatments for their metabolic comorbidities because they did not feel “sick”. To some of our participants, the reasons for adhering to treatments were simply numbers on a page in the doctor’s office. Seeing lab results reporting high cholesterol did not have an impact on their decision to adhere to treatments or attend follow-up appointments. This led service providers to prefer to treat the metabolic conditions to the extent of their abilities.

Even if you refer a patient to polyclinic or hospital, they often don’t go. So they remain untreated, and all the while we are thinking that they have gotten treatment. So, engaging the patients and ensuring that they follow-up elsewhere, uhm, is a challenge. So we thought “why not do it in-house?” at least to the extent possible. Service provider 110

Some service user participants described that they had in the past chosen to stop their antipsychotic medication due to the lack of symptoms and because they felt better. This could lead to the manifestation of new symptoms, which interfered with the treatment of their metabolic comorbidities. In these cases, such relapses could precipitate the development of insight.

#### Insight

For our service user participants, previous experiences with non-adherence prompted by periods of symptom absence led to relapses. These relapses were especially troubling for those who had experienced longer periods of stability or had only recently been diagnosed. After having been hospitalized and prescribed antipsychotics anew, their symptoms subsided. This experiential knowledge also developed in the families and led them to perceive their control over their symptoms and attribute their ability to control their symptoms to their adherence.

So far, when she is stable, right now, I would say that she is religiously taking her medicine. But I mentioned to you earlier that there will be a few occasions over one year, two years, when suddenly her emotions will become unstable. I would say prior to that, there may be certain triggers that tick her, or maybe she didn’t take her medicine, and then that causes her to become sick. But now, when she is stable, with me she has been taking [her medications], and she knows that if she doesn’t take them, she will start to become agitated, and all that. She realizes all this. Caregiver 163

It is these experiences they wish to share with their younger selves and others:

I would advise them to go for medical checkups. I mean, like for the psychiatric checkups. […] Because, I mean, what’s the point of having like… having attacks every other day when you can prevent them from occurring? Like, after one attack you come back for follow-up. Then, I’m sure that the attack would be fewer or even prevented. Service user 112

However, others believed that the fact they had multiple diagnoses eliminated their control, making adherence a necessity:

But then… but what am I supposed to do? It’s already been diagnosed that I have this…so many medical conditions…so, I really don’t have that much of a choice but to seek treatment. Otherwise, things go haywire. Service user 121

Concerning metabolic conditions specifically, the weight gain was tangible to service users, but hypertension and diabetes were invisible and symptoms were infrequently recognized. Because these symptoms did not have the same impact as psychotic symptoms (in terms of how they perceived the world), participants’ awareness of the reasons for adhering to treatment was based on rote rather than experiential knowledge.

I need to control my cholesterol level. Because there will be other complications. Like heart [problems]. You may get heart disease if you don’t control your cholesterol levels … because of that I… don’t… I’m alright with taking the medicine. Service user 134

### Organizational considerations

Some service users and caregiver participants considered the professional’s qualification when talking about whether or not they would want to follow-up with services. Some service user participants preferred care delivered by specialists because they worried about receiving treatments from doctors whose primary expertise were other than the treatment of their illness, for example receiving psychiatric services from a general practitioner.

Because I believe in specialization. And I know one doctor cannot specialize [in everything]. They can manage the medicines. […] But I don’t want one doctor for both cases, because I don’t think that’s possible. Service user 145

They did not follow-up with referrals to certain professionals, but rather waited or asked for referrals to specialists. When the service users connected with the type of professional they preferred, instructions could suffice to ensure adherence: “One of the doctors actually told me that I should go to the polyclinic to see the doctor for some of my conditions. So I went to the polyclinic” (Service user 118). While this is quite a simplistic view, the working alliance service users had in these instances played an important role to persuade service users to seek the necessary services.

Service provider participants believed they were sufficiently qualified to treat uncomplicated cases of metabolic syndromes, but usually preferred that their service users obtain care from a specialist, echoing the preferences of service seekers. As one service provider participant explained, this related to the focus of the professional:

We have two problems: one you dilute the psychiatrist’s focus if you then treat the whole thing. Of course it is great for holistic medicine, but nobody does everything well. […] And the other reason is that we are not, we are really not the best at diabetes, hypertension and hypercholesterolemia. These are not simple conditions, you have an entire specialties devoted to that sort of thing! Service provider 095

Their choice to treat uncomplicated cases was their way of decreasing the service gap and increasing adherence. Furthermore, by treating the metabolic comorbidities to the extent possible, they leveraged their working alliance to bolster the importance of treating metabolic comorbidities.

## Discussion

Our goal was to understand what stakeholders consider when discussing service user adherence to treatments, including treatments for metabolic comorbidities. Our participants mentioned several considerations that impeded their ability to adhere with treatments, and several considerations which facilitated their adherence.

Approaching the problem of adherence from the perspective of the rational patient allows us to organize some of our findings. Barriers related to attitudes of the impact of the illness, and facilitators related to the subjective norms of following physician instructions fall well into the framework of the Theory of Planned Behavior [31], which has shown relevance in several domains of adherence in chronic conditions [32]. Cost and time constraints are clearly important factors in actual behavioral control, and past experiences with non-adherence may lead to the individual’s perceived behavioral control [31]. The fact that some of our patients followed prescriptive instruction in a normative fashion fits with the model and is consistent with previous observations: In patients with diabetes, the compliance with medication prescribed has been higher than the adherence to exercise and diet regiments [33] as taking medication is more normative to the sick role. However, Corrigan and colleagues argue such rational patient models neglect important components in the decision making process, namely the emotional, implicit, and immediate nature of health behaviors [34]. Emotional considerations were important for our participants. For our caregiver participants, the emotions resulting from the stigma of bringing their kin to an institute impeded adherence with outpatient follow-ups. Service user worries of stigma impeded their involvement of family members, but when they had resolved fears of stigma, they were able to productively use peer supports.

While we expected more service users and caregivers to discuss their working alliance with service providers, their accounts relate to their opinion of professional qualifications. As noted throughout our article, cost had multiple impacts, and those that consistently sought services from the institute saw a rotating pool of service providers rather than one single provider (a more costly alternative). This limited their ability to form working alliances, and therefore they based their opinion on the qualifications of the physician rather than their past relationship. Receiving services from a rotating pool of physicians could be problematic as some service users have difficulty establishing rapport [16, 17], and consequently feel most comfortable dealing with one service provider, shying away or dropping out from settings like group practices or public clinics where they may not be seen consistently by the same practitioner.

The fact that some participants were reluctant to seek treatment for additional comorbidities can be partially explained by the findings of previous studies. People with schizophrenia highly value their independence, and valued being in a disease state higher than being in a side effect state [18]. This partially explains adherence variability as their preference for independence may lead them put more importance on their own feeling of wellness versus their lab-reported wellness. Their preference to avoid side effect states may lead them to decline follow-up treatment for their metabolic comorbidities thereby avoiding further medication and limiting their risk of additional side effects.

Collaborative care models using multiple approaches are an important direction for addressing issues faced by our participants [35]. By organizing the health professionals in such a way as to facilitate the transfer of expertise in a timely way, delays in screenings and medication adjustments resulting from time constraints or lack of expertise may be eliminated. Such models have been shown to reduce costs, increase professional and patient satisfaction [35]. These models are likely to be especially applicable in a setting such as Singapore where service user opinion of the professional qualifications may play a greater role in determining adherence to prescribed courses of treatment: if the team providing services is seen as more professionally divers and qualified, adhering to treatment plans may be more acceptable.

### Implications

The main implication is the way in which physicians negotiate treatment plans with their service users as rational patients. The result of negotiations may not be as anticipated if a physician falsely believes that the service user’s choice to comply with an action depends heavily a certain value, and then mistakenly leverages that value to bring about adherence with the proposed course of treatment. If the physician leverages what is of actual value to the service user and relies on more than one point, there may be a greater chance of bring about adherence to follow-up treatment [14].

Secondly, the varying willingness to accept the link between psychiatric treatments and metabolic syndromes leads to varying level of desire to preemptively prepare service users for the treatment of an eventual metabolic comorbidity. “Instead of being more curative than right now, we want preventative. Things are changing, but maybe not as quickly as we would like it to be” (service provider 0110). If the psychiatrist believes that psychiatric treatments do not cause metabolic symptoms, they are less willing to contemplate proactive steps to ensure proper follow-up for metabolic comorbidities.

### Strengths and limitations

Our study has several strengths: We obtained multiple perspectives on the issue of care preferences. The content reported in this study reached saturation. Theoretical sampling was used to recruit people who were non-compliant with their referrals to outpatient metabolic clinics. However, because the sampling protocol depended on attendance at mental health outpatient services, service users who were non-compliant with mental health appointments are under-represented. A rational patient framework was used to organize our findings, however, as noted by Corrigan and colleagues, such frameworks do not take into account the implicit nature of decision making, its emotional components, or the immediacy of some decisions [34].

## Supporting Information

S1 File Qualitative Data. This file contains the nodes used to construct the themes reported in the manuscript. Including advice to others; expertise; insight into illness; instructions; looking after kin; preferences; relapse; resistance to doctor’s orders; social factors; social support; stigma; therapeutic alliance; and uneasy about initiating treatment.
(ZIP)
