# Supplementary material for: The Impact of Stakeholder Preferences on Service User Adherence to Treatments for Schizophrenia and Metabolic Comorbidities
Source: PLoS One. 2016 Nov 16;11(11):e0166171. doi: 10.1371/journal.pone.0166171 (PMC5112999; doi:10.1371/journal.pone.0166171)
Supplement: S1 File — This file contains the nodes used to construct the themes reported in the manuscript. Including advice to others; expertise; insight into illness; instructions; looking after kin; preferences; relapse; resistance to doctor’s orders; social factors; social support; stigma; therapeutic alliance; and uneasy about initiating treatment. (ZIP) [file pone.0166171.s001.zip › Qualitative data/Advice to others with similar conditions.docx]

**Name:** Advice to others with similar conditions

**<Internals\\HDL interview 3 20160217171017621 no audio> - § 1 reference coded [5.62% Coverage]**

**Reference 1 - 5.62% Coverage**

Encourages people to get tested regularly and go to checkups, thinks people should get early treatment after the beginning of an acute episode.

**<Internals\\HDL Study - service user HDL_151224-0139> - § 1 reference coded [1.88% Coverage]**

**Reference 1 - 1.88% Coverage**

INTERVIEWER: is there anything that you like to tell us. To help us understand you experiences.

PARTICIPANT: yah, as people getting older right, they have such kind of diseases. So they have to take care of themselves and take medications often. And take…. Schizophrenia medications also. They have to take. Yah. And they don’t have to feel lonely at home, they can visit friends and talk to parents. Yah

INTERVIEWER: so if they take the medications, they will be well and can go out and do things.

PARTICIPANT: yes

**<Internals\\HDL study -Service user HDL_140211-0114> - § 1 reference coded [0.54% Coverage]**

**Reference 1 - 0.54% Coverage**

Sometimes when you want to advice to a person, like an individual for those who are career minded who are here because of stress, work related. You can highlight these issues to them you know. About erm.. to be treated is better than having relapse you know.

**<Internals\\HDL study service users HDL_151209-0140> - § 1 reference coded [3.04% Coverage]**

**Reference 1 - 3.04% Coverage**

INTERVIEWER: well, you.. I know this is how we .. it’s very important. Because I’m sure you understand you are not the only one who goes through this progression and if we understand how people like yourself experiences. We hopefully can help those who are now sort of developing the illness. And I’m sure in your circle of support, you must sort of tell sort of newly diagnosed people about a

PARTICIPANT: yes

INTERVIEWER: what to expect and what’s goes on. What sorts of things would you tell them about? To expect from the medical system. In terms of when you first see your physicians to get the pills or when you go for interviews, like that? Has there been any advice you sort of give..,

PARTICIPANT: I should .. ah.. for my own advice is health wise ah.. its, watch what you eat. What you eat and I mean do exercise, that’s all. That’s the simple thing. I’m not a.. I’ve experimental... I experiment on this trial, you know. On just exercise, you know. Food wise, you know. you should go internet, you know. But I can tell them that you know, you should eat, watch what you eat. You know. So exercise. Yah. that’s all.

**<Internals\\HDL study service users HDL_151209-0149> - § 1 reference coded [4.62% Coverage]**

**Reference 1 - 4.62% Coverage**

I: is there anything that you think would help people who don’t see that? That effect? Because we know that some people will take the medication for a while think that they are better and then stop taking the medication, they don’t realize that if they do they will have more symptoms. Do you think that there is anything we can tell them to help them learn what you have learned?

P: I think I can tell them my experience, like I have not had a manic episode since I was 19 , now I am 24 coming to 25, so it is best to keep taking the medication because it helps, because if I keep taking the medication and you see that I don’t have manic episodes you see it really helps, and I mean for my mum, I think I was diagnosed with clinical depression when I was 16 or 17, when the doctor told me to take medication, but my mother did not want me to take medication, because she doesn’t see me as a mentally ill person, she did not want me to take, but I think it is important to like let caregivers and people who have the illness to know that it is important to take the medication because that is the only way we can control 2144, because like if you are sick you have fever you have to take medication, if you are having mental illness you have to take medication, so yeah.

**<Internals\\HDL Study_service user HDL_151023_0035> - § 1 reference coded [3.54% Coverage]**

**Reference 1 - 3.54% Coverage**

INTERVIEWER: yah, but some people do feel that way. So, for these people right. Is there anything that you like to… in your experience.. is there anything you like to tell them?

PARTICIPANT: because I don’t really know what is crazy.. maybe crazy is.. only you are crazy, you are normal. Maybe it is that way.

INTERVIEWER: ok. and then about these people who have these condition like schizophrenia but they don’t want to come here to receive treatment like… is there anything from your experience that you like to tell that should they receive treatment.

PARTICIPANT: actually I also don’t know the cause for schizophrenia.

INTERVIEWER: has the Dr told you anything about it?

PARTICIPANT: Even if he tell me, I can’t recall. (Laughs)

**<Internals\\HDL Study_service user HDL_151218-0133> - § 1 reference coded [3.11% Coverage]**

**Reference 1 - 3.11% Coverage**

is there anything else you think is important for us to know about people with diabetes who come to IMH? Anything else you want to tell us?

PARTICIPANT: Doctor I don’t know about diabetes

INTERVIEWER: just about your experiences

PARTICIPANT: my experiences, this is the first time doctor that a doctor ask me if I have diabetes, first time. [Possibly referring to the present interview] my experience , I think to take care of food take care of drink, don’t take too sweet things, mind blood pressure , don’t take sour drinks. Don’t take, … what this drink [Malay word] don’t remember the name white colour and sour.

**<Internals\\HDL Study-Service User HDL_151203_0061> - § 2 references coded [2.24% Coverage]**

**Reference 1 - 1.32% Coverage**

Interviewer: Ok, so one thing we would like to find out is how people with schizophrenia and other conditions right? How are they managing their conditions, so if there is something that you can tell to them as an advice what would you tell them?

Participant: Nothing

**Reference 2 - 0.91% Coverage**

Interviewer: Ok, so you have not had any major problems down here? But is there something that you would like to tell those with your type of condition? Any advice?

Participant: No, no

**<Internals\\HDL Study-Service User_140210-0112> - § 2 references coded [9.37% Coverage]**

**Reference 1 - 4.95% Coverage**

INTERVIEWER: what would you tell someone who may have schizophrenia and who doesn’t yet have diabetes or high cholesterol to prepare them for potential diagnosis of this. Sort of, is there any information that you would like to have known about sooner?

PARTICIPANT: but then, if the person has secret excuse schizophrenia..

INTERVIEWER: (ack)

PARTICIPANT: erm, it might not be all these that .. that person don’t have diabetes and high cholesterol

INTERVIEWER: yup.

PARTICIPANT: so, just be prepared on.. like have to.. read more on the information. Like google through and maybe

INTERVIEWER: sorry, google through what?

PARTICIPANT: ah, google through the .. what they call it? About schizophrenia and all that la. Just be prepared for it. Ya. Even though even you are seeking treatment you can ask the psychiatrist to explain more further about your condition. Ya. And prepare for any future attacks.

INTERVIEWER: and that’s what you might tell someone who might have yet to develop diabetes or

**Reference 2 - 4.42% Coverage**

INTERVIEWER: cause one of the thing we have been trying to do is help people who aren’t as understanding as you. cause we have some people who come in with an attack and they don’t follow up. Which mean that they have another attack and then another attack. And it’s a .,. very disruptive for their lives. So, we were wondering if you have any advise you might wanna give to somebody who is reluctant to come in, so that they can get well or function a bit better.

PARTICIPANT: I will advise them to go for medical check up. I mean like for the psychiatric check up, we. For 3 months so how many months or so. Because I mean what’s the point of having like having attack every other day when you can prevent it from occurring soon. Like, after one attack you come back for .. follow up. Then, I’m sure that the attack would be lesser or either prevented la. It’s best to prevent then cure, they say.

**<Internals\\HDL study-service user_151210-0147> - § 2 references coded [3.03% Coverage]**

**Reference 1 - 1.49% Coverage**

INTERVIEWER: yes. What do you think makes you have those interests? What can we tell people who may be like you eventually. To help them. You think there is anything we can do? Say to …

PARTICIPANT: maybe trust them to get the cholesterol level know. So that they know which.. which stage.. which stage are they in.

**Reference 2 - 1.54% Coverage**

INTERVIEWER: ok. If we ask you about what we want you to tell yourself. Let’s say 20 years ago. On how to better manage your illness. Is there anything you want to sort of know back 20 years ago?

PARTICIPANT: have to take medicine regularly. That my advice

INTERVIEWER: that’s your advice, ok. Ok. Think that’s everything.

**<Internals\\HDL_CG 151023_0036> - § 1 reference coded [3.08% Coverage]**

**Reference 1 - 3.08% Coverage**

INTERVIEWER: or anything you would like to tell other caregivers who are actually taking care of people like him?

PARTICIPANT: oh I could say … patience [laughs] patience is needed. And you must understand that at times the love to throw tempers, like almost 24 hours, and they get easily angry for small little things. Quite tension, and yeah, so just be patient with them, yeah, not to be too confrontive, because f caregiver they don’t be patient it would be hard for themselves also, yeah. They will be stressed also, yeah.
