# Supplementary material for: The Impact of Stakeholder Preferences on Service User Adherence to Treatments for Schizophrenia and Metabolic Comorbidities
Source: PLoS One. 2016 Nov 16;11(11):e0166171. doi: 10.1371/journal.pone.0166171 (PMC5112999; doi:10.1371/journal.pone.0166171)
Supplement: S1 File — This file contains the nodes used to construct the themes reported in the manuscript. Including advice to others; expertise; insight into illness; instructions; looking after kin; preferences; relapse; resistance to doctor’s orders; social factors; social support; stigma; therapeutic alliance; and uneasy about initiating treatment. (ZIP) [file pone.0166171.s001.zip › Qualitative data/Expertise.docx]

**Name:** Expertise

**<Internals\\HDL interview 1 20151009144255082 no audio> - § 1 reference coded [9.24% Coverage]**

**Reference 1 - 9.24% Coverage**

Thinks it is better to get treatment for Schizophrenia at IMH and treatment for chronic conditions at the polyclinic as they are both skilled at treating the respective conditions; feels that if she comes to imh for her diabetes, they might not be able to understand her condition vice versa.

**<Internals\\HDL interview 2 20151111171452547 no audio> - § 1 reference coded [9.34% Coverage]**

**Reference 1 - 9.34% Coverage**

Does see the convenience of having treatment all in one spot, but prefers to see as he does, polyclinic and psychiatrist as the high blood pressure is routine and common and can be handled by polyclinic, but psychiatric conditions requires more experience “ when you have a condition like this, you prefer to be treated at a place that has treated people for quite some time”

**<Internals\\HDL interview 3 20160217171017621 no audio> - § 1 reference coded [7.14% Coverage]**

**Reference 1 - 7.14% Coverage**

Would go to polyclinic for both treatment of psychosis and cholesterol but has “no choice” but to come to IMH . doubted that polyclinic would have the right medication to give out.

**<Internals\\HDL study -Service user HDL_140211-0114> - § 1 reference coded [0.98% Coverage]**

**Reference 1 - 0.98% Coverage**

But I don’t know whether bedok poly can really prescribe the medication which you are giving. And then, they are actually more for chronic. And then I think.. even like cholesterol right. It’s actually detected here. But I think it would have been treated here and they would have done it here. But instead they said they refer me to poly then. Of course cholesterol was no issue now lah. But in terms of blood pressure, I don’t know if they combine into one kind of thing

**<Internals\\HDL Study Service User HDL_151209-0145> - § 3 references coded [4.69% Coverage]**

**Reference 1 - 1.64% Coverage**

INTERVIEWER: cause we are curious to know if people with both conditions could be managed in the polyclinic.

PARTICIPANT: oh

INTERVIEWER: so these are... there are more professionals and it might be possible that

PARTICIPANT: I don’t think there are more professionals in the polyclinic.

INTERVIEWER: no?

PARTICIPANT: no.

INTERVIEWER: what do you think?

PARTICIPANT: I think the.. they are all fake Doctors

INTERVIEWER: so you prefer seeing 2 specialist

PARTICIPANT: yah

INTERVIEWER: in special places

PARTICIPANT: yah

INTERVIEWER: for each condition

PARTICIPANT: yah

**Reference 2 - 1.51% Coverage**

INTERVIEWER: beyond, you know the.. so, you mentioned that it was difficult for you to choose. You din know who to choose. As sort of, if you get services from one place.

PARTICIPANT: no, you see. Cause I believe in specialisation. And I know one Doctor cannot specialise. They can manage the medicines. They call it … I forgotten the terms. They call a Doctor, who can examine both and see if there is any clash. So, I hope someone does that for me. But I don’t want one Doctor for both cases, because i don’t think that’s possible. Yah

**Reference 3 - 1.55% Coverage**

yah. You mentioned that you prefer Tan Tock Seng rather than polyclinic. What are the different types of services which makes you prefer Tan Tock Seng more?

PARTICIPANT: I think they are more professional there. the way they deal with diabetes condition.

2nd INTERVIEWER: ok, so. Because you mentioned that in polyclinic there was a dietician right?

PARTICIPANT: yah… but she wasn’t … that good la. She didn’t give me a good knowledge of food, you know. I only remembered like I say, her saying you go and doctorink diet coke or coke zero. You know.

**<Internals\\HDL study service users HDL_151209-0149> - § 3 references coded [3.10% Coverage]**

**Reference 1 - 1.00% Coverage**

so there is some stigma, that if you go to IMH a lot , people will have a lot to say but I don’t find anything at the polyclinic because I don’t think they know, but even if I were to tell them, I am ok with it, because I am sure that they will keep it confidential. Yeah.

**Reference 2 - 0.46% Coverage**

I: do you think that IMH, the psychiatrists here are adequately equipped to provide you with the care that you need?

P: yeah

**Reference 3 - 1.65% Coverage**

I: is there anything else in an idea setting where you could choose one or the other, you would think maybe a GP is better educated, different medication, anything like that?

P: sorry can you repeat?

I: that a GP may be better educated to treat, or have better access to medications to prescribe?

P: …. I feel like the IMH is … I feel that there is more… more facilities, is it the correct word? Whereas the GP doesn’t have that many services. Yeah

**<Internals\\HDL Study_service user HDL_151210-0137> - § 1 reference coded [3.16% Coverage]**

**Reference 1 - 3.16% Coverage**

INTERVIEWER: how about skills wise?

PARTICIPANT: skills wise?

INTERVIEWER: hmm (agree)

PARTICIPANT: in terms of what?

INTERVIEWER: for example you are seeing IMH for psychiatric condition right? Do you think that if you go outside at polyclinic you will get the same amount of care? Do you

PARTICIPANT: polyclinic is quite a bit crowded. And they like give me referral letter, they.. I call the hotline also, engaged. Keep on engaging. So.. and there’s no text message coming in to see Dr. They forgot to text me the appointment.

I:ok

PARTICIPANT: yah. Different polyclinic has different skills to handle. But I still prefer singhealth. Singhealth polyclinic.

**<Internals\\HDL Study-Service User 140208-0106> - § 2 references coded [8.44% Coverage]**

**Reference 1 - 5.38% Coverage**

INTERVIEWER: ok ok. Very good. How did you feel about this way of splitting of services. Would you rather have Jerome treat everything, or the polyclinic treat everything. How do you prefer?

PARTICIPANT: erm. I think , because Jerome is only a psychiatrist. He is not a general doctor. So, its… it’s very difficult for him to give me the high blood pressure pill. It’s not .. he don’t study in that area.. So totally cannot la. The polyclinic doctor is specialized in general medication. So they can give the.. they can treat my high blood pressure la. So different.. because the IMH is more specialized in mental welfare and also the polyclinic is more general health care. General.. ya..

**Reference 2 - 3.06% Coverage**

PARTICIPANT: No, I think it would be better to have.. the… the IMH giving me the medicine and as well as the medicine at polyclinic. Because the polyclinic doctor may not understand my condition ah. My mental condition. Because they are not specialized in it.

INTERVIEWER: so, you would feel more comfortable treated only at imh for the 2 conditions, for your hypertension and schizophrenia.

**<Internals\\HDL Study-Service User HDL_151203_0061> - § 1 reference coded [1.63% Coverage]**

**Reference 1 - 1.63% Coverage**

Interviewer: Nothing. Do you think the polyclinic doctor can treat you for schizophrenia?

Participant: Yes, yes

Interviewer: You think they can

Participant: Yah

Interviewer: Can you tell me about it?

Participant: Don’t know

Interviewer: You don’t know why they can treat you? Do you think they have the skills to treat you?

**<Internals\\HDL study-service user HDL_151209-0152> - § 2 references coded [1.69% Coverage]**

**Reference 1 - 0.61% Coverage**

INTERVIEWER: why? Because remember you said that MOs sometimes not very professional. Yah? At IMH

PARTICIPANT: the MO never made any decision because ah.. he can’t make the decision by.., he say next time he want to talk to me, this one no need to talk to me.

**Reference 2 - 1.08% Coverage**

INTERVIEWER: polyclinic better to.. because IMH focus on.. eh, you sure know one. Because of psychotic medication. So they want. They still got ah, they say is best advise to go to polyclinic and get the simvastatin. I eat the medicine, simvastatin. My medicine is simvastatin. Because polyclinic got problem the medicine. And then the .. polyclinic the Dr very professional in giving advise on like… refer you to dietician for the treatment, everything inside the.

**<Internals\\HDL Study-Service User_140209-0109> - § 3 references coded [3.52% Coverage]**

**Reference 1 - 1.09% Coverage**

INTERVIEWER: rather than coming to IMH. Would you prefer if your GP was the one to treat your mental illness?

PARTICIPANT: he know how to treat or not?

INTERVIEWER: he doesn’t know how to treat?

PARTICIPANT: ya.

INTERVIEWER: no?

PARTICIPANT: no.

**Reference 2 - 2.00% Coverage**

INTERVIEWER: ok.. Is there a difference in the way that they treat you?

PARTICIPANT: not say different la. As long as.. is you see them, you get the medicine you... your.. you recover is important la. The most important is you recover. Able to recover.

INTERVIEWER: and that important to them

PARTICIPANT: ya.

INTERVIEWER: ya, but they don’t differ in the way that they speak to you on one on one? Or listen to you?

PARTICIPANT: not much difference actually.

**Reference 3 - 0.43% Coverage**

PARTICIPANT: ermm… IMH the doctor is quite good. Expertise and the characters also good la. So..

**<Internals\\HDL Study-Service User_140210-0112> - § 1 reference coded [3.35% Coverage]**

**Reference 1 - 3.35% Coverage**

PARTICIPANT: so far.. I believe that I get a good treatment here. Ok because of the speciality of the department. Ok, for mental health. So, I don’t have a problem with it ah. Yah

INTERVIEWER: ok, suppose you go over to a polyclinic right? And you talk about your.. schizophrenia to your polyclinic doctor, do you think that he will be able to help you? give you proper treatment for that or ?

PARTICIPANT: I’m not so sure. Because of the fact that maybe they are specialised in only medical conditions and not so much on psychiatric conditions. So, I .. not really sure whether they are…

INTERVIEWER: capable of ..

PARTICIPANT: they can give me the proper treatment or not.

**<Internals\\HDL Study-Service User_140214-0118> - § 1 reference coded [0.76% Coverage]**

**Reference 1 - 0.76% Coverage**

Because the sickness that IMH treat is different from the sickness that polyclinic treat. They are different. Yah, they.. here is more specialised, mental illness. Because schizophrenia is like something uncontrollable that even I cannot tell myself, I cannot read myself.

**<Internals\\HDL study-service user_151210-0148 (chinese with english)> - § 3 references coded [7.56% Coverage]**

**Reference 1 - 2.74% Coverage**

2nd: 但是专科贵， 你觉的会比较好吗？ 还是。。(but its specialist… you feel that it will be better? Or..)

P: 没有啦。 跟这边的一样。 (no la. Same as here)

2nd : 跟这边的一样? (same as here?)

P: hm.

2nd : ok.

P: 有专没专都一样。(have specialist or no specialist also the same)

2nd : 什么?(what?)

P: 有专科跟没专科都一样(have specialist or no specialist also the same)

2nd: 为什么?(why?)

P: 他们都一样吗。 他们的检查那个的胆固，那个糖尿病， 每次一样的。看专科也比较贵。 要百多块。 (they all the same, they check the cholesterol, diabetes. Everytime the same. Specialist more expensive. Need 100 plus dollars.)

**Reference 2 - 1.44% Coverage**

Ｉ:　but do you think that the specialist might have better knowledge and maybe better able to easier to help you or? Do you think it’s the same? Same ability to help to you, as here in IMH?

Ｐ:　here also also take same medication ah.

Ｉ:　so it’s also the same medication?

**Reference 3 - 3.38% Coverage**

2nd: 但是你觉的可能好像他们懂的比较多吗？还是你觉的 (but do you feel that they will know more?)

P: 　专科也是懂的比较多。　(Specialist will know more)

2nd: ok. 还是你觉的跟这里没有分别？　(ok. But will you feel that it there will be any difference compared to here?)

P: 　你看。　专科比较。。　查得比较明确。　因为这边他。。　oh 你有diabetic, 吃药！(you see. Specialist will be better. Checking will be more detailed. Because here.. oh, you are diabetic, take medicine!)

2nd: 你之前有去专科？(you been to a specialist before?)

P: 　没有没有。我本来是看这边的。　因为医生这边看也是不错。　但是等很久。　有时候有很多人，　要等很久。　(no no. I see here originally. Because the dr here also not bad. But wait very long. Sometimes a lot of people, need to wait very long).

**<Internals\\HDL_CG 20151105 notes, no audio> - § 2 references coded [23.89% Coverage]**

**Reference 1 - 17.39% Coverage**

Prefers Tx in one spot, but spoke to IMH who said “not possible to treat chronic conditions” and spoke to polyclinic who noted that it was “not advisable to get treatment at IMH for chronic condition” . Family would also prefer Tx in one spot. But would only want Tx for chronic condition if physician was trained and competent. Choice depends on qualification of physicians.

**Reference 2 - 6.49% Coverage**

Always sees different physician and is therefore worried about each physician psychiatrist not being able to do enough to get to know him.

**<Internals\\HDL_CG151209-0142> - § 5 references coded [10.07% Coverage]**

**Reference 1 - 3.64% Coverage**

PARTICIPANT: first time, for how long? Maybe 3 weeks, I think. About 3 weeks, they changed the meds, changed medicine, changed medicine

PARTICIPANT2: also because [previous psychiatrist] resigned, go to private practice, so we cannot afford private practice, so we came to IMH, under IMH they changed to periperidone?

PARTICIPANT: no no other medicine

PARTICIPANT2: but after a while they gave that medication to her

PARTICIPANT: they change few medications

PARTICIPANT2: change change change and after that they gave her that medicine because it is a new drug under research, something like that.

INTERVIEWER: so you were part of research back then too

PARTICIPANT2: correct, so after that period of research ended , then we need to pay I think , but then they gave us sample so we don’t pay, but in the end it didn’t quite work.

PARTICIPANT: I can’t afford to pay all these things because I am broke already

PARTICIPANT2: so if it doesn’t work they change medications, I think mental patient they adjust the dose, change and try to get the right combination you know

PARTICIPANT: and then in 2012 she admitted again for about 3 months 3 whole months, yeah, until today, I think 2012, the doctor say , he say he want to do ECT for her, so we say can you try oral, because it is not proven that ECT can help so we asked can you have oral sample then the doctor say we can try [incomprehensible] so from then until today

**Reference 2 - 1.50% Coverage**

PARTICIPANT: oh the people there are not much experience about mental people, so… not like the normal people, no say not helpful but not experienced, nurse her are different.

INTERVIEWER: this is a difference we are very interested in can you tell me how that sort of , how you know these things what examples you have ? or how you know they are not experienced?

PARTICIPANT: because these people..2926 these type of patients they have not patience, they cannot wait too long or whatever, even when come to the hospital if they wait too long the will go away, they do not take the time

**Reference 3 - 3.09% Coverage**

INTERVIEWER: do you think, if we talk about IMH vs. poly, do you think one place is better for her to get care for her high cholesterol? Because if she is only willing to go see few doctors, do you think it would be better for her to come see the IMH doctors and get everything treated by the IMH doctors, or to see the poly doctors?

PARTICIPANT: I think IMH better, one place better

INTERVIEWER: why

PARTICIPANT: because if two places, different places, so when she come here she no worry, she learn to go this place and that place

PARTICIPANT2: I think for metal patient somehow they have attachment, if they are used to this place the routine is there they more or less think “ok I am coming here”

PARTICIPANT: the hospital doctor here

PARTICIPANT2: it is a routine, but if we have to go to another place, sometimes they will feel “why am I seeing doctor so often” and then “You do I go to this place”

PARTICIPANT: angry

P2:”you go to see la” but here at least they say ok, sometimes they accept that they have mental illness , they accept that they need the medicine, but if “now you tell me that I got another disease I need to see another doctor in another place! You go la I am not sick, I am well!”

**Reference 4 - 0.70% Coverage**

PARTICIPANT2: the reason if it is the same place everything is ok, even if it is manageable and you don’t need the specialist!

PARTICIPANT: they can’t tell the difference, see doctor and at the same time take blood , very convenient, doctor say go take blood test convenient

**Reference 5 - 1.14% Coverage**

we can’t expect the doctor to treat everything, he is not trained but he can help to manage because he is the doctor, whereas we only know our own trouble our own inconveniences, but the doctor can be a better person to help us manage, at a manageable level la, but if it is beyond then of course we cannot demand that this doctor continue to help me, you know “I want you to do my heart surgery here” when you don’t have the facilities, you know?

**<Internals\\HDL_CG151209-0158> - § 1 reference coded [1.53% Coverage]**

**Reference 1 - 1.53% Coverage**

INTERVIEWER: now, do you worry about the doctors here being less knowledgeable, educated to give those types of medications? Or do you think that there is equal level of skill?

PARTICIPANT: I mean equal level of skill for medication, I mean, I think cholesterol, it ok here or there, but for schizophrenia I think here better , the case, where we can talk to the person,

**<Internals\\SP 140130-0095> - § 3 references coded [6.56% Coverage]**

**Reference 1 - 3.55% Coverage**

the challenge my personal issue with this is that if we ask the psychiatrist to do this besides the advantages of yes they get treated they get their medication which is probably better than not, we have 2 problems: one you dilute the psychiatrist focus if we then treat the whole thing of course it is great for holistic medication but nobody does everything well . and if you the psychiatric issues are significant enough and complex enough that we in all honesty we should be spending more time sorting that out not all patients have their issues… psychiatric issues in complete remission. in fact many of them still have multiple issue and resolute problems that require treatment. And the other reason is that we are not, we are really not the best at diabetes, hypertension and …er hypercholesterolemia are not simple conditions you have an entire specialty devoted to that sort of thing endocrinology...and even gps do it my wife is a gp she knows what…her field, their knowledge of these 3 conditions, she does so much of it in the polyclinics it’s really different from me my questions are what medicine should I give them? And she’s asking what is a metabolic risk factor? What’s your family history like? What’s your diet like? what is your weight profile? Er… I don’t know, what medicine do I give them? So I like when people speak psychiatrist, What’s the best medicine for depression? What type of depression so it’s different when I speak to my wife about this and she always sighs when I talk to her about what is the best thing to do…what I do…because I don’t ask the right questions, I don’t have the right information same situation when we see patients with mental disorders being treated by gps sometimes they do well, those that don’t often it’s an issue of lack of knowledge and training that’s all. They can give exactly the same medication that we can sometime their therapeutic relationship is better than we have, but it’s often an issue of knowledge and training and to… I see the advantage of getting patients treated psychiatric patients with metabolic comorbidities is there an easier term to summarize this one? er never mind

**Reference 2 - 1.30% Coverage**

These patients if there is a I can see the advantages at least if they come here and we treat them…the medications we monitor their blood pressure and cholesterol we do all that for them versus no treatment at all because of course they won’t see a polyclinic because you have to pay money which you don’t have the obvious advantage of that, the disadvantages are less clear because we will divest our time away from psychiatry because our time is limited and the patients are going to get less or unless they have a dedicated clinic, which is a different issue. If we ask the psychiatrist to do that focus is taken away from a severe mental illness which is difficult enough to treat and we don’t it as well as we should and that’s my initial first thought lots of possibilities from here

**Reference 3 - 1.71% Coverage**

I also recognize the advantages of seeing someone who is more skilled in that area polyclinics are generally nearby and a significant proportion of these patients don’t work so they actually have the time to do this and I suppose the therapeutic relationship with them helps “look, this is best for your care go and see them I can’t give you this medicine I’m not a specialist in this” so it works for most patients so the short answer is er it is a difficult question to answer because of selection allocation bias I do this I treat small number of my patient don’t know how many but small and they are very happy with this they don’t go anywhere else a lot of patients I push out some of them will end up seeing the primary doctor and they will get better care for example, they will get their DNAs done, their diabetic retino therapy, eye checks done, they will get their feet check by podiatrist we don’t have that service here, I can’t order it so they don’t get … they will get proper care there, some may end up getting worse care…

**<Internals\\SP_140109-0130> - § 2 references coded [5.97% Coverage]**

**Reference 1 - 3.47% Coverage**

ok and so, it is our responsibility to monitor, and is it our responsibility to treat? Or once we have found the problem, we would want to refer to polyclinic or GP outside?

PARTICIPANT: ok, uhm there is no direct answer to it, the general rule is polyclinic has better set up to manage condition, but there are exceptions, like what I say some may not engage so well with polyclinic, that is where we have a role as psychiatrists to come in and um provide that kind of support. In what my general principal is I would initiate them first stabilize that is what I mean, put them on the treatment first and then transition them to the polyclinic, so that it becomes smoother rather than just throw to the polyclinic and the patient gets the shock of , and don’t know what to do at the polyclinic which is a different environment. Yeah.

**Reference 2 - 2.50% Coverage**

I probably believe that one of the reason is that not everyone has the same training and not everyone has the same exposure in the treatment of the medical conditions , and that makes a difference in their comfort level, how they are comfortable managing these conditions and whether you are worried you may be missing something. Yeah.

INTERVIEWER: so your exposure to these conditions has led you to feel more comfortable initiating and then referring

PARTICIPANT: yes, and even managing if necessary it is just the problem is we do not have the whole set up of the facility, that is my only concern

**<Internals\\SP_140116-0079> - § 1 reference coded [3.86% Coverage]**

**Reference 1 - 3.86% Coverage**

**Do you think that on this issue er maybe it’s better to…do you think the care that they get in one setting or another is superior to one or the other?**

**PARTICIPANT: As I said I don’t know for dyslipidemia I think it’ not…I…I…I’m not…at least from what the patients report to me I don’t see it being very superior.**

**Interviewer: OK**

**PARTICIPANT: Yah…er… but I think if its er… high blood pressure or diabetes I think they get a better monitoring done at a polyclinic at present**

**Interviewer: And so that’s your view of the way things are(20:51)**

**PARTICIPANT: And it really depends it is based on what the patients have told me**

**Interviewer: And what do you think contributes to that superiority there… is there anything that we can say to patients who are abit hesitant and say ok... well you know…at this setting people have found xyz that makes that…better?**

**PARTICIPANT: I guess, I think they have a diabetic care plan or hypertensive care plan in the polyclinics so these kind of chronic diseases… and in fact the other day I was talking to a doctor who was in the polyclinic. So it’s again a team based approach so they will be seen by the advanced practice nurse, the nurse, the dieticians and then the doctors will see and I think that approach I think the patients do benefit from it**

**<Internals\\SP_140120-0082> - § 3 references coded [4.63% Coverage]**

**Reference 1 - 2.49% Coverage**

And so that’s sort of…those nudges to get them together, simply helps them to remember they’ve got to come for…(15:36) Do you think that the care that patients get in one setting, like IMH, differs from the setting like in the community? In terms of…

PARTICIPANT: For…medical conditions?

INTERVIEWER: Yeah.

PARTICIPANT: (15:53) I would like to think that maybe for the medical conditions, they are probably getting more optimal care there (16:02) than here – because here, like what we’ve been saying, people are not overly familiar with the treatment guides. So would that mean that the treat – they may affect the treatment outcomes, I think; whereas in the community, they have the specialist in managing some of these chronic medical conditions. So I would think possibly in that setting, the doctors there should be more familiar. Outcomes I would think should be better

**Reference 2 - 1.36% Coverage**

But at the end of the day, it’s you’re psychiatrist – 99% of the work we’re doing is psychiatry, and you can read a lot from journals, you can read a lot from books, but I’m not sure if that is sufficient - is real life enough for improving the medical care of our patients? If they are already experts out there – having guidelines, suggestions, protocols they’re using along the way, then why not make friends with some of them, and just copy and paste some of their system over?

**Reference 3 - 0.78% Coverage**

Because if you’ve got patient who is extremely disturbed, they are not – some of these regional hospitals are not capable of managing…these patients. And (34:40) no choice, they have to be sent here. And once they are sent here, the follow-up will naturally be here as well.

**<Internals\\SP_140120-0083> - § 2 references coded [1.81% Coverage]**

**Reference 1 - 0.67% Coverage**

i think the 3rd thing is about unfamiliarity with the condition and also erm inadequate training so they may not be able to I mean they may not be confident they may not kind of er be aware about how to pick up early relapse and things like that…

**Reference 2 - 1.14% Coverage**

I mean I must say you know for er lay person er often there is this view that you know seeing a specialist because this specialist has more knowledge so at the first cut I will want to have a specialist make that assessment and diagnosis it likely to be more accurate and you know he’s probably also likely to be more well-versed with er current treatment options and you know and so someone to have that discussion with.

**<Internals\\SP_140123-0081> - § 1 reference coded [2.63% Coverage]**

**Reference 1 - 2.63% Coverage**

I think the the the again I just want to emphasize my general principle to most people, I mean to my junior to my when I do supervision with them is that… know your limit, know your limit. Do not be overtly gung ho I think if you are competent you should be able to give them the service, you’re not please refer to someone who is better at giving the service be it in the community or elsewhere. There is always er no one will fault you for doing that because in the end you have your best intentions. I give you another example, for example in the night one of our patients in the chronic ward have some condition, be it fracture or fall. If we can manage it, we will manage it over here, if we can’t we have to send the patient out . (58:22-58:24) just because here our doctors on duty at night but our doctors are junior doctors (58:29) and our consultant on call is only over the phone, if we can provide the service we provide the service, if we can’t we should know our limitations, we should refer out. An example I give just now is the cut around the eye, you have to send the patient out I think you are doing the patient totally a dis-service if you’re there trying your best to stitch the area and at the end of the day the patient has some form of double vision or visual impairment just because of your super-ego

**<Internals\\SP_140123-0084> - § 2 references coded [1.87% Coverage]**

**Reference 1 - 0.64% Coverage**

If you really wanted to…yes there is expertise. And there are also some gp er… gps who run clinic here as well so there’s doctor Alvin lum, he is a gp who runs clinics here so if you can we have access, yes we can email any of these doctors or gps or medically trained erm…to get some advice

**Reference 2 - 1.22% Coverage**

Other than expertise…I don’t…I guess the only, no…I think it’s mainly the expertise the one benefit of having erm like I mentioned before the psychiatrist and the person managing the medical comorbidities really liaising closely is that if say the medical co-morbidities, for example cholesterol is still erm not responding to medication, diet, lifestyle change, then that they would then be able to speak to the or liaise with the psychiatrist to say well can you change the antipsychotic medication that’s causing this or might be contributing to it.

**<Internals\\SP_140123-0085> - § 3 references coded [2.15% Coverage]**

**Reference 1 - 1.08% Coverage**

Like how confident the physicians in terms of looking after managing these chronic illnesses, because psychiatrists are trained in psychiatric field per se lah. So some of the physicians might not be so confident to look after this issue. Especially these crisis issues. I would say that if the doctors are very well-trained in this field also, then it would be a good idea for us to manage this issue if we can (17:46).

**Reference 2 - 0.27% Coverage**

Because at the end of the day we are dealing with their mental health. We are more specialised in this way.

**Reference 3 - 0.80% Coverage**

Of course I would like…prefer my family members to be treated by the doctors who are specialised in the different areas lah. Because at the end of the day, you are well-trained, you are the specialist in that area. Because we will feel safer for the patients to be seen or under the care of that specialist team.

**<Internals\\SP_140125-0086> - § 1 reference coded [0.84% Coverage]**

**Reference 1 - 0.84% Coverage**

if it is simple treatment I think why not we can do it but if it is something which is more complicated like the patient need to titrate insulin for example in serious cases or there is already liver involvement if the lipidemia is that bad or the patient develop some kind of side effect to the drugs then I think we are not the best place we are not trained for that yah

**<Internals\\SP_140125-0087> - § 2 references coded [3.49% Coverage]**

**Reference 1 - 1.78% Coverage**

I think not many people do the baseline fasting bloods, and even the , hardly anybody does the 3 to 6 months fasting bloods, and very little do the annual test as well, so I think we are not monitoring sufficiently, I think there is not enough awareness as well, and … even when the results are abnormal I think generally psychiatrist, they are not very well versed in treating medical comorbidities, and so sometimes it has not been managed as appropriately as it should be.

**Reference 2 - 1.71% Coverage**

I think the nursing care as well, because nurses here are psychiatrically trained so they are the best in the world in terms of handling and educating impatient, restraining the patient de-escalating patients, but compared to nurses in restructured hospitals, really I think the medical knowledge is a little bit lacking. Uhm knowing when to highlight certain medical issue to the doctors, things like that, I think the nursing part needs to be beefed up too.

**<Internals\\SP_140126-0088> - § 3 references coded [5.19% Coverage]**

**Reference 1 - 1.86% Coverage**

i feel that for conditions like hypertension diabetes, lipidemia uhm we have a very effective system of monitoring in our polyclinics, so they have a system of blood monitoring every few months and a system of escalation, they are probably more familiar because they manage more of the hypertension diabetes, lipidemia problems in their daily work load, so they may be more up to date, with the pharmacology of such medications as well as potential other interactions with drugs , for example.

**Reference 2 - 1.50% Coverage**

sometimes they are persuaded, like if they are new diabetes onset, they are persuaded because we convince them that there is a system of care, which primary care offers a certain standard out there, and being higher volume and higher volume is more experience, and with that they can probably get better care for their medical condition than from us since we are a tertiary care psychiatric facility.

**Reference 3 - 1.83% Coverage**

my sense is that the skill set for GP and polyclinics for treating psychiatric outpatients are much less than our skillset in treating hypertension diabetes, lipidemia, because most of us would have gone through postings , even before entering general psychiatry when we have to treat such conditions, but those in primary care would not have gone through a psychiatric rotation

INTERVIEWER: uh hum, so just universally psychiatrists have that foundation for medical comorbidities?

**<Internals\\SP_140202-0098> - § 2 references coded [1.90% Coverage]**

**Reference 1 - 1.62% Coverage**

I mean the basics of medical education have been well laid out in our5 years of undergraduate but the basics of psychiatry probably have not been dealt with as much because we only start in I think year 4 and you know it’s very little time to go through such a wide field whereas medicine is the basis of our degree and that is kind of inculcated throughout the way and kind of every doctor who is worth his (32:56) know at least how to manage basic metabolic complications so if you ask me to pick a choice I would say that it will probably still be better for a psychiatrist to manage both and to you know and be aware of how to pick up all these early warning signs

**Reference 2 - 0.28% Coverage**

So so I do think that I would want the patient to be managed by a doctor that I know is quite reputed in that field

**<Internals\\SP_140203-0100> - § 2 references coded [2.48% Coverage]**

**Reference 1 - 1.44% Coverage**

more complicated stuff we will ask them to attend polyclinics, especially when it becomes more complex regimes, or they require yearly follow-ups with a dietetic nurse, and things like that , the preference would be that they see someone for their physical health, we are also short of time, we do not have enough time to look at both mental and physical aspects of health in that way they are better served by seeing someone who is exclusively for let’s say diabetes or whatever health condition they have.

**Reference 2 - 1.04% Coverage**

sometimes people have difficulty in terms of expressing themselves, they are not asked questions in the right way, like we are more skilled in terms of communicating with people who have a mental illness, especially people with chronic schizophrenia, they feel quite out of place going to such places, they would rather just come here and get everything done here.

**<Internals\\SP_140214-0116> - § 2 references coded [3.17% Coverage]**

**Reference 1 - 1.99% Coverage**

And once you have referred someone to the polyclinic, and the polyclinic has chosen to start a type of treatment, are you comfortable taking up the follow up of that treatment?

PARTICIPANT: for the follow up of the metabolic syndrome?

INTERVIEWER: yes

PARTICIPANT: no I think that is best place to treat these conditions the polyclinic

INTERVIEWER: why

PARTICIPANT: they are more experienced to handle these metabolic symptoms

**Reference 2 - 1.18% Coverage**

I think so far so good, I think that for me, I prefer leaving it to the GP to handle the metabolic symptoms

INTERVIEWER: because they have more experience

PARTICIPANT: or specialists, they are in the general hospital, specialist for metabolic symptoms.

**<Internals\\SP_151007-0097> - § 1 reference coded [1.51% Coverage]**

**Reference 1 - 1.51% Coverage**

psychiatric doctors definitely because of our backgrounds and d training, we ultimately, end up being much more skilled in treating mental health illnesses, and also no doubt start lagging behind in maintaining and developing our skill and competence for treating medical problems, no doubt about that, I think that is… expected… yeah.

**<Internals\\SP_151210-0132> - § 1 reference coded [1.69% Coverage]**

**Reference 1 - 1.69% Coverage**

so you can see that the way we think and the way they think are not the same, we are thinking that basically clinical care might be better in the clinic that has got a set up to deal with large numbers of patients with metabolic health problems, they have treatment guidelines , algorithms they have the medications they have got regular testing and they’ve got allied health podiatrist, optometrists, to do that work, and we think that that means it would be the likelihood of better clinical outcomes is there.
