# Supplementary material for: The Impact of Stakeholder Preferences on Service User Adherence to Treatments for Schizophrenia and Metabolic Comorbidities
Source: PLoS One. 2016 Nov 16;11(11):e0166171. doi: 10.1371/journal.pone.0166171 (PMC5112999; doi:10.1371/journal.pone.0166171)
Supplement: S1 File — This file contains the nodes used to construct the themes reported in the manuscript. Including advice to others; expertise; insight into illness; instructions; looking after kin; preferences; relapse; resistance to doctor’s orders; social factors; social support; stigma; therapeutic alliance; and uneasy about initiating treatment. (ZIP) [file pone.0166171.s001.zip › Qualitative data/Insight into illness.docx]

**Name:** Insight into illness

**<Internals\\HDL interview 2 20151111171452547 no audio> - § 1 reference coded [2.11% Coverage]**

**Reference 1 - 2.11% Coverage**

Does see that people may be a risk to themselves and others if they do not take meds.

**<Internals\\HDL study - service user HDL_151210-0153 (chinese)> - § 1 reference coded [0.30% Coverage]**

**Reference 1 - 0.30% Coverage**

P: 吃药、 没有去住院就算会康复咯。 (eat medication, no hospitalization is considered recovered)

**<Internals\\HDL study service users HDL_151209-0140> - § 3 references coded [2.19% Coverage]**

**References 1-2 - 1.07% Coverage**

So right now I don’t have any relationships because of the incident happened, you know. Because I get relapse, because of her, you know. Sometimes she.. you know. I’ve been maintaining myself as a single la. Obviously. I can’t have a relationship, only I have friends la. Yah. Because of my emotions, sometimes I have to… you can’t 100% know, you know. You have to give in. Then you break up, yah.

**Reference 3 - 1.12% Coverage**

How did you think your life changed when you sort of had the diagnosis.. understood that you had that mental illness?

PARTICIPANT: Think when I understand that.. when I got the last relapse of me..mine, in 2012. I went to stepping stone for the 2nd time. My first time was in 2010 and 12. Then they actually guide you along because the… they tell me about schizophrenia a lot. They tell a lot, teach a lot of things.

**<Internals\\HDL study service users HDL_151209-0149> - § 3 references coded [14.23% Coverage]**

**Reference 1 - 1.78% Coverage**

I: what made you choose to prioritize IMH scheduling vs. the polyclinic scheduling?

P: hum… I think because if I don’t have medication for my bipolar , my condition will get worse, and it will effect a lot of things, because once I get sick I will take time to recover, and then the, for this depression I had to take 1 year plus to recover, yeah. So I think for me, metal health is more important.

I: so that aspect of health has a greater impact on your ability to function.

P: yeah

**Reference 2 - 5.23% Coverage**

P: I feel…I feel happy that she wants to protect me, but also I feel like… there will come a time that I will, that someone will be prejudice against me because of my mental illness and I have to deal with it, and what is important is not what they think but my mental health, that is the important thing, so I think that maybe if she just don’t care about what other people think and focus on my mental health, that is the important thing. It is important to me.

I: that is important to us as well. And … so I am curious, because we don’t have a lot of people to speak to, with this level of detail [insight], so I hope you don’t mind if I as you this type of question, but do you think that her worries of having the family judged may compromise the level of care you get because she may want to sort of not do one thing or another?

P: I think she will , like maybe her family members will find out , maybe I have a feeling she will tell them like explain to them what is going on instead of assuming what’s my problem, yeah, so I think she will just talk to them, and then if things get worse for me, I think she will just not talk to them, if they judge me or my mental illness problem, so I think just, I think she will just talk to her family members, but I don’t think she will like…how do I say…I don’t think she will try to cover up my illness even more, she will be just “people found out, it’s ok, I’ll just explain” , yeah.

**Reference 3 - 7.22% Coverage**

I: how do you think you have changed as a person? How did you change in terms of the way you see yourself now compared to when you were first learning about taking the medications to control your bipolar episodes? Do you think you have changed in the way you understand the medication, you understand taking the medication? Or is it still not too sure about some things?

P: I feel like when I take the medication it controls my moods, so it is life a good thing, because before that I had no medication so it just goes crazy, but my mood will go up and down, a bit crazy, so after I take the medication I realize I have to keep taking it to control.

I: so you have realized that it does help you control?

P: yeah

I: is there anything that you think would help people who don’t see that? That effect? Because we know that some people will take the medication for a while think that they are better and then stop taking the medication, they don’t realize that if they do they will have more symptoms. Do you think that there is anything we can tell them to help them learn what you have learned?

P: I think I can tell them my experience, like I have not had a manic episode since I was 19 , now I am 24 coming to 25, so it is best to keep taking the medication because it helps, because if I keep taking the medication and you see that I don’t have manic episodes you see it really helps, and I mean for my mum, I think I was diagnosed with clinical depression when I was 16 or 17, when the doctor told me to take medication, but my mother did not want me to take medication, because she doesn’t see me as a mentally ill person, she did not want me to take, but I think it is important to like let caregivers and people who have the illness to know that it is important to take the medication because that is the only way we can control 2144, because like if you are sick you have fever you have to take medication, if you are having mental illness you have to take medication, so yeah.

**<Internals\\HDL study_ service user HDL_151023_0040> - § 1 reference coded [1.65% Coverage]**

**Reference 1 - 1.65% Coverage**

INTERVIEWER: and how did you come to the conclusion that it was sort of you have to accept it? In the beginning, do you find it difficult to accept?

PARTICIPANT: In the beginning, I was in denial. I din accept that I had the illness. But everybody keeps pushing me back here. If I miss my appointment, I get a relapse. Come back here again.

**<Internals\\HDL Study-Service User HDL_151203_0061> - § 1 reference coded [1.97% Coverage]**

**Reference 1 - 1.97% Coverage**

: But you are not taking anything for it. Do you go to the polyclinic?

Participant: Yes

Interviewer: Why do you go to the polyclinic?

Participant: Dunno my father take me there

Interviewer: You father takes you there… but you go and see for? do you know what you are seeing for at the polyclinic?

Participant: No

Interviewer: What does the doctor down there do?

Participant: I don’t know

**<Internals\\HDL Study-Service User_140113-0128> - § 1 reference coded [1.39% Coverage]**

**Reference 1 - 1.39% Coverage**

he din change your medication because of the results or ?

PARTICIPANT: change the medication for cholesterol or?

INTERVIEWER: no. for the antipsychotics.

PARTICIPANT: no. because abilify has been working well for me. So, I don’t really dare to change to another medication because it will go through the whole process of maybe. I’m not sure but.. I’m already comfortable with abilify.

**<Internals\\HDL Study-Service User_140214-0118> - § 1 reference coded [1.21% Coverage]**

**Reference 1 - 1.21% Coverage**

PARTICIPANT: Yes, so I learned from hougang care centre because they have sessions on anger management, you know. Things like being very careful and all that. The sessions that they hold, its helpful to us. For IMH, I think the DRs are concerned about us. That’s why they want us to come for regular checkup. Regular maintain, rather dosage maintenance. So, when we complie to them also, so that it help us, we help ourselves also can.

**<Internals\\HDL study-service user_151210-0147> - § 3 references coded [4.36% Coverage]**

**Reference 1 - 1.82% Coverage**

INTERVIEWER: do you know if your relapse is.. Happened because you stop taking your medications? Or was it simply because of the flu this time?

PARTICIPANT: because I stop my medications

INTERVIEWER: oh, ok. Because some people don’t like taking the medications.

PARTICIPANT: yah

INTERVIEWER: why did you stop? It’s ok if you did, its ok.

PARTICIPANT: I think I’m ok, so I stopped

**References 2-3 - 2.54% Coverage**

INTERVIEWER: yah. I think that’s something a lot of people experience. Do you think that it’s going to be reason for you to stop your medication again?

PARTICIPANT: no.

INTERVIEWER: no, why not?

PARTICIPANT: dare not already. After I get relapse again. I never stop anymore.

INTERVIEWER: you don’t want to what?

PARTICIPANT: never stop anymore

INTERVIEWER: you don’t want stop your medication anymore.

PARTICIPANT: yah

INTERVIEWER: no. So you have a clear understanding that there is a …that it does affect.

PARTICIPANT: hm

**<Internals\\HDL study-service user_151210-0148 (chinese with english)> - § 1 reference coded [4.81% Coverage]**

**Reference 1 - 4.81% Coverage**

2nd: really? 那你之前你不是有讲说你有进出医院咯。 (then previously you said that you in out hospital a lot of times?)

P: 对， 一直进出IMH医院。 (yes, in out IMH hospital).

2nd: 为什么？ (why?)

P: 因为听到声音。弄到我整个。。 那时候我整个很疯溃。 好像xiao cha bo 这样。 好像发神经这样。Then 不可以。 一直进出进出，进出很多次。 (because hear voices, ,make me totally… that time totally crazy. Like a xiao cha bo (chinese word for crazy girl). Like crazy. Then cannot. In out in out, in out a lot of times)

2nd: 是不是你没有吃药？ 所以才进出进出？( is it because you did not eat medications? That’s why in out?)

P: 对， 那时是没有吃药。 (yes, that time did not eat)

2nd: 为什么没有吃药？(why did not eat?)

P: 因为他讲不要吃比较好。(because he say don’t eat better)

2nd: 谁讲不要吃比较好？谁讲的？(who said don’t eat better? Who said?)

P: 我自己讲的。(I said)

2nd: 你自己讲的？(you said?)

P: 啊。 不要吃药。 (ah, don’t want to eat medications)

2nd: 那改次还敢吗？(and the next time do you still dare?)

P: 不敢了。不敢了。( don’t dare . don’t dare)

2nd: 不敢了啊？(don’t dare?)

**<Internals\\HDL_CG 151023_0036> - § 3 references coded [8.72% Coverage]**

**Reference 1 - 3.52% Coverage**

uhm not yet because I haven’t seen, because at ties I come with him and I realize that if I question the doctor, he will be quite sensitive and tense, like “why are you asking the doctor so many questions?” so at times I only ask questions that are important, because I am afraid he is a bit too sensitive, like him getting angry over me asking too many questions. Then uhm that is why I was saying that he is not aware of his condition, he should actually ask the doctoral, if he is well he will know that this problem, he should ask the doctor what can he do about it, why is this the medication? Yeah

**Reference 2 - 1.00% Coverage**

His mind have to change more, like I don’t know if the doctor can help him change his mind, his mind to let himself know himself deeper, like what condition he is facing.

**Reference 3 - 4.21% Coverage**

yeah make him understand and how is he going to like , uhm/.. Like make him... understand deeper, and how to let him help himself, alter himself to try and reduce the medication and be back to normal again.

INTERVIEWER: so in that sense, have you tried to help him?

PARTICIPANT: i… because the only think I can help him is , I give him advice, like be happy, be content for what you have now, you have nothing to stress about, no need to feed the house, no need to pay rent, just earning money to support your own self , and you can buy whatever you want. The only thing I tell him is I am making his room more green, yeah. Other than that I think there is nothing else I can do, the rest he has to work on himself. Yeah.

**<Internals\\HDL_CG151210-0163> - § 1 reference coded [7.19% Coverage]**

**Reference 1 - 7.19% Coverage**

INTERVIEWER: that’s very good, you also mentioned that she learned that taking the medication from IMH helps reduce the feelings, but you as a family have also noticed. How did you learn about that effect? Because some people don’t like taking the mediation because of side effects and they don’t understand that tit has an impact on their symptoms, how did you come to learn about this?

PARTICIPANT: I think it is through experience like what I mentioned, like when I ask her like now when she is stable I ask her “do you can you recall what happened last time when she likes to spend “ she say “yeah” now she is stable when she recalls she laughs, and say “yeah I recall” and she laughs it out, and then she said that it is not a negative experience she said she also doesn’t know why she does that , bo probably it is beyond her will power to do that , but that actually happens. And then they will question her, but she is very angry and aggressive always scolding us, scolding the taxi driver scolding anyone, she meets. And then we ask can you recall that happened, she will 50/50 can recall cannot recall and then she is feeling sad about it sorry about it and if she manage to recall she will apologize for her actions , so probably all this, she knows that when she is at her down or when these things very much affects her it affects us as a family, so probably because of this it makes her want to be controlled herself, or to continually take medicine so that she doesn’t cause trouble to her children and all that. And then when , because she doesn’t like the environment, like when we send her to IMH to be warded a few times, and she did share her experience, like “this patient is lie that like that like that,” because in one ward. And then she feel sad to be there without the family members, because when we send her there she knows there is something wrong with her , and the she will feel sad , and then she will appreciate us coming every day to see her, and of course she also wants to get better, not to be warded, but then we tell her the reason we send you here is because of these reasons, and so then when she starts to stabilize, the doctor certify her to be stable enough to be back home that is what we did, so probably all these thing happens, she notice that she needs to constantly take her medicine to maintain, like what I mentioned the experience that makes her want to continue eating to stabilize. If not she goes back to her previous conditions, and it will cause a lot of trouble to everyone, including herself. Maybe that’s what makes her want to continue eating and the desire to actually get better. When I talk to her she knows she has this conditional and situation. And she keeps on praying that she gets better 2622 and of course we also pray that she gets better, so probably these are the few, these are one of the methods that she knows helps to stabilize her emotions. Yeah.

**<Internals\\SP 140130-0095> - § 3 references coded [3.75% Coverage]**

**Reference 1 - 1.42% Coverage**

What about the severity of their conditions? Do you think that insight might be different or compliance with medication?

Participant: No. Not really, the patients we are talking about are patients that come back voluntarily to come back to see me they would the patients that default completely they are not in this group the patients that just don’t come back until they are very very ill … refuse to see the doctor, not in this group, these are patients who at some level come back to see me repeatedly so while there are various levels inside even within this group most of this people have enough, have the type of insight that makes them come back to see me repeatedly so no I don’t think insight is a significant differentiative between the two, the only thing that came to mind is assertiveness you mention the other thing is type of illness, is that right?

**Reference 2 - 0.77% Coverage**

Now what about the group that does not come back, does not comply and we see them only when they become very ill? Does this group differ in terms of like characteristics compared to those that are willing to other than insight and…

Participant: Well absolutely they tend to beside the fact that they have less insight they tend…their conditions tends to be more severe in general in both… both, I think mostly for the psychiatric condition there is tiny bit of insight

**Reference 3 - 1.55% Coverage**

Do you think there are any barriers to patients getting through the door?

Participant: Many, many barriers, one is cost

Interviewer: Financial…

Participant: Financial cost, inconvenience, lack of insight about these conditions, they may not want to take the things…the polyclinic itself may push back abit if you’re a patient with...”oh, you’re a imh patient” then oh, they want these kind of patients, there is a strong stigma there that they will disturb the other patients some is valid and some not so. you got barriers from the patient point of view, unwillingness to go, lack of insight about their condition, inconvenience, you’ve got system factors, payment so on and so forth, then you’ve got the provider, the polyclinic doctors, GPs who are not particularly… may not be particularly keen to take these patients, “oh you don’t want to come, that’s fine go back and see your imh doctor to give you everything”, you’ve got all three

**<Internals\\SP 140131-0096> - § 2 references coded [2.43% Coverage]**

**Reference 1 - 1.27% Coverage**

so we have quite a few who stop their medication for various reasons , it is not always because of the weight gain, yeah, not only because of the weight gain but insight into the illness, some of them have totally no insight into the illness, so do not think they have an illness, but weight gain is one of the reasons they stop.

**Reference 2 - 1.16% Coverage**

I mean if psychotic disorder insight is always affected to a certain extent, especially during the first few months and episode it takes a while to develop if it develops at all

INTERVIEWER: for insight to develop

PARTICIPANT: for insight to develop, yeah so that affects them taking the medication.

**<Internals\\SP_140109-0130> - § 2 references coded [3.78% Coverage]**

**Reference 1 - 1.89% Coverage**

why they disengage in the first place? Uhm, it is very difficult to say because I usually don’t go in depth and ask why they disengage, a number of them actually came, they have not been seeking treatment for years, that is at least what I know. Yeah, some of the reason they say is I don’t have a problem, I tell them your records show you have diabetes, you were taking medication, it was controlled for a while , and they say no I don’t have a problem

**Reference 2 - 1.88% Coverage**

well we are not talking about age difference, or educational difference, but sort of insight? Do the people who want to follow up have better insight? That sort of is what we have been hearing, right? Do you find that’s the case?

PARTICIPANT: insight? … Insight in what sense, I mean?

INTERVIEWER: insight into their medical illness their psychiatric illness,

PARTICIPANT: yeah that is one of the components but probably not all of them. One of them.

**<Internals\\SP_140123-0084> - § 3 references coded [2.54% Coverage]**

**Reference 1 - 0.40% Coverage**

I guess main challenges I would say is one… a certain proportion of them engaging them in treatment and er that’s related to either not not…having poor insight or the stigma issues ah

**Reference 2 - 0.56% Coverage**

even though we explain that if you’re on this medicine long term, this is going to be a long-term er co-morbidity therefore it needs management er…or just complete lack of insight as in do not acknowledge that…that it is real as big an issue as it is

**Reference 3 - 1.58% Coverage**

I would say…defaulted tend to be those who also do not er have good insight or partial insight or you know limited insight into their psychiatric illness as well so overall it reflects a state of the patient not having taken a great deal of responsibility or ownership in terms of the illness be it psychiatric or medical erm and they are the ones who tend to default or they are the ones who need more supervision from family in terms of taking medication and if family member s have been abit busy then the patient hasn’t gone off to the polyclinic so versus the ones who go to the polyclinic are usually quite insightful take it quite seriously when we explain “ you do need follow-up” and comply with that

**<Internals\\SP_140123-0085> - § 6 references coded [14.10% Coverage]**

**References 1-2 - 6.71% Coverage**

Okay so…for me it’s fortunate for me, because I’ve been rotating to three different departments so far. First is the EPIP – the early psychosis team – so we often see a lot of patients with first episode of acute psychosis, because it’s early psychosis lah. Then I switch to the ward – I was in Ward 34A, so I see those chronic patients and also acute patients lah. And then now I’m in clinic – running Clinic C in most of the community pool patients. Yeah, so to me – it really depends on how good that insight of the patients is with regards to the treatment. Because at the end of the day, it’s their insight and their own willpower that will determine how good and how good their compliance, and how well their illness (02:35). Yeah, so being rotated through these three different postings right, you can see the differences between the acutely ill patients or the early…the first acute episode patients compared to those who are already chronically ill patients. That’s the difference lah.

INTERVIEWER: Specifically which group has more insight, which group has more compliance…? (03:00)

PARTICIPANT: Which group? Erm…so of course the group with more insight, like if they are responding, for instance, for those chronically ill patients, we are talking about chronic psychotic patients like schizophrenia, most of the time they don’t have much insight to their illness. So often we will see a lot of patients, like just a week ago I saw a patient with high blood pressure (03:27), has already been diagnosed with hypertension and hyperlipidaemia but she doesn’t have insight toward the illness despite she’s on regular…the interesting thing is they will still come back (03:43) for regular injection from our side, the psychiatric mental health, but they still don’t have insight to as the other medical conditions. Like every time she comes back for injection, then we will – the routine lah – we will measure the blood pressure. (03:57) Her blood pressure will always be more than 180, they systolic blood pressure. So despite multiple consultations, multiple counselling, that you need to take the medications, because your blood pressure is not well control every time you come back for injections (04:09), despite telling the patients multiple times, and patient just doesn’t have any…insight. Yeah. So she will just show me – it’s a Malay patient – so she will just show me this bible, it’s the Muslim bible, and says that, “Look I’m on traditional medicine. I’m reading this bible, and it’s supposed to help with the illness itself.” But she just doesn’t buy the Western – you know, the treatment and all. (04:38)

**References 3-4 - 3.57% Coverage**

It’s quite common in the common pool patients lah – like the community pool patients, like they will just come back every month for the injections or to top up their medications – to top up their supply. But when you ask about “Why do you think you need to take the medications?” Often a time, they wouldn’t say that they have – it’s for the mental illness – but they would say that “It helps me to sleep better. It helps me to reduce all the disturbances that I’m having.” (05:23). So it’s the symptoms that is making them to take the medications, because the medication helps them to control the symptoms in a way. But then I guess for more for hypertension, and hyperlipidaemia and all these illness right – the chronic illness – because there isn’t like any life-destructing symptoms per se? So they don’t see the point of taking the medications like, high blood pressure they are asymptomatic – they have no headaches, they have no blurred vision anything right, so…to them, like look unwell, like don’t have any symptoms, “why do I have to take the extra pills?” That’s what I gather the feedback from most of the patients lah. And that’s the (06:07) reason I guess why, you know, they come back to take medication and injections from the mental health side, but not the chronic illness. Because to them, they are – you know the reading is high, but so what? I’m still well and asymptomatic.

**Reference 5 - 1.50% Coverage**

I mean their illness and the role of the physician plays both – an important role, because if the patient doesn’t have any insight, no matter how good we psychoeducate the patient, there’s a chance that the patient just don’t buy your explanations lah. And they will keep defaulting the follow-up. And for those patients who have good insight, they are able to understand that after multiple attempts of like explanations and psychoeducation and counselling, they will eventually understand more about their illness, the consequences, and they will start taking medications more regularly.

**Reference 6 - 2.32% Coverage**

Because if they don’t have insight, then no matter how much counselling you give them, will still not buy it. But then it’s also important to involve the family members also. Because if let’s say the patient has no insight to their illness, at least we can get the family member to monitor for them, at least to supervise their medicine. Because like most of the time the patient like come back to us and then they have this uncontrolled BP per se, then if after much explaining/explanations to the patients they still don’t’ go for the appointment then I will just ring up the family members. So involving the family member is also another way to help the patients to monitor their conditions. At least somebody there for them to help them with the medications, at least supervise them and remind them to take the medications. So involving the care of the families also matter if we can’t engage our patients.

**<Internals\\SP_140125-0087> - § 1 reference coded [1.56% Coverage]**

**Reference 1 - 1.56% Coverage**

I guess to some extent if we do have patients who are cognitively impaired, like for example chronic schizophrenic patients, sometimes when they see other physicians they may have difficulty expressing themselves, and so sometimes their complaints are less , not taken as seriously, like non psychiatric patients. 1121 other barriers… I guess financial barriers, yeah like I was saying the medifund is not translated.

**<Internals\\SP_140202-0098> - § 1 reference coded [1.00% Coverage]**

**Reference 1 - 1.00% Coverage**

I will be in the best position to help this patient, yah psychiatry is a very difficult process to get across to patient sometimes many of them do not have insight into their medical conditions so I mean they probably won’t have insight into psychiatric conditions sometimes there are people no matter how many years of treating their psychiatric illness they may not develop the insight that they have an illness

**<Internals\\SP_140215-0120> - § 1 reference coded [0.51% Coverage]**

**Reference 1 - 0.51% Coverage**

I mean outpatients if kind of they are in a state of relapse, then that may sometimes colour the picture that may influence I think their insight. So their willingness to go back

**<Internals\\SP_151210-0132> - § 2 references coded [2.64% Coverage]**

**Reference 1 - 0.98% Coverage**

if we have a good service that captures people in on their first episode, and you have a shortened duration of untreated illness, the evidence suggests that these patients have better insight, they respond better to treatment they stay longer in remission and they are able to manage themselves.

**Reference 2 - 1.66% Coverage**

I have seen I have visited people who have non-psychiatric disorders, but they are from lower socioeconomic income families, and honestly the lack of insight into their conditions, and they would have two or three, hypertension poorly controlled heart failure, maybe diabetes, their lack of understanding with what is going on with their bodies and how they should be taking their medicines and how that should be coupled with a change in their life style is no better than a person with schizophrenia.
