# Supplementary material for: The Impact of Stakeholder Preferences on Service User Adherence to Treatments for Schizophrenia and Metabolic Comorbidities
Source: PLoS One. 2016 Nov 16;11(11):e0166171. doi: 10.1371/journal.pone.0166171 (PMC5112999; doi:10.1371/journal.pone.0166171)
Supplement: S1 File — This file contains the nodes used to construct the themes reported in the manuscript. Including advice to others; expertise; insight into illness; instructions; looking after kin; preferences; relapse; resistance to doctor’s orders; social factors; social support; stigma; therapeutic alliance; and uneasy about initiating treatment. (ZIP) [file pone.0166171.s001.zip › Qualitative data/Instructions.docx]

**Name:** Instructions

**<Internals\\HDL interview 2 20151111171452547 no audio> - § 1 reference coded [4.10% Coverage]**

**Reference 1 - 4.10% Coverage**

Worries of relapse if he does not follow up the clozapine, so is very careful about following up with his visits. And was told he could come any day in emergency.

**<Internals\\HDL study -Service user HDL_140211-0114> - § 1 reference coded [0.80% Coverage]**

**Reference 1 - 0.80% Coverage**

and they had your physicians, your psychiatrist, have they change your medication?

PARTICIPANT: no, in terms of quantity yes.

INTERVIEWER: yes.

PARTICIPANT: in terms of duration yes, the types specifically no.

INTERVIEWER: ok

PARTICIPANT: I think they actually had kind of targeted at my condition exactly. And I guess this proved to be effective. So, I just go along with it.

**<Internals\\HDL Study_service user HDL_151218-0133> - § 2 references coded [3.32% Coverage]**

**Reference 1 - 1.77% Coverage**

INTERVIEWER: and was the doctor doing regular tests?

PARTICIPANT: yes

INTERVIEWER: so regular blood tests

PARTICIPANT: yes regular blood tests. I think I saw the doctor two or three times, two or three times, after that I understood the doctor’s advice, advice meaning “don’t take more sugar, don’t take sweet food, don’t eat foods which are sweet”

**Reference 2 - 1.54% Coverage**

INTERVIEWER: did you take any medication for the high blood pressure?

PARTICIPANT: yes and then the doctor said to avoid sour stuff,

INTERVIEWER: sour?

PARTICIPANT: like sweet and sour stuff yes. Sour food and drinks. I understand the doctor advice, and this [incomprehensible] my high blood pressure.

**<Internals\\HDL study-service user HDL_151209-0152> - § 2 references coded [0.97% Coverage]**

**Reference 1 - 0.43% Coverage**

And then my dr say got to, refer me to, refer la. Because the polyclinic general medicine. General medicine they.. they see they say ah… they see ah, high cholesterol problem issue. Ah

**Reference 2 - 0.54% Coverage**

got people say come to IMH, the medicine cannot change. Is true ah. The medicine they give cannot change. I ask Dr, say can you change my ah.. can you change my medication? He say cannot change. Wait I scared you… you relapse ah.

**<Internals\\HDL Study-Service User_140113-0128> - § 1 reference coded [2.13% Coverage]**

**Reference 1 - 2.13% Coverage**

and so, when you found that you had the elevated lipids… did the psychiatrist prescribed anything for your medication? Or prescribe anything for that? How did..

PARTICIPANT: the psychiatrist didn’t prescribe anything. But then he has actually asked me to go to the polyclinic to get my medication. Yah.

INTERVIEWER: so. And then you went to the polyclinic?

PARTICIPANT: yes

INTERVIEWER: and how did that work? What did they do at the polyclinic?

PARTICIPANT: they just review through the blood test results that was done in IMH. And then told me that I actually have to take this pill.
