# Supplementary material for: The Impact of Stakeholder Preferences on Service User Adherence to Treatments for Schizophrenia and Metabolic Comorbidities
Source: PLoS One. 2016 Nov 16;11(11):e0166171. doi: 10.1371/journal.pone.0166171 (PMC5112999; doi:10.1371/journal.pone.0166171)
Supplement: S1 File — This file contains the nodes used to construct the themes reported in the manuscript. Including advice to others; expertise; insight into illness; instructions; looking after kin; preferences; relapse; resistance to doctor’s orders; social factors; social support; stigma; therapeutic alliance; and uneasy about initiating treatment. (ZIP) [file pone.0166171.s001.zip › Qualitative data/Looking after kin.docx]

**Name:** looking after kin

**<Internals\\HDL_CG 151023_0036> - § 3 references coded [10.54% Coverage]**

**Reference 1 - 2.81% Coverage**

PARTICIPANT: yeah, we, uhm, this , at times I’ll ask him if he hears any voices, because it is my concern , I need to know because if anything happen I hope he will seek doctor help as fast as possible, but I don’t know if these are sensitive questions for him, at times he will hide from me. And he doesn’t want me to know. Maybe makes a face, I don’t know. I told him before that “I don’t mind to share this burden with you, but you need to take care of yourself” that’s my story.

**Reference 2 - 3.52% Coverage**

uhm not yet because I haven’t seen, because at ties I come with him and I realize that if I question the doctor, he will be quite sensitive and tense, like “why are you asking the doctor so many questions?” so at times I only ask questions that are important, because I am afraid he is a bit too sensitive, like him getting angry over me asking too many questions. Then uhm that is why I was saying that he is not aware of his condition, he should actually ask the doctoral, if he is well he will know that this problem, he should ask the doctor what can he do about it, why is this the medication? Yeah

**Reference 3 - 4.21% Coverage**

yeah make him understand and how is he going to like , uhm/.. Like make him... understand deeper, and how to let him help himself, alter himself to try and reduce the medication and be back to normal again.

INTERVIEWER: so in that sense, have you tried to help him?

PARTICIPANT: i… because the only think I can help him is , I give him advice, like be happy, be content for what you have now, you have nothing to stress about, no need to feed the house, no need to pay rent, just earning money to support your own self , and you can buy whatever you want. The only thing I tell him is I am making his room more green, yeah. Other than that I think there is nothing else I can do, the rest he has to work on himself. Yeah.

**<Internals\\HDL_CG 20151105 notes, no audio> - § 1 reference coded [16.09% Coverage]**

**Reference 1 - 16.09% Coverage**

Been carer for 10 years in the same family, spends 4 hours a week with him taking him to appointments, but he is able ot go to IMH and polyclinic on his own, however, visits on his own tend to result in changes to his medication and diagnosis which confuses the care givers, they wish they could get a memo explaining the changes and rationale.

**<Internals\\HDL_CG151209-0142> - § 4 references coded [8.69% Coverage]**

**Reference 1 - 0.96% Coverage**

PARTICIPANT: this month, she did not go because she said she did not want to go. So I have no choice to not take her. So I go…

P1: she had the blood test but two days , one week later, to see the result she refused to go…

PARTICIPANT: so I went without her, and they said no, at the polyclinic they cannot, they need the patient to be present, otherwise cannot, so I leave it.

**Reference 2 - 3.77% Coverage**

, people know about things there, so I was[incomprehensible14.44] it was 2 3 or serious, and said ok she can go to school but in the end, to the end I follow up and see, one day one of the doctors consultants from IMH took trip to there, and he gave a talk at orchard hotel about the medicine risperidone

INTERVIEWER: yes risperidone

PARTICIPANT: and he say the presentation, he is called doctor [name] he said, no you just take the medicine it would be very easy, I hear the doctor. After that I bring her to NUH, to follow for I don’t know how many years, after that , before that we were in the [hospital] they do ECT I don’t know how many times 4 quite a number of times, also the symptoms also like this, not help much, so I bring her, I went to the orchard hotel to listen to the talk about the risperidone new drugs, so I told the doctor at nuh to continue until I don’t know how many years, and then her take risperidone for how many years, and here [above the chest] has swollen a lot of water coming out, so maybe because of the medication

INTERVIEWER: medication

PARTICIPANT: and then the medication also not effect already la, it lost effect after that, maybe not , maybe the time the doctor asked me, she said the [incomprehensible] so I asked the doctor because she looked tired whether the medicine can reduce the dose, the doctor asked to observe her I said can, at the time I did not know much [incomprehensible] the doctor said yeah he can review one the mg

**Reference 3 - 3.03% Coverage**

INTERVIEWER: ok another thin you mentioned was that sometimes when you had the blood tests done here at IMH if it was for the liver function test , you would ask them also to do the cholesterol test, since you were drawing blood, why do you prefer it done that way?

PARTICIPANT: because I want to get here to see the doctor is not easy, depending on mood

INTERVIEWER: so once again it is challenge of getting her to come and go

PARTICIPANT2: this is more the patient side

INTERVIEWER: yeas ok

PARTICIPANT: and then I even bring her to the police AnE, if she come here, or she don’t want to calm down , I ask police to help me, sometimes she will come after two weeks come, she don’t want to come for injection

PARTICIPANT2: she will say “you go for injection”

PARTICIPANT: I will ask for help, in the middle of the night I will ask her to come help me bring together to AE, very hard for the caregiver when the caregiver is me, I am not young, I am 60 years old, I also got angioplasty, so I am tired, I need help, that is why, I don’t have the strength, it is very , she make up her mind to don’t come, she don’t come.

PARTICIPANT2: strong willed

PARTICIPANT: strong willed yes

**Reference 4 - 0.93% Coverage**

very taxing, some will [incomprehensible] cannot take the stress, so we are taking care of my daughter so many years 14 years, and some people encourage send her to hospital for treatment, and I say “no I cannot listen to you” because the symptoms are there, they cannot manage, then they listen to us, they also learn from this sharing sharing, very stressed.

**<Internals\\HDL_CG151209-0158> - § 4 references coded [10.46% Coverage]**

**Reference 1 - 4.97% Coverage**

my sister told me she found out he was naked dancing in the room. So I caught him, and returned him here, so they found out there is some disturbance then, that was 1 year ago I think. And now, every month , taking for medication injection, so since he taking his injection, I found out there is a big difference, no disturbing I mean, he can, he can be advised, “don’t do this, don’t do that” and ever since, he followed. He follow. Before the injection, he can take your advice, but won’t do. Never come back for 2 3 days, so since taking the injection I find a big difference, he can listen to me, even thought, we are not staying together, I have to find the time to rush and look for him for a few hours and then rush to come back again. My sister also not so free. Can take a few minutes to look, different Unit, one Block different unit, same building, and the maid, also have to look for him, but the maid also go around and try to leave him alone. So my parents stay with my sister at present. So he is staying along at present in a room , they look around him, they also look for him, to see if he makes any problem, so far no. since his admission to here [IMH] I think they recorded, I don’t want you so far, ok

**Reference 2 - 2.17% Coverage**

INTERVIEWER: so when he comes for the injection, does he come alone sometimes?

PARTICIPANT: I try to bring him, alone cannot, paranoid, he doesn’t know what to do, so he just…like... tomorrow I bring him, try to let him, he cannot, he has to be guided, to bring him, I try my best to bring him, get him to go alone? No, I think he cannot.

INTERVIEWER: cannot. Do you have strategies to help him overcome those difficulties so you can bring him without the difficulties?

PARTICIPANT: no I have to go with him, I don’t have.

**Reference 3 - 1.35% Coverage**

PARTICIPANT: I do, I had to build it, my brother alone!, older, yeah, but younger, only one, like my sister cannot take care of him, taking care of my mother, bed-ridden, so I think I try my best, have the free time, have to rush and look, and take a look, even if it takes 2 hours three hours, have to take a look, then come back

**Reference 4 - 1.97% Coverage**

it is so stress, but I have to take it, because compared to before, it was more, but now it is ok, I feel like I can check with my sister, and they didn’t complain about it, or the maid, she see the medication, regularly take. But medication is very important to me, have to check, if you can ask him to take it in front [of you] he is very obedient, because I told him it is important, he knows, but, he knows what is the situation la, he is very obedient, to me he is very obedient.

**<Internals\\HDL_CG151209-0159> - § 6 references coded [22.08% Coverage]**

**Reference 1 - 3.47% Coverage**

it’s a burden, it’s a burden. Taking the responsibility, because we can give them medicine,, because what they can do, is we can give them medicine medication …and if they take the tablet or not is also, I can just put the tablet there, and it is up to him to take it, I can’t force him to take it, he should understand, it is ok he would just take the tablet, if not, I can’t do anything.

INTERVIEWER: so are there times when he refuses?

PARTICIPANT: I just put the tablets there it is up to him, his responsibility and if he wants to get well then he takes, but when he takes tablets, you have to eat , you know, you have to take your meals … regularly, so if you don’t take you will feel a bit weak, because his appetite is quite bad. And smokes! He smokes terribly!

**Reference 2 - 3.93% Coverage**

INTERVIEWER: so do you think that you have developed any particular skills from managing your siblings?

PARTICIPANT: skills? You need a lot of patience, but whatever skills, I don’t think it is skills?

INTERVIEWER: what is it then the patience?

PARTICIPANT: well I think it is patience and long suffering what you call it.

INTERVIEWER: long suffering?

PARTICIPANT: yeah it is like a like what you call it…all the while you know there is this these sick people around and you have to take care it is sort of a burden.

INTERVIEWER: can you tell me a bit more about that?

PARTICIPANT: same things I said I have to repeat.

INTERVIEWER: sorry

PARTICIPANT: currently the place the house is for them, I cannot go travel leave them alone or go out , I can’t do that. They are not they are irresponsible I have to say that they are, and I think that is all I can tell you.

**Reference 3 - 2.82% Coverage**

one of the things we are curious about is how you sort of became , how you came into the role of having to care for them. So

PARTICIPANT: I have no choice because there are 5 of us and I am the eldest! The youngest who passed away, he was the one who also helps, helps me to take care of them because we are living together. Yeah so sometimes he brings them down to the clinic, when he can, so that I don’t have to bring them down…

INTERVIEWER: so while you were working as an air stewardess, he was responsible?

PARTICIPANT: we both shared I mean shared, if he is available he brings them down, if not I will bring them down.

**Reference 4 - 3.08% Coverage**

INTERVIEWER: one of the things we ask, it may not apply exactly to you, but how has your life changed since you have taken on the role of care giver, if you have impressions

PARTICIPANT: it is stressful and frustrating , because I have to work, and also think of the home! And the, it is not an easy task.

INTERVIEWER: so you have those duel responsibilities of looking after them and looking after the home?

PARTICIPANT: it’s not easy!

INTERVIEWER: you mentioned frustration, do you mind if I ask you a bit more about what what specific things are frustrating?

PARTICIPANT: my goodness! Frustration can, there are so many categories! So just say a word “disgusted with everything”

**Reference 5 - 5.93% Coverage**

PARTICIPANT: they are already coming for treatment, like this there is already an appointment to meet the doctor, “can you hear voices ?” or… the same questions they will repeat. So it is fine, because a few minutes with them , but we caregivers, we are the whole day with them! You know that’s a different story.

INTERVIEWER: can you tell me a bit more about that?

PARTICIPANT: yeah, I mean each day is a different thing, like we won’t know what to …what they call it, unforeseen circumstances just pop up, each day is different.

INTERVIEWER: you have to understand that I have never been in this role, so we are trying to understand what it is like to be you. I think you are the 7th person I have spoken to who is in this role, we hope to speak to another 5 or 6. What do you mean when you say that there are unforeseen, or that there are still changes?

PARTICIPANT: yeah you see all of a sudden my brother passed away, it is quite sudden. So that will be a lot of changes within the family, yes, and if I pass, then who is going to look after them, that is another big headache, so they have to understand , they have to be placed in the home or someone , who is going to take responsibility to manage them or look after the place? This kind of thing, so this is also a worry. But I don’t think they understand this.

**Reference 6 - 2.86% Coverage**

PARTICIPANT: yeah, I think they don’t know they think we are forcing them or ,,, I mean this thing, so it is not easy. Who is going to take responsibility? Parents are not here, so nobody!

INTERVIEWER: and how do they express that worry?

PARTICIPANT: they don’t express anything because they don’t understand anything , they will just listen and say yes

INTERVIEWER: uh hum, do you think that there is anything else that can help us improve services? Or any type of service that you think we should offer, but that we are not offering?

PARTICIPANT: hum…. I guess that is the best you all have offered, so it is just wait and see?...

**<Internals\\HDL_CG151210-0163> - § 4 references coded [17.07% Coverage]**

**Reference 1 - 5.17% Coverage**

before this when I lived with her of course I tried to help her in everything like house chores cooking, maybe just peal the onions, the simple stuff, but as for the main cooking she does it. All the major movement of furniture and all that of course it is the children that does it. And then going out for appointments, going out, bringing her to one point or another. If, let’s say I am occupied, either my brother or sister would accompany her, if not she would usually go with my father, but he himself is also facing some, he also has diabetes and all this, and he also walks quite slowly, he needs a walking stick to walk, so uhm …if all of the children can’t make it, usually the two of them will go out, but at their own pace, a lot of stopping, a lot of sitting a lot of resting and all that. And of course on short distances , not going out to shopping malls and all that, but just to hospitals and maybe have a quick lunch and then come back home. Those are the movement that she does, usually we also advise her to do some exercises at home, or we offer ourselves to bring them downstairs to walk , because we know her weight is one of the main factors, and even when we come for appointment , they keep emphasizing that she has to reduce her weight, but looking at her condition, I know she will not be able to do what we do, which is, if we know we are overweight we can start jogging brisk walking, but for her it is quite hard, because on normal days when she walks so slow, she can only walk maybe 5 ten minutes then she needs to rest, but I know the nurses and the doctors propose to so strengthening at home, do the cycling, we bought all that, but of course the other thing is the will power , so for me, her will power to really exercise, is not that great [laughs] so she will just resort to just waking up, do her normal routine resting watch TV and stuff like that, but for exercising portion ,I think her will power to do it is not that high to actually, you know, push all the tiredness away and do it for 30 minutes, I think, at least at this moment she is not strong like that.

**Reference 2 - 2.31% Coverage**

is it simply to bring her, or is it because she needs someone there?

PARTICIPANT: for now she is not so stable in terms of her posture, so she needs someone to hold her, and then the other thing is, usually she wants us to be around because sometimes when they speak in English she may not understand so we translate for her, or we share, or certain things she has difficulty conveying to the doctors or the nurses, and then the children are the ones that relay information,. And then I understand that during this kind of appointment, the children can share what we see at home to the doctors, the doctors meet once every month or every 5 weeks, but we are the eyes to see what is happening at home, whether she is stable or whether her moods are going up and down, and personally for my mom, aside from all that, I think it is a bonding time, that she wants the children to bring her to hospital, yeah, so, yeah I see it from that angle.

**Reference 3 - 2.70% Coverage**

PARTICIPANT: I think firstly of course, the burden the first one is to actually bring her to be warded at IMH, of course if we say we need to bring you to IMH she wouldn’t want to, she would not willingly come, so we need to say that “oh let’s go out to eat” and then suddenly we will drive the car, and then she will ask “hey why are you bringing me here?” and then try to coax her, tell her that oh it is only for a normal check-up and then she realize that she is to be warded. So first is of course the pain to separate yourself from your parents or your mom and know that she will be warded for one week two weeks, and then next of course is the burden to come and visit her every day, you know the whole mood of the family, but you know your mom’s mood is at its lowest when she display this kind of these kinds of emotions, so it is more the emotional part, and physical in terms of travelling to see her. But ultimately we all know that we are doing this for her own good so that she can recover and stabilize herself. That is the burden I am talking about, more the emotional aspect of it…

**Reference 4 - 6.89% Coverage**

PARTICIPANT: I believe all caregivers have their own different good bad, their own different experiences. So for me firstly of course it take a high level of patience, and then if she is not able to do certain tasks, we cannot, we need to sympathize with her, we cannot see her like how we see ourselves, meaning being able to walk for long distances, you know and all that [can’t expect her to do the things we can do] so firstly I believe knowing about ,as a caregiver, knowing about the person you are taking care of, the conditions of the person is very important, so you can actually understand why that person can or cannot do certain tasks and then we need to always remind ourselves to be patient, patience towards that person. … and of course work together, because for me personally, I am working, I also have my own family, and then now I am living in woodlands, so I appreciate both my siblings, we take turns or whoever is free on that day accompany, or if all of us are working and she needs accompany, and my dad can’t accompany her, one of us has to take leave, you know to take care of her, or to bring her. So I would appreciate the family support or the community support in this as well, because , you know having a family member that is having this conditions, it is challenging in its own way, like for example, I believe, let’s say you have a family member who has this experience, for me I visit your place, I see your grandmother is having this schizoaffective disorder or schizophrenia, and then I would sympathize and give my 120% attention to her for that day, but then I see how you acted toward her differently, like you want to get things done quickly or easily frustrated, for me I understand that situation already because I experienced it one time off, but for you it is a daily affaire, and you have been helping her , for example if she asks for a coffee and you make her a coffee, and then she said “I don’t want coffee I want another drink” you know , and it is not a one-time affaire, it has been ongoing for a lot of time, so you as a caregiver you accumulate all this stress all this frustration and you might you MIGHT, or that caregiver may blurt it out or blast it toward the person, to the patient. So that family support and understanding from the community is really important I feel. Because sometimes when I bring my mother out and then the way she walks, she takes time entering the lift, and then a lot of the people at the back, are upset, making those types of remarks … you know… I know her, she is walking like this because of her condition, but you need to be more patient for the public I mean. So yeah those are the things, patience understanding about the person’s health condition, and the support from family and the community, it really helps.
