# Supplementary material for: The Impact of Stakeholder Preferences on Service User Adherence to Treatments for Schizophrenia and Metabolic Comorbidities
Source: PLoS One. 2016 Nov 16;11(11):e0166171. doi: 10.1371/journal.pone.0166171 (PMC5112999; doi:10.1371/journal.pone.0166171)
Supplement: S1 File — This file contains the nodes used to construct the themes reported in the manuscript. Including advice to others; expertise; insight into illness; instructions; looking after kin; preferences; relapse; resistance to doctor’s orders; social factors; social support; stigma; therapeutic alliance; and uneasy about initiating treatment. (ZIP) [file pone.0166171.s001.zip › Qualitative data/Preferences.docx]

**Name:** Preferences

**<Internals\\HDL interview 1 20151009144255082 no audio> - § 2 references coded [21.05% Coverage]**

**Reference 1 - 4.26% Coverage**

Likes the division between IMH and polyclinic as each setting is suited to treat each condition, believes that the two are best source.

**Reference 2 - 16.79% Coverage**

Satisfied with the treatment she is currently receiving from both the polyclinic and IMH. Feels that she has no major problems in receiving treatment in the various settings; does not mind the long wait at the polyclinic (“smooth sailing”). Thinks it is better to get treatment for Schizophrenia at IMH and treatment for chronic conditions at the polyclinic as they are both skilled at treating the respective conditions; feels that if she comes to imh for her diabetes, they might not be able to understand her condition vice versa.

**<Internals\\HDL interview 2 20151111171452547 no audio> - § 3 references coded [13.94% Coverage]**

**References 1-2 - 9.34% Coverage**

Does see the convenience of having treatment all in one spot, but prefers to see as he does, polyclinic and psychiatrist as the high blood pressure is routine and common and can be handled by polyclinic, but psychiatric conditions requires more experience “ when you have a condition like this, you prefer to be treated at a place that has treated people for quite some time”

**Reference 3 - 4.60% Coverage**

Also pleased about the doctors at IMH who have been respectful and been able to produce rapport with him. They know how to handle him here. Prefers IMH for treatment of mental illness.

**<Internals\\HDL interview 3 20160217171017621 no audio> - § 1 reference coded [17.13% Coverage]**

**Reference 1 - 17.13% Coverage**

Psychiatrists never referred her to polyclinic for the treatment of her cholesterol, unclear why. But if the psychiatrist had told her, she would go. Would go to polyclinic for both treatment of psychosis and cholesterol but has “no choice” but to come to IMH . doubted that polyclinic would have the right medication to give out.

Would prefer to be in polyclinic near her house, but no choice since they do not have the same medication.

**<Internals\\HDL Study - service user HDL_151224-0139> - § 5 references coded [12.57% Coverage]**

**Reference 1 - 5.50% Coverage**

INTERVIEWER: is that ok? Do you prefer going to the polyclinic for treatment?

PARTICIPANT: yes

INTERVIEWER: yah?

PARTICIPANT: yah, I prefer to go to the polyclinic for treatment

INTERVIEWER: why?

PARTICIPANT: ah.. its much more convenient. Convenient to my home. Nearest.

INTERVIEWER: ok

PARTICIPANT: yah

INTERVIEWER: so, its easier for you to go to the polyclinic

PARTICIPANT: yes.

INTERVIEWER: to get treatment for your diabetes and high cholesterol

PARTICIPANT: yes.

INTERVIEWER: would you prefer if they could also give you the treatment for schizophrenia?

PARTICIPANT: in the polyclinic? No, they don’t give me for schizophrenia.

INTERVIEWER: no they don’t

PARTICIPANT: only the IMH.

INTERVIEWER: but would you prefer? Do you like it? If the polyclinic could give you the treatment for schizophrenia and you don’t have to come down to IMH?

PARTICIPANT: they give me to go to the this place. Admiralty ?? (14:15). To get my medication.

INTERVIEWER: ok.

PARTICIPANT: for upcoming treatment.

INTERVIEWER: ok. So, that’s.. is that close to your house?

PARTICIPANT: yes

INTERVIEWER: yes. So, from now you can go get treatment there

PARTICIPANT: yes yes.

INTERVIEWER: and you prefer that?

PARTICIPANT: yes.

INTERVIEWER: ah..

PARTICIPANT: I don’t need to come here and take medication anymore.

INTERVIEWER: ok

PARTICIPANT: yes.

INTERVIEWER: and you prefer that?

PARTICIPANT: yes.

INTERVIEWER: yes. Only because it’s closer?

PARTICIPANT: yes. Closer to my home.

**References 2-3 - 3.47% Coverage**

INTERVIEWER: is that ok for you?

PARTICIPANT: yes

INTERVIEWER: to go to 2 different places?

PARTICIPANT: yes

INTERVIEWER: or would you prefer to have 1 DR treat everything?

PARTICIPANT: ah.. I don’t know whether they have a new diabetes treatment here. Because they din tell me that, maybe I go to 2 place at 1 time.

INTERVIEWER: and that’s ok?

PARTICIPANT: yes.

INTERVIEWER: why do you not.. why would you prefer there over IMH?

PARTICIPANT: because it is convenient and its cheaper.

INTERVIEWER: cheaper?

PARTICIPANT: yes. I show them my blue card. This card and I can get the discount.

INTERVIEWER: ah…

PARTICIPANT: $16 only.

INTERVIEWER: so its cheaper

PARTICIPANT: they ask around.. I can get $14 for that. Yah. Discount.

INTERVIEWER: ok.

PARTICIPANT: ya

INTERVIEWER: so makes it cheaper for you to go there.

PARTICIPANT: yes.

INTERVIEWER: do you know why its cheaper there than here?

PARTICIPANT: ah,.. I don’t know.

**Reference 4 - 0.98% Coverage**

INTERVIEWER: do you think polyclinic is better for treating diabetes than IMH? If IMH could get treatment for diabetes here. Would you prefer here or?

PARTICIPANT: ahh… I so I can prefer here, but they always… they ohh ah.. located me here already. So I have to go here.

**Reference 5 - 2.61% Coverage**

INTERVIEWER: Ok. And.. you don’t mind seeing the polyclinic and the psychiatrist.

PARTICIPANT: yes

INTERVIEWER: ok. And you wouldn’t prefer if you only had to see 1 DR.

PARTICIPANT: no

INTERVIEWER: no

PARTICIPANT: I can see 2 Dr at in one time. At one time.

INTERVIEWER: in one spot? Or? I don’t understand.

PARTICIPANT: I don’t mind visiting DR, 2 Drs in one time. I don’t mind that.

INTERVIEWER: In one time. What do you mean?

PARTICIPANT: I can visit 2 DRs at one time. Psychiatrist and diabetes DR.

INTERVIEWER: but on the same day? Or at the same place? At the same clinic

PARTICIPANT: different time, different day

INTERVIEWER: different time, different day

PARTICIPANT: I… different clinic.

**<Internals\\HDL study - service user HDL_151210-0153 (chinese)> - § 3 references coded [6.63% Coverage]**

**Reference 1 - 0.77% Coverage**

: ok. 除了等了很久、除了好象。。 因为polyclinic 等很久、 你还有什么好象会比较喜欢这里拿药吗？ (ok. Besides wait very long… beside waiting.. because polyclinic wait very long. Anything else that you prefer taking medications here?)

P: 没有 (no)

**Reference 2 - 2.38% Coverage**

ok、 所以你在这里有拿药。 对不对？ 在polyclinic 也是有拿要过。 那你觉得这两边你如果你有一个选择、 你会想在哪里拿药？(ok, and you take medication here. right? At polyclinic also have taken before. Then you feel 2 sides, if you have a choice, where do you prefer to take medication?)

P: 这里咯。(here)

I: 这里。 可能跟我讲多一点吗？为什么呢？(here. Can tell me more? Why?)

P: 这里靠近我家咯。 (here closer to my house)

I: 靠近你家啊。 Ok. 还有吗？(close to your house. Ok. Anything else?)

P: 在这里、 我比较。。 我比较熟咯。(here.. I in comparision…. I’m more familiar)

I: 这里比较熟。 但是你在polyclinic 也是拿了很久了吗。所以你对那边。。。(familiar.. but at polyclinic you also take very long already. So you also at that side..)

P: 不久 啦、 不久。 (not long, not long)

**Reference 3 - 3.49% Coverage**

I: 每次换来换去啊？那你对他们有什么看法？ 你觉的有ok 吗？还是有什么东西你觉的不怎么好。 (always change? Then what do you think about them? Any thoughts? What do you think is not good?)

P: 现在好象是换新的医生、旧的医生没有看了。 (now is like new dr. previous dr not seeing anymore)

I: 那什么样？ 你觉的。。 觉的他ok 。。。 (so how? You feel he is ok?)

P: ok la.

I: ok. 跟。。 他跟旧的医生有什么分别？(he and the previous dr, any difference)

P: 旧的医生、 跟他讲话、 他比较会咯。 新的医生就好象不大会这样。 (previous dr, tell him, he knows better. New dr , like not like that)

I: 比如呢? (for example?)

P: 新的医生、 我要跟他讲、 跟他讲。 旧的医生他问话、 我们就。。 他就知道我们什么病。 (new dr, I tell him, tell him. Previous dr, he ask me. We can… he knows what condition we have)

I: ok、 你问他什么、还是 他问你什么东西？ (ok. You ask him what, or he ask you what)

P:他跟我们讲的东西、 then 就拿药了。 新的医生跟他讲、 还要讲咯。 讲我什么病。 他不知道。 新的医生不知道。旧的跟他讲、 他就知道咯。 (he tell us things, then take medicine, new dr need to tell him, tell him. Tell him my illness. He don’t know. New dr don’t know. Previous dr tell him, he know already)

**<Internals\\HDL study - service user HDL_151222-0138> - § 3 references coded [6.47% Coverage]**

**Reference 1 - 1.74% Coverage**

INTERVIEWER: but what paper work do you have to see the GP for?

PARTICIPANT: for example, got no money, apply for medifund. For financial assistant. Something like that la.

INTERVIEWER: So because of that, you prefer seeing Dr Jimmy? Or because you don’t want to see GP? Or you can’t see a GP?

PARTICIPANT: because money problem la. Cannot Cannot go and see a psychiatrist. Money problem la, cannot. Cannot, cannot. Sir, I’m ok already. Ok already. I still feel i feel fine after taking the small, the 3 small pill.

**Reference 2 - 2.21% Coverage**

INTERVIEWER: yah. And how did you like receiving treatment at that place? Was it ok?

PARTICIPANT: at first I don’t feel anything. But now, I don’t know, I don’t know. I think that it is some kind of changes in my life. When I was diagnosed schizophrenia, that time were to ask me do I hear anything or not? I say hear hear, hear, what thing? Hear people quarrelling lo, arguing all this and that. Noises. Where got see. All this efforts hor, I keep on saying medicine won’t help me. Help me. But now, it seems that everything is ok for me already. My father say Dr got many years study, so they are.. they are.. what they, their treatment is perfectly correct.

**Reference 3 - 2.52% Coverage**

PARTICIPANT: I think because the Dr, I think at one time some people, the Dr ask whether I want to get 3 months medicine or not, instead of every month come and work. Come and see Dr. I say cannot la. Like that, I tend to, after I forget everything. That’s what I don’t dare to try it. The Dr arrange us, ask me want to go for 3 months medicine. No no every time come, like that ah. But I said I cannot accept it. I scared the feeling come, maybe within 3 months I got relapse already. I cannot try. I cannot take my chances. So now I stick, stick to 1 month lo. Now.. clozapine like that, 4 weeks one time.

INTERVIEWER: so you prefer coming more often?

PARTICIPANT: yah. You know why? I want to keep check whether my mind is playing tricks on me or not.

**<Internals\\HDL study -Service user HDL_140211-0114> - § 3 references coded [3.41% Coverage]**

**Reference 1 - 0.80% Coverage**

and they had your physicians, your psychiatrist, have they change your medication?

PARTICIPANT: no, in terms of quantity yes.

INTERVIEWER: yes.

PARTICIPANT: in terms of duration yes, the types specifically no.

INTERVIEWER: ok

PARTICIPANT: I think they actually had kind of targeted at my condition exactly. And I guess this proved to be effective. So, I just go along with it.

**Reference 2 - 1.99% Coverage**

INTERVIEWER: and how do you feel about getting treatment for your mental illness here at IMH? And then for your high blood pressure or cholesterol at the polyclinic?

PARTICIPANT: no choice is the only way I can say it. If given a preference I wouldn’t want to. Guess whenever I see a DR, I always bring up the subject of reducing or stopping. You know? But they explained the reason of it so I’m ok with that. And in terms of the blood pressure, also.. because this is chronic. I suppose chronic is for life term you know. But they did mentioned ok we can consider it if you bring your weight down. Because it got linked to it. So I’m trying to do that. So bring my weight down, so my blood pressure is actually stable. So is showing progress but is very slow. I mean from poly they can see, they weight, take the blood pressure is stable, weight is going down. But I have to give it some time lah. It cannot be immediate as how I would like it to be.

**Reference 3 - 0.62% Coverage**

PARTICIPANT: if possible, but I don’t think really can like cater for all you know? Because of the.. I suppose.. bedok is actually nearer to me. I mean in terms of convenient. It’s just.. compared to here. But I don’t know whether bedok poly can really prescribe the medication which you are giving

**<Internals\\HDL Study Service User HDL_151209-0145> - § 5 references coded [13.66% Coverage]**

**Reference 1 - 5.02% Coverage**

INTERVIEWER: how well do you think that works for you? Is it ok?

PARTICIPANT: yah yah. Its alright.

INTERVIEWER: yah?

PARTICIPANT: yah

INTERVIEWER: because we find sometimes it’s that some people don’t like going to places for different treatments. And what happens is then that they neglect one treatment and then the condition gets worse. So the diabetes won’t be under control. And we want to understand wh y people like yourself are willing to proactively manage and take care of it. You go to see both professionals.

PARTICIPANT: cause if find that Tan Tock Seng is a good place. yah.

INTERVIEWER: how?

PARTICIPANT: the Doctor has been very good. The Doctor there. She’s a very good professional and the.. she’s looking after me very well.

INTERVIEWER: and have you been seeing the same Doctor there for the past 20 years or?

PARTICIPANT: no no. I was from Tan Tock Seng first, then I was sent to polyclinic. Followed up by diabetes, then I went back to Doctor Liew’s side again.

INTERVIEWER: who sent you to the polyclinic?

PARTICIPANT: the Tan Tock Seng people.

INTERVIEWER: So they wanted to be followed up in the polyclinic.

PARTICIPANT: yah

INTERVIEWER: ok. And how did you like that?

PARTICIPANT: At first I was worried, in case my diabetes won’t be controlled. But.. I accepted it.

INTERVIEWER: you accepted it?

PARTICIPANT: yah.

INTERVIEWER: ok. Why were you worried that the polyclinic will not be able to manage your diabetes?

PARTICIPANT: Because I feel that they were not good enough, really.

INTERVIEWER: can you tell me a bit more that? What you mean about that.

PARTICIPANT: my diabetes level will always very high. So, I felt that they were not professional enough for me.

INTERVIEWER: and then you went back to Tan Tock Seng for services

**Reference 2 - 4.37% Coverage**

INTERVIEWER: So do you think that it might be good if one professional can deal both your diabetes and your schizophrenia?

PARTICIPANT: yes, that the ideal condition.

INTERVIEWER: ok

PARTICIPANT: they can handle both. But I don’t know who to choose

INTERVIEWER: you don’t know which to choose?

PARTICIPANT: yah.

INTERVIEWER: do you have any other Doctors in your life? Any GPs or ..

PARTICIPANT: except for this Doctor Teo who was my family Doctor for years

INTERVIEWER: your family Doctor for years.

PARTICIPANT: the time we shifted to Clementi , that’s the housing estate. And the.. just walking distance. And the… from there till about my brother got married. And the… we saw other Doctors and. I know I saw him for my diabetes.

INTERVIEWER: or for schizophrenia?

PARTICIPANT: no, not for schizophrenia.

INTERVIEWER: cause we are curious to know if people with both conditions could be managed in the polyclinic.

PARTICIPANT: oh

INTERVIEWER: so these are... there are more professionals and it might be possible that

PARTICIPANT: I don’t think there are more professionals in the polyclinic.

INTERVIEWER: no?

PARTICIPANT: no.

INTERVIEWER: what do you think?

PARTICIPANT: I think the.. they are all fake Doctors

INTERVIEWER: so you prefer seeing 2 specialist

PARTICIPANT: yah

INTERVIEWER: in special places

PARTICIPANT: yah

INTERVIEWER: for each condition

PARTICIPANT: yah

INTERVIEWER: you wouldn’t be very happy about.. sort of being told to go back to polyclinic for those conditions.

PARTICIPANT: no, I wouldn’t be happy.

**Reference 3 - 1.51% Coverage**

INTERVIEWER: beyond, you know the.. so, you mentioned that it was difficult for you to choose. You din know who to choose. As sort of, if you get services from one place.

PARTICIPANT: no, you see. Cause I believe in specialisation. And I know one Doctor cannot specialise. They can manage the medicines. They call it … I forgotten the terms. They call a Doctor, who can examine both and see if there is any clash. So, I hope someone does that for me. But I don’t want one Doctor for both cases, because i don’t think that’s possible. Yah

**Reference 4 - 1.55% Coverage**

yah. You mentioned that you prefer Tan Tock Seng rather than polyclinic. What are the different types of services which makes you prefer Tan Tock Seng more?

PARTICIPANT: I think they are more professional there. the way they deal with diabetes condition.

2nd INTERVIEWER: ok, so. Because you mentioned that in polyclinic there was a dietician right?

PARTICIPANT: yah… but she wasn’t … that good la. She didn’t give me a good knowledge of food, you know. I only remembered like I say, her saying you go and doctorink diet coke or coke zero. You know.

**Reference 5 - 1.23% Coverage**

2nd INTERVIEWER: so, because you mentioned that you stayed at Simei care centre, at IMH and Hougang Care Centre. Is there any main differences between the 3?

PARTICIPANT: yes yes yes

2nd INTERVIEWER: could you explain a bit more?

PARTICIPANT: no I can’t. cause it’s a difficult word.

2nd INTERVIEWER: But you prefer to stay at SIMEI care centre

PARTICIPANT: n, I prefer HCC.

2nd INTERVIEWER: ok, HCC

PARTICIPANT: Hougang Care.

**<Internals\\HDL study service users HDL_151209-0140> - § 4 references coded [4.88% Coverage]**

**References 1-2 - 2.61% Coverage**

INTERVIEWER: cause one of the things we are trying to find out is if people such as yourself would prefer to have their GP or their GP at a polyclinic and not at IMH. Because you are stable now on your medication, so if your GP could give you the medication and you would have to come down to IMH, if that would be more convenient or if you prefer something like that. Or if people get something else out of coming to IMH.

PARTICIPANT: since Singapore launched the.. I stay I tampines, Tampines. There’s a polyclinic down there, last time I think 2004, there’s a clinic down there for psychiatric clinic. So because of the SARS period, they shut down the clinic. So I need to go IMH. Hahaha. Because of that. So, besides that, there’s geylang east there, aljunied MRT there, there’s a clinic for psychiatric la. Geylang east polyclinic down there. I was refer first at geylang east, then after that I was got relapsed then I was.. you know. Until now I was with IMH.

**Reference 3 - 0.84% Coverage**

INTERVIEWER: can you describe how the 2 places were different? Because you were at the geylang clinic for a while, yah?

PARTICIPANT: but over here is quite better ah. Other than NUH, other than Tan Tock Seng, other than all these hospital. Not because of the DR, because of my awareness to be recovered. Yah.

**Reference 4 - 1.44% Coverage**

2nd INTERVIEWER: how about verus Changi hospital. You were there for a while right? Can you say like what, why is the differences

PARTICIPANT: back then I was like…kind of frustrated because the hospital is very slow. Because it was slower than here. Because from 45 mins to half an hour you can see the psychiatrist. Over there is like 1 hour plus to see the patient. Because the slowness of their system, I think very long hours of queuing you know. Yah..

2nd INTERVIEWER: so that’s the reason you prefer here?

PARTICIPANT: yes

**<Internals\\HDL study service users HDL_151209-0149> - § 3 references coded [8.65% Coverage]**

**Reference 1 - 1.70% Coverage**

P: they didn’t really say, but I think they will refer me to the polyclinic, because like the previous time, they refer me to the polyclinic.

I: and if they do, would you got or would you ask them “could you treat me at IMH?”

P: if there is an option to treat here, I would prefer it to be the same; maybe what’s that, the scheduling will be the same, so maybe here would be more convenient than choosing polyclinic.

I: And it all comes down to schedule?

P: yeah

**Reference 2 - 3.38% Coverage**

I: because some people experience problems when they go to the polyclinic, they do not feel comfortable, thy feel that there is stigma against the mental illness, and they worry about going there, and they only want to come here, or some people worry about coming to IMH because there is stigma about coming to IMH, do you feel that there is anything like that that plays into why you choose one place or the other?

P: hum… I think… coming here is more comfortable, but also , like my mum doesn’t like it when we come to IMH for too many times, like when I come here for vocational training she is a bit upset because I have to come here 3 times a week , so there is some stigma, that if you go to IMH a lot , people will have a lot to say but I don’t find anything at the polyclinic because I don’t think they know, but even if I were to tell them, I am ok with it, because I am sure that they will keep it confidential. Yeah.

**Reference 3 - 3.57% Coverage**

I: what about preferences for other types of care? So if, do you have a GP?

P: I don; really frequent that,

I: don’t really have. But would you prefer if your GP could give you the treatment for schizophrenia, and monitor your cholesterol? So you would not have to come to IMH other than for the job club?

P: I think there is maybe the cost of the treatment; I think the GP is more expensive. Yeah so IMH is more, a better option for me.

I: what if the costs were equal?

P: then if it is close to my home I would go to my GP.

I: is there anything else in an idea setting where you could choose one or the other, you would think maybe a GP is better educated, different medication, anything like that?

P: sorry can you repeat?

I: that a GP may be better educated to treat, or have better access to medications to prescribe?

P: …. I feel like the IMH is … I feel that there is more… more facilities, is it the correct word? Whereas the GP doesn’t have that many services. Yeah

**<Internals\\HDL study_ service user HDL_151023_0040> - § 2 references coded [8.86% Coverage]**

**Reference 1 - 6.95% Coverage**

INTERVIEWER: and… do you think its ok to have polyclinic give you the medications for the diabetes and IMH give you medications for medical illness?

PARTICIPANT: no choice ah.

INTERVIEWER: no choice?

PARTICIPANT: no choice. They separate.

INTERVIEWER: if you have a choice, how would you like it?

PARTICIPANT: both together.

INTERVIEWER: why?

PARTICIPANT: see one place, then they will know what’s happening to my body. Now one person taking care of my body, one person taking care of my head. So, its no discussion.

INTERVIEWER: we are very interested in knowing what you think about this. So this is very important for us.

PARTICIPANT: I think it is not good la. I think it is better if everything is in one place. cause this people also Drs. Psychiatrist also Drs. You mean they can deal with diabetes. I don’t know whether they can or not. But they can try.

INTERVIEWER: so do you think that it will be better if maybe the polyclinic gives you the medications that they give you at IMH?

PARTICIPANT: yah, maybe.

INTERVIEWER: or would you prefer to have a psychiatrist give you the medications for diabetes?

PARTICIPANT: I prefer psychiatrist give medicine for diabetes.

INTERVIEWER: why?

PARTICIPANT: because I’m staying here. Convenient for me. I’m staying at hougang care center in blk 4, walking distance. Whereas if wanna go polyclinic, must take a bus. So its more convenient. IMH is more convenient for me.

**Reference 2 - 1.91% Coverage**

PARTICIPANT: yes. I don’t have many friends. So depend very much on my family.

INTERVIEWER: and do you think that they have preferences for where you get treatment?

PARTICIPANT: no. they don’t have. They don’t have preferences.

INTERVIEWER: So it’s most important for them to.. for you to get the care you need.

PARTICIPANT: yes

INTERVIEWER: rather than conveniences?

PARTICIPANT: yes

**<Internals\\HDL Study_service user HDL_151023_0035> - § 4 references coded [15.16% Coverage]**

**Reference 1 - 5.85% Coverage**

INTERVIEWER: but if given a choice, would you be seeking treatment at IMH down here or would you prefer other place where you can actually get treatment for schizophrenia and your…

PARTICIPANT: If talk about other place, another place ah., the best is my polyclinic lor. Tampinese polyclinic.

INTERVIEWER: that’s near your …

PARTICIPANT: I don’t need to travel so far.

INTERVIEWER: ok. But you have not gone to the polyclinic for your cholesterol check-up or anything like that?

PARTICIPANT: haven’t.

INTERVIEWER: ok. So now you say that if you were receiving treatment for schizophrenia at the polyclinic where it will be better right.

PARTICIPANT: yah.

INTERVIEWER: But. So, do you think that they will actually be able to provide good treatment for you at the polyclinic?

PARTICIPANT: I got no idea lei, this one. Yah

INTERVIEWER: ok. So right now you are not seeking treatment at the polyclinic. But supposed right. Come here for treatment of schizophrenia and you go for treatment for high cholesterol at polyclinic. Would you feel comfortable you getting treatment at 2 different settings or how is it for you?

PARTICIPANT: So.. I think its ok la. come here to do.. ok, receiving 2 different treatments from..

**Reference 2 - 5.51% Coverage**

INTERVIEWER: so, how would you feel getting treatment for your schizophrenia down here. And treatment for high cholesterol at polyclinic. Would you be comfortable getting treatment at 2 different places or how is it for you?

PARTICIPANT: ah.. if you talk about high cholesterol at polyclinic. Then I think it should be ok. Then come here for schizophrenia, yah. Ok la. I think ok with that. But the best is.. if.. if high cholesterol, what I heard from the DR is need to take medication right

INTERVIEWER: (agree)

PARTICIPANT: So, I will just… just… one place is also good la. I one shot take all the medication lah. Lets say 3 months then I take the medication. Then I come every 3 month or something like that.

INTERVIEWER: so you prefer to be all at the polyclinic if you can.

PARTICIPANT: yah, hopefully.

INTERVIEWER: hopefully

PARTICIPANT: yah

INTERVIEWER: but is there any particular reason that you want it at one place besides…

PARTICIPANT: convenience lah.

INTERVIEWER: but otherwise, nothing else?

PARTICIPANT: sorry?

INTERVIEWER: otherwise nothing else?

PARTICIPANT: convenience lor.

INTERVIEWER: convenience

PARTICIPANT: yah

**References 3-4 - 3.80% Coverage**

INTERVIEWER: so we are actually trying. Ok, so basically, the interview is trying to find out if your.. how you perceive the treatment you are getting for both schizophrenia and high cholesterol, in your case. But for you, you haven’t started seeing DR for high cholesterol yet.

PARTICIPANT: no haven’t. yah

INTERVIEWER: and you are ok with actually seeing 2 different Drs for 2 different conditions.

PARTICIPANT: hmmm.. I’m ok with that la. But hopefully I can just see.. I can just go to 1 place la. Because I know it’s a different thing. So, ah… I think, I’m ok with seeing 2 Drs la.

INTERVIEWER: you are ok. but convenient wise you want it at one place, is possible.

PARTICIPANT: yah la, convenient wise la. Then I don’t need to like.. this date go there, this date go there, like that.

**<Internals\\HDL Study_service user HDL_151210-0137> - § 6 references coded [17.97% Coverage]**

**Reference 1 - 5.53% Coverage**

INTERVIEWER: ok. So in that case, how is it that you have to go to Queenstown and Jurong polyclinic? Is it inconvenient for you to go?

PARTICIPANT: No, it’s all west area. In the west area

INTERVIEWER: so you are ok with that?

PARTICIPANT: ok with that

INTERVIEWER: but then if now you have a choice right, that you have to see in both conditions in one place. So either Jurong or Queenstown, would it be more convenient for you, or you don’t mind either way?

PARTICIPANT: don’t mind

INTERVIEWER: don’t mind either way

PARTICIPANT: yah.

INTERVIEWER: Because it’s both ?? (10:13)

PARTICIPANT: yes yes.

INTERVIEWER: ok, but hypothetically, let’s say that you are coming here for your psychiatric condition, right?

PARTICIPANT: yah

INTERVIEWER: and going to a polyclinic for your medical condition. Would you prefer that kind of arrangements?

PARTICIPANT: no choice lei.

INTERVIEWER: no choice.

PARTICIPANT: ah…

INTERVIEWER: ok

PARTICIPANT: I would like to, but it’s quite pretty far for me to travel.

INTERVIEWER: To go to 2 different places?

PARTICIPANT: no from.. let’s say buangkok green here and my house Jurong right, it’s very far apart. Yah…

**Reference 2 - 2.57% Coverage**

INTERVIEWER: for example you are seeing IMH for psychiatric condition right? Do you think that if you go outside at polyclinic you will get the same amount of care? Do you

PARTICIPANT: polyclinic is quite a bit crowded. And they like give me referral letter, they.. I call the hotline also, engaged. Keep on engaging. So.. and there’s no text message coming in to see Dr. They forgot to text me the appointment.

I:ok

PARTICIPANT: yah. Different polyclinic has different skills to handle. But I still prefer singhealth. Singhealth polyclinic.

**Reference 3 - 3.15% Coverage**

INTERVIEWER: So right now, do you have any particular preference about where you should be receiving treatments?

PARTICIPANT: based on DR.. can really help me see where I be seeking treatment. Maybe the Dr find that I comfortable, they at CWC ah..

INTERVIEWER: so it’s based on what the DR tells.

PARTICIPANT: yes yes

INTERVIEWER: but do you have any individual preference on where you like to get your treatment? Either for schizophrenia or hypertension.

PARTICIPANT: no

INTERVIEWER: no particular

PARTICIPANT: yah.

INTERVIEWER: so you think that either polyclinic or CWC, they will be able to treat your conditions just as well

PARTICIPANT: yes yes.

**Reference 4 - 2.02% Coverage**

ok. have you have any major problems since you started taking medication or started your treatments?

PARTICIPANT: some problems I encountered. Dizziness, drooling, ?? (18:07 )here and there. The whole.. the whole body is very lethargic. So I .. I push the Dr to.. 3 years back. the Dr quite friendly to me. Is a Japanese DR. yah. Then he slowly, he change my medication, then I get treated. He then.. they refer me back to CWC.

**Reference 5 - 2.30% Coverage**

INTERVIEWER: ok, so in the case that you had to continue seeing here. Would you have preferred that?

PARTICIPANT: continue seeing here?

INTERVIEWER: yah, for your psychiatric condition. Or would you have preferred to see another place?

PARTICIPANT: Maybe I go sayang to see.

INTERVIEWER: sayang

PARTICIPANT: yah, lesser people

INTERVIEWER: lesser people

PARTICIPANT: yah.

INTERVIEWER: so it’s the location of clinic B, that’s a bit problematic for you?

PARTICIPANT: a bit ah.

**Reference 6 - 2.40% Coverage**

INTERVIEWER: that’s why we want to get your opinion on what you feel about having to go to 2 locations or would you prefer 1 location?

PARTICIPANT: I prefer 1 location. I will see at CWC lor. (25:20)??Every day will see at CWC.

INTERVIEWER: so if you had a choice, it will be at CWC?

PARTICIPANT: yah..

INTERVIEWER: not at the polyclinic?

PARTICIPANT: CWC is a polyclinic by itself. If I have a choice, I’ll be seeing at.. everything at CWC lor. And.. it’s much more convenient to me also. In future..

**<Internals\\HDL Study_service user HDL_151218-0133> - § 3 references coded [10.42% Coverage]**

**Reference 1 - 4.58% Coverage**

INTERVIEWER: so you are pleased to come and get this information?

PARTICIPANT: yes

INTERVIEWER: so you don’t mind coming

PARTICIPANT: I don’t mind coming my off day, every Thursday.

INTERVIEWER: do you usually see this doctor? Or is it a different doctor each time?

PARTICIPANT: different doctor.

INTERVIEWER: ok. Would you rather be able to go to polyclinic to get this type of treatment when you feel unwell rather than have to come to IMH every time?

PARTICIPANT: I think it is ok to me. I go to polyclinic doctor; I think it’s ok to go to polyclinic or IMH treatment.

INTERVIEWER: but which do you prefer what do you prefer? Would it be better for you if the polyclinic could do what the psychiatrist does at IMH? Or is it ok for you to come to IMH and only go to the polyclinic when you feel unwell?

PARTICIPANT: I think it is better to come here. If I am feeling unwell, I go to polyclinic.2214

**Reference 2 - 1.58% Coverage**

INTERVIEWER: would you prefer if your polyclinic doctor could look after your medication, your condition, rather than you having to come to IMH to see a psychiatrist?

PARTICIPANT: … uhm… I think…I go to polyclinic is near, then I come here. If I come here when I have my first appointment, then it’s no problem.

**Reference 3 - 4.26% Coverage**

INTERVIEWER: good good. Now , what we are trying to understand is how people with your condition and diabetes receive treatment in two places. Is there anything that you want to tell us about your experiences that you think is important anything that is important to you about coming to IMH or seeing the polyclinic?

PARTICIPANT: I think it is important I come to IMH and polyclinic to see a doctor, because …the doctor let me that now I am no longer, not have diabetes, or not have high blood pressure, so if I go IMH or clinic appointment appointment date doctor tell me he wants to see me, if I am not fine to go to polyclinic, doctor understand I go to polyclinic…

INTERVIEWER: so if the doctor tell you to go to polyclinic you go to polyclinic?

PARTICIPANT: yes

INTERVIEWER: has the doctor her told you to go to polyclinic?

PARTICIPANT: no

**<Internals\\HDL Study_service user HDL_151218-0134> - § 3 references coded [14.90% Coverage]**

**Reference 1 - 4.94% Coverage**

INTERVIEWER: ok. So... he didn’t refer you out to see a GP or polyclinic? He didn’t?

PARTICIPANT: er… he did... actually wanted me to go see a GP. At the polyclinic. But then, I think I found it hard to go to the GP and polyclinic because... I found it quite troublesome. Yes, I have to make an special additional trip to the GP at the polyclinic to see the treatment for cholesterol. And in addition to the visit her. Cause if I… Cause I think I went... I think I went polyclinic for... they ask me take blood test again. And yeah. But I think is similar to like having the treatment at IMH. Cause they will just ask me to... they will just prescribe me the medications for cholesterol and then they will say they will need a review lor. Like... maybe three months later will ask you to do a blood test again. Yah so, coming to IMH I will also... I will just... get medicine and I will take it. Then they will also follow up on the... they will also follow up on the blood test. Yah. I don’t... I found it is quite troublesome if I need to have another additional visit to the polyclinic. Yah.

INTERVIEWER: and… So you find it troublesome to go to

PARTICIPANT: yah.

INTERVIEWER: the polyclinic additionally. so I guess you don’t prefer to see a GP or a specialist for high cholesterol and a psychiatrist for mental illness. You prefer to have a psychiatrist treat for both.

PARTICIPANT: yah.

**Reference 2 - 4.53% Coverage**

INTERVIEWER: so one of the things that we want to find out is how service users preferences guide their actions. And if they are able to do what they prefer. So some people who want to see the specialist psychiatrist here because it is the best type of psychiatric care. But they also want to see the best type of specialist care for diabetes or hypertension. So they don’t mind going to 2 different places, just to see 2 different physicians. And that’s their preference. And your preference is...

PARTICIPANT: prefer to see a ... see both ... all the conditions in a single settings. Ya. Because for me, is quite troublesome, If I go do a 2nd visit at polyclinic or specialist. Cause I don’t wait 2 appointments ah. Yah

INTERVIEWER: but now if your GP can follow you. Would you prefer to see the GP? And only go to the polyclinic and not bother coming to IMH. Or is it

PARTICIPANT: GP… I think the... don’t know. because I’ve been seeing the psychiatrist here for 13 over years. So, I’m quite used to the... yah. The environment

INTERVIEWER: and its always the same psychiatrist?

PARTICIPANT: yah, Dr Johnny.

INTERVIEWER: so it must be a good relationship then. to have with a

PARTICIPANT: not sure. I think is ah... comfortable la. Yah, I have been seeing him for 13 years.

**Reference 3 - 5.43% Coverage**

INTERVIEWER: ok. Any questions? One of the things we hope to do is understand as we said preferences. Is there anything that you think is important that we haven’t spoke about? That sort of might explain why some people like yourself don’t mind following up with the… one place and they don’t mind taking the medication?

PARTICIPANT: hmm… cause it is more convenient, this way. For me la. At least I feel that I don’t have to make 2 trips. Yah. And I think other thing is the location, which is quite near my house. So I don’t mind coming here also. Yah.

INTERVIEWER: so, if IMH wasn’t near your house

PARTICIPANT: ya, probably I would consider going to a GP for my schiz and cholesterol. Cause I know one of my friends she also... she was... she lives quite far away from IMH. So she having her follow up at the wellness clinic at... I think geyland. Geyland wellness clinic. So it’s nearer her house. Ya.

INTERVIEWER: so you could see yourself doing that sort of thing.

PARTICIPANT: yah, if it is quite far away from home.

INTERVIEWER: so its less about its expertise in IMH but more about the convenience of the location

PARTICIPANT: ya

INTERVIEWER: and there only one. What if your psychiatrist stopped prescribing the statins and you have to go somewhere else. You have no other choice, what do you think about that?

PARTICIPANT: my… I think I can go to GP at my... near my house there. I... I can still follow up at GP la. But I still prefer to have it prescribed at IMH. So I don’t have to go for another visit. Yah.

**<Internals\\HDL Study-Service User 140208-0106> - § 4 references coded [21.33% Coverage]**

**Reference 1 - 5.38% Coverage**

INTERVIEWER: ok ok. Very good. How did you feel about this way of splitting of services. Would you rather have Jerome treat everything, or the polyclinic treat everything. How do you prefer?

PARTICIPANT: erm. I think , because Jerome is only a psychiatrist. He is not a general doctor. So, its… it’s very difficult for him to give me the high blood pressure pill. It’s not .. he don’t study in that area.. So totally cannot la. The polyclinic doctor is specialized in general medication. So they can give the.. they can treat my high blood pressure la. So different.. because the IMH is more specialized in mental welfare and also the polyclinic is more general health care. General.. ya..

**Reference 2 - 4.59% Coverage**

INTERVIEWER: no. ok. and do you think that having to see 2 doctors. One at the polyclinic one here, has influenced your life in any way?

PARTICIPANT: influence, no. working perfectly fine. Because it is not a.. the period is quite long. 4 months plus one time ah. So it won’t affect my work ah.

INTERVIEWER: ok, so you don’t have trouble taking day off work to come like that. Ok.

PARTICIPANT: And also because my company is quite understanding. So they don’t.. he allow me to take time off ah. I.. ask my manager to give me time off on that day. Ask on advance ah. He.. agree la.

**Reference 3 - 8.15% Coverage**

INTERVIEWER: do you think care would be better for you, if polyclinic and psychiatrist were in the same place?

PARTICIPANT: same place. Ah.. can also. Cause it will save my time also.. Save my time of travelling. I have more time to rest also.

INTERVIEWER: But now.. if the polyclinic or if Jerome?

PARTICIPANT: Jerome.

INTERVIEWER: if Jerome could treat your metabolic comorbidity. Would you accept for him to follow you up? Or would you still prefer to see the polyclinic GP?

PARTICIPANT: ah.. also can la.

INTERVIEWER: and what about having follow up of schizophrenia only at polyclinic? Would you be interested in having that? Sort of..

PARTICIPANT: No, I think it would be better to have.. the… the IMH giving me the medicine and as well as the medicine at polyclinic. Because the polyclinic doctor may not understand my condition ah. My mental condition. Because they are not specialized in it.

INTERVIEWER: so, you would feel more comfortable treated only at imh for the 2 conditions, for your hypertension and schizophrenia.

**Reference 4 - 3.21% Coverage**

INTERVIEWER: Ya, anything you want to tell us about your experiences cause we want to know what it is like to be.. to get treatment for these conditions.

PARTICIPANT: its good la. It’s good that.. if the government managed to come up to a place ah.. like a place where we can get those treatment at the same time ah. It will save the conveniences for the people and also give us more time to ourselves also.

**<Internals\\HDL Study-Service User HDL_151203_0061> - § 4 references coded [6.81% Coverage]**

**Reference 1 - 1.59% Coverage**

Interviewer: Nothing. So currently you are taking medication everything from here only, ok but in the case that you were taking your medication for your schizophrenia down here and medication for your high cholesterol at the polyclinic, how do you…how do you find that?

Participant: I feel fine

Interviewer: You are ok

**Reference 2 - 2.47% Coverage**

So how do you feel …how would you feel if you had to get treatment from two different places?

Participant: No, no, ok

Interviewer: You are ok if you get it…

Participant: One place

Interviewer: One place, can you tell me why you will be comfortable getting it in one place?

Participant: More…more…more simple

Interviewer: More simple, can you tell me…

Participant: More simple to think…

Interviewer: simple to think…So you think it is more convenient?

Participant: Yah, more convenient yes.

**Reference 3 - 1.24% Coverage**

Interviewer: Hougang Polyclinic. Ok suppose right, suppose now you are getting all your medication from imh correct? But in the case that now the polyclinic treats you for you schizophrenia and high cholesterol, would you like it?

Participant: No, no

**Reference 4 - 1.52% Coverage**

Interviewer: Never ask. Ok but if you had to go to the polyclinic again for your high cholesterol, would you be ok?

Participant: Yes

Interviewer: You are ok going 2 places

Participant: Yah

Interviewer: But you would prefer one for convenience. Ok is there any other problems you face…

Participant: No

**<Internals\\HDL study-service user HDL_151209-0152> - § 5 references coded [6.14% Coverage]**

**Reference 1 - 1.35% Coverage**

PARTICIPANT: about.. last time I take risperidone. They change the medication in the ward. So, don’t know why. Everything the Dr on the ward control my decision want to leave the ward or not. very hard for me to take the decision I want to leave. Later I go to the ward, I want to go out, but they lock me up. It’s totally.. in the …the hands, the hands is totally in the Dr decision. If the Dr decision not really happy… (Laughs). If the Dr not feeling happy today, they will not give you to see him. Then say ok, he want me I feel not very happy. Second I will not discharge you.

**Reference 2 - 0.43% Coverage**

PARTICIPANT: So the decision is in the Dr hand la. Cannot. Last time I admit, I cannot celebrate Christmas, everything cannot. Christmas pass by, new year day pass by. Cannot go out.

**Reference 3 - 1.33% Coverage**

INTERVIEWER: and so you don’t see the Dr, you see a MO

PARTICIPANT: yah. Which I don’t like

INTERVIEWER: why not?

PARTICIPANT: huh?

INTERVIEWER: why not?

PARTICIPANT: not professional

INTERVIEWER: not professional

PARTICIPANT: anyhow give decision

INTERVIEWER: hmm?

PARTICIPANT: anyhow give decision. Anyhow give Decision!!

INTERVIEWER: ok. even though he is not professional, he make decisions.

PARTICIPANT: yah

INTERVIEWER: can you tell me a bit more on why you think he is not professional?

PARTICIPANT: (laughs). Mean he never make decision.

**Reference 4 - 1.34% Coverage**

INTERVIEWER: no, you don’t need take diabetes or take blood for diabetes. Ok. how do you feel about having to go to the polyclinic? vs. IMH. Do you think IMH is better than the polyclinic? Or was the polyclinic better than IMH?

PARTICIPANT: both the same ah.

INTERVIEWER: both are the same?

PARTICIPANT: ah

INTERVIEWER: why? Because remember you said that MOs sometimes not very professional. Yah? At IMH

PARTICIPANT: the MO never made any decision because ah.. he can’t make the decision by.., he say next time he want to talk to me, this one no need to talk to me.

**Reference 5 - 1.70% Coverage**

INTERVIEWER: you prefer seeing the Dr.. who’s the Dr you see? Dr.. Oh.. lin, Dr Yeo

PARTICIPANT: ah.

INTERVIEWER: you prefer seeing Dr Yeo. Yah, why do you prefer seeing Dr Yeo?

PARTICIPANT: Everybody say she’s good. (Laughs)

INTERVIEWER: he’s good?

PARTICIPANT: yah, ?? but I don’t know him. He come and approach me say ?? like that [incomprehensible]

INTERVIEWER: very good. Yah

PARTICIPANT: he keep on talking to me. Tell me I don’t know him like that. See Dr or not, then he come at me and say. Aiyah, so long. He say. You know why so long? They see Dr Yeo Sui lin. Cause Dr Yeo Sui lin like to listen to other people stories.

INTERVIEWER: so he spends more time with you? likes to listen to other people stories

**<Internals\\HDL Study-Service User_140113-0128> - § 1 reference coded [2.98% Coverage]**

**Reference 1 - 2.98% Coverage**

INTERVIEWER: which settings would you prefer to receive your treatment? Sort of where would you prefer treatment. In the polyclinic? Or? So let’s say if the polyclinic near your place is could provide you with the same treatments, now that you are stable on these medications as Dr Leong. Would you prefer that?

PARTICIPANT: I still prefer Dr Leong.

INTERVIEWER: why?

PARTICIPANT: he is.. as in he’s a psychiatrist. And then he has given me much support. And then.. I kind of feel.. how should I say? Because I know that I won’t get that kind of support and not that kind of support. but. I won’t be able to get that kind of treatment in polyclinic itself. Because what I need is a psychiatrist. And Dr Leong is a good one. A good one, yah. So, I think I would prefer that to be treated back here. Rather than in polyclinic. Yah.

**<Internals\\HDL Study-Service User_140209-0108> - § 2 references coded [6.78% Coverage]**

**Reference 1 - 3.42% Coverage**

INTERVIEWER: one of the things that we are trying to understand with this research is how people like yourself prefer to be treated. So, would you prefer your psychiatrist give you the medication for the high blood pressure and high cholesterol? So you would only need to come to one place? Or do you think it is best to have both one polyclinic and one IMH?

PARTICIPANT: I think I go to polyclinic and IMH for medication.

INTERVIEWER: you like that?

PARTICIPANT: that’s my opinion.

INTERVIEWER: ya, you prefer that?

PARTICIPANT: ya

INTERVIEWER: Why? What do you think?

PARTICIPANT: because, I’m definitely used to it already. And they have the.. its well taken care, by the doctors. And because have blood test and everything in one place also. In .. for my.. then for my eye sight, they test every yearly and my urine tests, and heart tests. Its very good. Very good. I mean service there.

**Reference 2 - 3.36% Coverage**

Oh, usually I find myself very hard sleeping and very strong medicine. That time until my husband was alive then he always like talk to me, calm me down. And he said I’m… asking me go for injection. To help me that I can sleep. Then I decide to come here and this tablet is very good. No need injection. I Dun want the injection. Very drowsy, not myself, very drowsy.

INTERVIEWER: the injection

PARTICIPANT: ya, the injection no good. Very drowsy, weak, very weak. Make me weak. Tablet medication better. Capsule one also too strong I think the powdery inside the capsule. The black capsule, very very strong. My sister said you look like a zombie, you walk. I mean very lifeless, not active at all.

INTERVIEWER: and how did you change the medication?

PARTICIPANT: After my husband passed away, I come to IMH here. I don’t know what year. I can’t remember. I very poor memory.

**<Internals\\HDL Study-Service User_140210-0112> - § 3 references coded [7.82% Coverage]**

**Reference 1 - 1.58% Coverage**

INTERVIEWER: so if given a choice, would you prefer to actually seek treatment for both conditions at one place or how would you prefer it?

PARTICIPANT: hm. I don’t mind.. like having treatment at one place. But, at nearby place my home. Ah, own home la.

INTERVIEWER: near your place.

PARTICIPANT: ya, near my place.

**Reference 2 - 1.58% Coverage**

INTERVIEWER: so, is this where you prefer to receive treatment for your condition? Do you think you are getting proper treatment down here or?

PARTICIPANT: so far.. I believe that I get a good treatment here. Ok because of the speciality of the department. Ok, for mental health. So, I don’t have a problem with it ah. Yah

**Reference 3 - 4.66% Coverage**

INTERVIEWER: but are there any particular reasons that you haven’t spoken to your polyclinic doctor about your psychiatric illness or is it that just because they don’t talk about it?

PARTICIPANT: Ah.. I don’t talk about it because.. I don’t think it is necessary to talk about it. Because I. they are only … dealing with medical conditions, so I just let it be that they just deal with my medical conditions like my psychiatric condition is separate place, separate person.

INTERVIEWER: ok. But then, in the case that they actually do talk about it. Would you be comfortable or would you still prefer this way. Having 2 separate places?

PARTICIPANT: I think I would prefer that. 2 separate places.

INTERVIEWER: 2 separate places.

PARTICIPANT: ya.

INTERVIEWER: is it just because of their expertise they have or? Any other reasons?

PARTICIPANT: erm. Due to the expertise also.

INTERVIEWER: due to expertise also…

PARTICIPANT: (agree)

**<Internals\\HDL Study-Service User_140214-0118> - § 3 references coded [9.54% Coverage]**

**Reference 1 - 2.02% Coverage**

INTERVIEWER: very good. And how did you feel about getting treatment for your mental illness here and your physical conditions at the polyclinic. Is it ok to be at 2 different place.

PARTICIPANT: no, the one I lose out is not able to go out to work. Because of my medical appointment I couldn’t able to work. Because I need time for my medical appointment and you know when you go out to work. People won’t give you time to see Drs, you know. They need you at work. So here IMH and polyclinic, I spend.. I’m actually going full strength. Full strength now. I’m seeing my polyclinic Dr , I’m seeing my IMH Dr. And I’m also trying to see a psychologist and I’m also trying to work in the.. to get a referral from the job club.

**Reference 2 - 5.33% Coverage**

INTERVIEWER: ok and now that you stable on those conditions, do you think you might be better only if you go to one spot? So the polyclinic can also give you your haloperidol? Or..

PARTICIPANT: ok, so that means just staying in polyclinic. But the polyclinic DR don’t have medicine for schizophrenia. Don’t have medication for that. Only more for the, what should I say. Other symptoms of sickness. Because the sickness that IMH treat is different from the sickness that polyclinic treat. They are different. Yah, they.. here is more specialised, mental illness. Because schizophrenia is like something uncontrollable that even I cannot tell myself, I cannot read myself. Only you can tell me what happened to me. I cannot understand myself. You need a 3rd party, yes.

INTERVIEWER: and how does that make IMH better than polyclinic.

PARTICIPANT: because you see schizophrenia at least there is a term. There is sickness, a term given to it. You are suffering from schizophrenia, or some kind of mental disorder. But for polyclinic, other people also suffering. High blood pressure.. what called… cholesterol, yah. Other people also have. But in IMH it is different, only people who have schizophrenia then they come here. Schizophrenia is a very broad name for mental illness. It is a very broad term. In the past when you have schizophrenia, you will be thrown, you know into some where. People don’t want to see you, you know. Now in the modern society, its different. People are seeking treatment and people are able to accept schizophrenia, they able to understand schizophrenia is a illness which can be treated. In the past, people maybe think that schizophrenia cannot be treated. So the patient, schizophrenia patient thrown into some where. In the forest or you know in the.. other countries the more traditional country, throw into the forest and then you know. Nobody will go near them like this. Yah.

**Reference 3 - 2.19% Coverage**

INTERVIEWER: ok, so would you prefer getting all your treatments at IMH?

PARTICIPANT: if can, if can, if possible. Because I remember there was a social worker that says that you cannot.. The Dr cannot prescribe panadol for the patient because that is not in.. like the IMH supposed to do, yah. So, you know because for example we need panadol when we get headaches. So we need the panadol when.. because sometimes as certain part of point of the day you just don’t feel well. The head is not well. So, not myself. I know of people who also take panadol, for sleeping patterns. For disorderly sleeping patterns. So the panadol is a very good medication for a lot of illness. Yah. So we know that we know that so we want the panadol. Because it can help us at any moment of time. Yes.

**<Internals\\HDL study-service user_151210-0147> - § 3 references coded [7.56% Coverage]**

**Reference 1 - 2.94% Coverage**

INTERVIEWER: cause… what we like to know is how people with schizophrenia and high cholesterol get treatment for both condition. Some people don’t like coming to IMH. So they go only to polyclinic. Or some people only want to come IMH, so they only want to come here. And they won’t go polyclinic. But you wouldn’t mind going to the polyclinic if they sent you for treatment.

PARTICIPANT: yah, I don’t mind.

INTERVIEWER: why not?

PARTICIPANT: although a bit inconvenient but… see hospital once and then once we have reason.

INTERVIEWER: so coming once a month is the reason. And you don’t mind.

PARTICIPANT: don’t mind

**Reference 2 - 2.37% Coverage**

INTERVIEWER: no, ok. Do you think IMH is a suitable place for you to get your treatment for schizophrenia and your high cholesterol?

PARTICIPANT: yes.

INTERVIEWER: or would you prefer to see a specialist for both?

PARTICIPANT: no.

INTERVIEWER: no?

PARTICIPANT: no, financially I cannot support.

INTERVIEWER: ah.. ok

PARTICIPANT: and so far so good. I am nothing. no, no. nothing happened in my life

INTERVIEWER: so you never have trouble getting services either here at IMH?

PARTICIPANT: yes

**Reference 3 - 2.25% Coverage**

INTERVIEWER: ah, ok. If another DR could have medifund, would you prefer going to another DR?

PARTICIPANT: no

INTERVIEWER: no? why not?

PARTICIPANT: so far I check here nothing wrong

INTERVIEWER: it’s ok

PARTICIPANT: yah.

INTERVIEWER: and is it more convenient for you to come here than to go to the polyclinic?

PARTICIPANT: yes

INTERVIEWER: why?

PARTICIPANT: if polyclinic, I have to travel. Don’t know which polyclinic they referring me to. So I’m not sure, which one.

**<Internals\\HDL_CG 151023_0036> - § 2 references coded [7.65% Coverage]**

**Reference 1 - 3.28% Coverage**

uhm if me, I would prefer elsewhere because I might be like you said, those people who hear that IMH is a bit like you know, yeah, so I prefer elsewhere actually if possible, like at polyclinic.

INTERVIEWER: but if you go elsewhere for other treatments of his condition, do you think that they could provide care?

PARTICIPANT: because this one we haven’t tried it , we don’t know if the service is there. Yeah. So maybe can try out for like three months if there is still not that good, then we can refer back here. If it is ok, then we can continue from there.

**Reference 2 - 4.36% Coverage**

PARTICIPANT: the only changes I would like to see is the doctor giving him more like , not only about medication and see him as a condition patient, but help …a service that could help him get better. Also more

INTERVIEWER: services that can get him better?

PARTICIPANT: yeah services that can get him better. I also don’t know la, because he seems that he doesn’t know about his condition, he knows he has a condition , but he didn’t go deep inside, like “how can I get myself well” like without medication like totally , “I’m ok” or slowly reduce, he doesn’t want and doesn’t like. Try to think of a way to help him to be normal, so that is why I am worried, because he always just thinks “ok I come here, take medication, go home” that’s it.

**<Internals\\HDL_CG 20160426 notes, no audio> - § 1 reference coded [7.90% Coverage]**

**Reference 1 - 7.90% Coverage**

Does not have a preference as long as her sister recovers but if there is need for her sister to get treatment for any HDL conditions at the polyclinic and psychiatric treatment at IMH, prefers to get both treatment at one place, preferably at IMH since sister has been seeing the doctor here for a long time.

**<Internals\\HDL_CG151209-0142> - § 1 reference coded [6.13% Coverage]**

**Reference 1 - 6.13% Coverage**

INTERVIEWER: ok another thin you mentioned was that sometimes when you had the blood tests done here at IMH if it was for the liver function test , you would ask them also to do the cholesterol test, since you were drawing blood, why do you prefer it done that way?

PARTICIPANT: because I want to get here to see the doctor is not easy, depending on mood

INTERVIEWER: so once again it is challenge of getting her to come and go

PARTICIPANT2: this is more the patient side

INTERVIEWER: yeas ok

PARTICIPANT: and then I even bring her to the police AnE, if she come here, or she don’t want to calm down , I ask police to help me, sometimes she will come after two weeks come, she don’t want to come for injection

PARTICIPANT2: she will say “you go for injection”

PARTICIPANT: I will ask for help, in the middle of the night I will ask her to come help me bring together to AE, very hard for the caregiver when the caregiver is me, I am not young, I am 60 years old, I also got angioplasty, so I am tired, I need help, that is why, I don’t have the strength, it is very , she make up her mind to don’t come, she don’t come.

PARTICIPANT2: strong willed

PARTICIPANT: strong willed yes

INTERVIEWER: do you think, if we talk about IMH vs. poly, do you think one place is better for her to get care for her high cholesterol? Because if she is only willing to go see few doctors, do you think it would be better for her to come see the IMH doctors and get everything treated by the IMH doctors, or to see the poly doctors?

PARTICIPANT: I think IMH better, one place better

INTERVIEWER: why

PARTICIPANT: because if two places, different places, so when she come here she no worry, she learn to go this place and that place

PARTICIPANT2: I think for metal patient somehow they have attachment, if they are used to this place the routine is there they more or less think “ok I am coming here”

PARTICIPANT: the hospital doctor here

PARTICIPANT2: it is a routine, but if we have to go to another place, sometimes they will feel “why am I seeing doctor so often” and then “You do I go to this place”

PARTICIPANT: angry

P2:”you go to see la” but here at least they say ok, sometimes they accept that they have mental illness , they accept that they need the medicine, but if “now you tell me that I got another disease I need to see another doctor in another place! You go la I am not sick, I am well!”

**<Internals\\HDL_CG151209-0158> - § 1 reference coded [0.73% Coverage]**

**Reference 1 - 0.73% Coverage**

INTERVIEWER: and so they give you all the medication here at once

PARTICIPANT: correct

INTERVIEWER: and is that better for you?

PARTICIPANT: Better! I got one stop, yeah.

**<Internals\\HDL_CG151209-0159> - § 2 references coded [5.29% Coverage]**

**Reference 1 - 2.46% Coverage**

PARTICIPANT: no I think it is better to see different doctors, yeah

INTERVIEWER: yes?

PARTICIPANT: yeah!!?!

INTERVIEWER: why?

PARTICIPANT: because each doctor is different, some will check thoroughly, some are more concerned and able to do their job. [Laughs] or what you call it , more understanding, I mean they will check in detail

INTERVIEWER: so better to have multiple doctors than being stuck with one doctor

PARTICIPANT: yeah in my opinion I think it is better, a second opinion is always better.

INTERVIEWER: true

PARTICIPANT: yes

**Reference 2 - 2.83% Coverage**

INTERVIEWER: ok… now what about worries about coming to IMH do you think it would be better for them to be treated in the polyclinic?

PARTICIPANT: for his case I think here would be better.

INTERVIEWER: why?

PARTICIPANT: because his is more serious than the sister you see, check the history and you see the history that is why they ask him to come to IMH rather than the poly.

INTERVIEWER: has he tried to go to the polyclinic for these concerns.

PARTICIPANT: do they have one at Changi? 1737

INTERVIEWER: I don’t know.

PARTICIPANT: yeah if they can transfer there, it would be nearer, rather than coming down here, so far.

**<Internals\\HDL_CG151210-0163> - § 1 reference coded [5.06% Coverage]**

**Reference 1 - 5.06% Coverage**

she actually, she also has; right now she also has appointments to CGH change general hospital for her heart cardio as well as her bones. Her heart is once every two months if I am not wrong or every 3 months, and for the bones once a year, for the bones and eyes and all that. The medicine that she is supposed to take from my understanding, IMH is only for the mental medications, whereas the diabetes I sunder CGH, but from what I know, she, she wants to take all the medication from here because of the simple fact that we receive subsidy at IMH but at CGH she doesn’t receive. So I mean coming from a normal paying family, she would go for his option. Because I think that she has 60% or 75%, so let’s say it is 100$ we only pay $25. Both my parents are no longer working , the three of us are working, so it is a bit of financial aspect that she decided to take all the medication here, but she still needs to do her medical appointment for the heart and everything at the CGH, so, there is two places , sorry not CGH, I mean polyclinic. So polyclinic. So these are the two places she often goes to, recently she was warded to CGH, last week , last two weeks , for one week in the hospital because of her thigh area suddenly felt very painful she was not able to walk, so when she was warded doctors check, and it relates to other things, so they checked her heart rate, we just had the doctor’s appointment earlier, so they, the CGH doctors asked whether some of the medicine that she is taking from IMH could cause some of the things that she is experiencing, so I understand that the doctors at IMH reduced one of the medications, because her hands both are shaking, and I think the doctors gave her medication to reduce the shakiness so they reduce the dosage of that, and hopefully by next month they want to review to see if it works to help reduce the heart rate, so if it does, and she is also able to control her shakiness, I think I is a good thing, because it is trying to balance between both. So that is what is happening to her right now.

**<Internals\\SP 140131-0096> - § 4 references coded [6.87% Coverage]**

**Reference 1 - 1.24% Coverage**

I mean ideally I guess in the best case situation we would actually have a physician in the hospital that could maybe see the patient on the same day that I see him. I would prefer that the medical condition is managed by a primary care physician first, but I guess where the physician is operating from is an important thing

**Reference 2 - 2.77% Coverage**

yeah we were talking about my preference as to would I be comfortable treating patients with metabolic conditions, I was saying that ideally it would be good if a physician can handle that part, the physical part, and whereas I would concentrate more on the mental illness. I guess in this case communication is very important between the physician and me as to like if they feel that their physical condition is deteriorating or not being well controlled because of the medication then maybe then I could do you know the necessary switching. But as to the treatment, as to what medication to start, uhm I actually wouldn’t be very comfortable in starting that, I would prefer for a primary care physician to do that actually

**Reference 3 - 0.04% Coverage**

preferences

**Reference 4 - 2.83% Coverage**

yeah once they have been started on medication for diabetes or dyslipidemia, yeah , I think ideally thy should still continue to see a primary care physician, on you know regular basis on their own schedule of course and depends on how often they need s to be seen because I think certain tests, certain monitoring, especially for diabetes, eye screening foot screening, are more easily done in the polyclinics, rather than in IMH, because, I am aware that we do such test here, but they are not done very regularly, they are only done once a week , and it is quite hard for our patients to be coming on different days to do these different test, whereas in a polyclinic it can all be done on the same day, and more closely monitored.

**<Internals\\SP_140109-0130> - § 2 references coded [2.76% Coverage]**

**Reference 1 - 1.81% Coverage**

I would prefer my way than what you were saying , what do you think? We continue on the treatment , I don’t prefer that because continuation of the treatment has a lot of other implications and consequences, in the sense we need to monitor other adverse outcomes to the kidney to the heart, which the at the moment here we do not have the facility unless we build it up, whatever it is polyclinic has better facility in that sense. yea

**Reference 2 - 0.95% Coverage**

uhm if the psychiatric condition is stable, and um the patient doesn’t have psychiatric problems or in the past, risk, generally I am quite comfortable to discharge to the polyclinic to manage the all the conditions, yup. So yeah

**<Internals\\SP_140116-0079> - § 12 references coded [8.68% Coverage]**

**Reference 1 - 1.52% Coverage**

**…hmm even I would prefer at least for the first few times patients seen by gp who can kind of choose the medication and then again I don’t mind seeing the patient again..hmmm so maybe the first few consultations or kind of it’s a joint collaboration with them so they come and the experts just like kind of help me decide a treatment plan for the patient given that there’s this risk .. and which patient… which medication may work and then give me kind of a plan on what should I do? how should I monitor?**

**References 2-3 - 2.71% Coverage**

**Now…er we’ve made it clear that sort of, some people don’t want to go to polyclinics to be treated, does that mean they don’t want to be treated at all or they prefer to be treated here? Because if we refer them to polyclinics**

**PARTICIPANT: I have seen both kinds of patients in my clinic so I have had patients who know that they have a problem they just don’t want treatment they don’t want to acknowledge it so it’s more denial**

**Interviewer: Ok**

**PARTICIPANT: Erm…And then there are some patients who don’t want to go to a polyclinic they they know there’s a problem but they rather be treated over here**

**Interviewer: They rather be treated here?**

**PARTICIPANT: Yah**

**Interviewer: Ok, are there any who don’t want to go to the polyclinic**

**PARTICIPANT: Who don’t ?**

**Interviewer: Who don’t mind going to the polyclinic? To be treated?**

**PARTICIPANT: Yah, we refer a lot to the polyclinic and they do go**

**Reference 4 - 1.25% Coverage**

**Ok. And why do you think some er would prefer to be treated here rather than the polyclinic**

**PARTICIPANT: I think some of them just their comfort level they are used to coming over here but as polyclinic is something especially erm those who have chronic illnesses like they don’t feel comfortable in an unfamilaiar place. It could be that they have faced stigma in going to these places so this is like more familiar…**

**References 5-6 - 0.63% Coverage**

**Maybe…maybe or sometimes…sometimes I’m the one because sometimes I have to tell them to go to a polyclinic. I tell them you know, this is not er my area of expertise I would prefer that you go to a polyclinic**

**Reference 7 - 0.02% Coverage**

**prefer**

**References 8-9 - 1.67% Coverage**

**I think it’s mainly the ease and convenience from patient’s point of view erm I think that is the biggest thing if you make it easier for them that’s where they will go so the patients who have like willingly gone to the polyclinic it would be because the polyclinic is actually next to their house so they would rather go there than come over here or they are already following up with the polyclinic with some doctor they’ll just go. The ease and convenience are the most important thing and I think we need to give them the option of where they would prefer**

**References 10-12 - 0.88% Coverage**

**Ok. Do you think there are any other considerations that we haven’t spoken about that might sort of explain why somebody prefers being seen at the polyclinic prefers being seeing here?**

**PARTICIPANT: Nothing …as I said it’s more the ease, convenience, familiarity, cost, all those basic issues**

**<Internals\\SP_140117-0080> - § 4 references coded [5.03% Coverage]**

**References 1-2 - 1.73% Coverage**

So one of the things we are trying to get to is the patient preference for who should be doing the follow-ups for metabolic syndromes so the psychiatrists typically the role is to consult on er mental health symptoms right? And once you’ve provided a treatment plan you might put the person back to the gp for follow-up treatment who do you think er should be responsible for the metabolic (11:09) sort of outside the range of psychiatric purvey?

Service Provider: I think responsibility of your er illness ah is the person themselves er should not be other people if the person can’t take responsibility who else can be responsible? the only way to have full control is lock them up in the hospital, make sure the diet is in control, the medication itself so if you talk about responsibility really I really think the patient themselves and caregivers that’s like I just got a call this person is needing palliative care so they are not hospitalized who’s looking after is the caregivers yah so the person is not well, the caregivers look after so I think if you…

**Reference 3 - 2.32% Coverage**

it is of course the patient’s service user’s choice what do you think influence s their choice to being treated either in the polyclinic of the gp or here at imh what do you think influences their preferences for treating for where they will be treated?

Service Provider: I think er individuals have different preferences some is convenience in terms of the location, the time, the effort, you know how to get all the treatment done right? So it’s the convenience some it’s actually because they have the relationship they only trust you so they will come because they only trust you and trust what you say they don’ trust anybody else so I think there is a whole spectrum so one is actually the convenience the accessibility but the other part is actually the rapport or the relationship you know and how we actually do it whether we do it efficiently and effectively lah it’s very difficult to measure effectiveness in terms of disease outcome prevention yah because it’s many years down the road so I really think the…we need we can offer them choice is it near their place or according to their routine because sometimes they may want to if they are working it’s not only near home but even somewhere near their workplace or even a company (accompanying? 16:29) doctor I think we need to think and see who are all the stakeholders and how we can collaborate right? Rather than oh we are responsible only person looking after

**Reference 4 - 0.98% Coverage**

usually when we don’t feel comfortable to treat we always refer out so I think today I actually made a statement we shouldn’t be keep referring people out because I can become a postman what? keep posting people out then where’s the treatment and where’s the relationship, there’s no relationship but if you’re the trusted advisor, you’re the trusted person basically it is to liaise rather than to refer yah right? so I tend not to use the word referral I tend to use request (19:30) request for this person right so who can i link this person with so that this person can get the most appropriate care?

**<Internals\\SP_140120-0082> - § 2 references coded [0.83% Coverage]**

**References 1-2 - 0.83% Coverage**

So I think if it’s just maintenance, treatment, I’ve got no trouble with managing those here in the IMH. But if it’s a new diagnosis, then my preference would be for the initial treatment to be started by the medical physicians, and then once it’s all stable, then I’ll continue on from here.

**<Internals\\SP_140120-0083> - § 3 references coded [6.15% Coverage]**

**References 1-2 - 3.72% Coverage**

What are your preferences for treating those metabolic syndromes?

Participant: Erm personally er this is a personal preference yah if you talk about adults in the past I’ve always felt that I’m a trained medical doctor so I can provide the care of course some sometimes in a busy clinic I wish I didn’t have to do additional steps like having to take blood pressure and things like that yah but I I actually feel it’s important to erm keep myself updated in terms of er you know er current treatments, medications because er even now when I have medical students sitting in my clinic I try to find out from them about how medical er treatments have progressed in this areas and er students are in fact teaching me and I feel that er you know for the one thing for y own personal kind of up keeping kind of medical training I will really love to be managing these problems then erm the other thing is that for esp the adult patients with little social support sometimes what happens is that erm they may actually default treatment for their medical problems because having to kind of go to diff care providers is difficult for them yah so erm I personally believe that if we have a one stop sort of a medical service for them then er the care I mean they they may not actually default as much you know er complying with er with follow-ups for those medical problems

**Reference 3 - 2.43% Coverage**

Er do you think that it is because the metabolic syndrome might be the result of psychiatric treatment that it is the psychiatrist’s responsibility to follow up with those conditions?

Participant: Well I don’t see so much because of responsibility and what not but partly because erm it’s about being able to provide a sort of a one stop complete er care for these patients especially for these chronic er lifestyle diseases er the capability to manage these conditions actually is kind of should be at least at a primary care level and I think er so although I’m not a a medical sort of specialist in any internal medicine but I should be able to provide at least a primary care level sort of treatment so why not for these patients who need them especially these vulnerable group yah who are more likely to kind of also be lost within the you know complex medical system healthcare system

**<Internals\\SP_140123-0081> - § 7 references coded [0.12% Coverage]**

**Reference 1 - 0.02% Coverage**

preference

**Reference 2 - 0.02% Coverage**

preference

**Reference 3 - 0.02% Coverage**

preference

**Reference 4 - 0.01% Coverage**

prefer

**Reference 5 - 0.01% Coverage**

prefer

**Reference 6 - 0.01% Coverage**

prefer

**Reference 7 - 0.02% Coverage**

preferences

**<Internals\\SP_140123-0084> - § 4 references coded [7.41% Coverage]**

**References 1-2 - 4.39% Coverage**

Participant: Hmmm… I think with the healthcare system in Singapore, erm…where the primary health care physicians and the specialists sort of psychiatrists don’t liaise that closely together I think it…it would be better that the psychiatrist is able to treat some of the comorbidities to a certain extent or to the level that they feel comfortable with but also to have say a certain network of gps that they can liaise with er because right now if they’re just followed up in polyclinics or private gps I think there’s no liaising between the specialists maybe the occasional memo…erm…but it doesn’t work hand in hand as it might do overseas sometimes where it’s…the roles are clear and they do liaise with each other and each appointment the other person is updated on what’s happened or the primary care doctors are updated on what’s happened erm…so I think the setting where er we do we should because we get patients who refuse to go to the polyclinic or gp and they don’t wish to and I think then we need to be equipped with managing these co-morbidities at least to a level where we say well it’s too complicated for us to manage now we need to bring in the specialist …er if not a po…gp or a polyclinic doctor but otherwise to have a system of gps who might be able to provide you know…a clinic here would…would maybe overcome that barrier of patients who refuse “no, I don’t want to see yet another doctor, I don’t want to go to a gp erm…why can’t you just prescribe me medicine?” so I often get asked that “why can’t you continue my medicine so erm it would be better … I guess the challenge is that I don’t feel to a certain extent to start medication for diabetes, or high cholesterol, hypertension the basic level I’m comfortable but when it, they are not responding or they need maybe multiple medication er to manage that illness erm then I start I don’t I’m not that confident so would have liked the support of a gp or a specialistwho we could work closely with then

**Reference 3 - 2.11% Coverage**

When it is the issue of sort of not wanting to go see multiple providers and them asking you “why can’t you just continue the medication”, do you feel that it is appropriate to…for you to agree to that sort of request to say ok we will…I’ll take it … just sort of renew your prescription

Participant: I think the hospital at one point we were… er but if I remember correctly I think ah..the hospital had said if at all possible don’t continue these prescriptions or other medical comorbidities we really should encourage them to see a…a specialist but if not then we can offer it here. So we, I do but still do encourage them to go see a specialist but yes for the handful of patients who just simply refuse to or are not keen to then we agree to maybe do the blood test monitor or continue the prescription , review it and then I do discuss that well… but if it is going up I will need to get a specialist erm…to see you so…it’s kind of … negotiation

**Reference 4 - 0.91% Coverage**

Ok…Would it sort of be a very similar explanation for the reason that some people prefer to be treated in the polyclinic versus those that prefer to be treated here?…have you experienced people who rather say “I’ll stick with…I’ll rather be seeing here…rather it’s…

Participant: No I don’t think that makes a difference er because then we are talking about stigma and I don’t think that makes a difference erm…

**<Internals\\SP_140123-0085> - § 1 reference coded [0.02% Coverage]**

**Reference 1 - 0.02% Coverage**

prefer

**<Internals\\SP_140125-0087> - § 5 references coded [0.16% Coverage]**

**Reference 1 - 0.02% Coverage**

prefer

**Reference 2 - 0.04% Coverage**

preference

**Reference 3 - 0.02% Coverage**

prefer

**Reference 4 - 0.04% Coverage**

preference

**Reference 5 - 0.04% Coverage**

preferences

**<Internals\\SP_140126-0088> - § 18 references coded [0.53% Coverage]**

**Reference 1 - 0.04% Coverage**

preferences

**Reference 2 - 0.04% Coverage**

preferences

**Reference 3 - 0.02% Coverage**

prefer

**Reference 4 - 0.04% Coverage**

preference

**Reference 5 - 0.02% Coverage**

prefer

**Reference 6 - 0.02% Coverage**

prefer

**Reference 7 - 0.04% Coverage**

preferably

**Reference 8 - 0.04% Coverage**

preferences

**Reference 9 - 0.02% Coverage**

prefer

**Reference 10 - 0.02% Coverage**

prefer

**Reference 11 - 0.02% Coverage**

prefer

**Reference 12 - 0.02% Coverage**

prefer

**Reference 13 - 0.02% Coverage**

prefer

**Reference 14 - 0.02% Coverage**

Prefer

**Reference 15 - 0.02% Coverage**

prefer

**Reference 16 - 0.04% Coverage**

preferences

**Reference 17 - 0.02% Coverage**

prefer

**Reference 18 - 0.04% Coverage**

preferences

**<Internals\\SP_140202-0098> - § 8 references coded [0.14% Coverage]**

**Reference 1 - 0.03% Coverage**

preferences

**Reference 2 - 0.01% Coverage**

prefer

**Reference 3 - 0.01% Coverage**

prefer

**Reference 4 - 0.01% Coverage**

prefer

**Reference 5 - 0.03% Coverage**

preferences

**Reference 6 - 0.01% Coverage**

prefer

**Reference 7 - 0.01% Coverage**

prefer

**Reference 8 - 0.01% Coverage**

prefer

**<Internals\\SP_140203-0100> - § 14 references coded [0.33% Coverage]**

**Reference 1 - 0.03% Coverage**

preference

**Reference 2 - 0.03% Coverage**

preference

**Reference 3 - 0.03% Coverage**

preference

**Reference 4 - 0.02% Coverage**

prefer

**Reference 5 - 0.03% Coverage**

preference

**Reference 6 - 0.02% Coverage**

prefer

**Reference 7 - 0.03% Coverage**

preference

**Reference 8 - 0.02% Coverage**

prefer

**Reference 9 - 0.02% Coverage**

prefer

**Reference 10 - 0.02% Coverage**

prefer

**Reference 11 - 0.03% Coverage**

preferences

**Reference 12 - 0.03% Coverage**

preference

**Reference 13 - 0.02% Coverage**

prefer

**Reference 14 - 0.03% Coverage**

preference

**<Internals\\SP_140209_0110> - § 2 references coded [3.49% Coverage]**

**Reference 1 - 1.81% Coverage**

ideology and view I would assume that patients prefer being seen in one place? Or prefer being seen here?

PARTICIPANT: yes

INTERVIEWER: do you have any sense that people would rather be seen in a different way or different setting? Or do you think that it is the patient’s preference to be seen by the psychiatrist for these conditions?

PARTICIPANT: I think that patient wouldn’t mind being seen by any single doctor. It could work the other way as well: that physicians see them for the psychiatric part of the illness and then treats them for their physical aspect as well. But I think for them the patient is more of a convenience thing, they really don’t want to go to different places, , if everything can be done under one roof, then that is what they would prefer. [End of recording 1]

**Reference 2 - 1.68% Coverage**

and it is primarily because of the stigma, which is associated with IMH. At times they do come back, one as I mentioned, they can’t be handled at other hospitals, and the second is the cost. So they have several relapses they go to private hospitals and they find that by the time they have the third or fourth relapse the family can’t afford treatment, so then they come back. So in that case the feeling of being stigmatized is still there, the desire of not really coming back to IMH is still there, but their options are limited. So they do come back., it is more against their will that they come back.

INTERVIEWER: it is less a preference and…

PARTICIPANT: yes less of a preference and more because there is no other option left.

**<Internals\\SP_140214-0116> - § 16 references coded [2.74% Coverage]**

**Reference 1 - 0.03% Coverage**

prefer

**Reference 2 - 0.03% Coverage**

prefer

**Reference 3 - 0.03% Coverage**

prefer

**Reference 4 - 0.03% Coverage**

prefer

**Reference 5 - 0.05% Coverage**

preference

**References 6-7 - 2.32% Coverage**

would it be better to be followed up by a psychiatrist once their metabolic condition is stable? Or would it be better for them to be followed up by a GP once the psychiatric condition is stable?

PARTICIPANT: uhm both is the depend on patient preference. I think can be seen both you know, if the condition of metabolic symptoms is stabilized and same time psychiatric condition is stabilized, it can be seen by any psychiatrist or GP, if the GP has the experience to handle the psychiatric patient.

**Reference 8 - 0.05% Coverage**

preference

**Reference 9 - 0.03% Coverage**

prefer

**Reference 10 - 0.03% Coverage**

prefer

**Reference 11 - 0.03% Coverage**

prefer

**Reference 12 - 0.03% Coverage**

prefer

**Reference 13 - 0.03% Coverage**

prefer

**Reference 14 - 0.03% Coverage**

prefer

**Reference 15 - 0.03% Coverage**

prefer

**Reference 16 - 0.03% Coverage**

prefer

**<Internals\\SP_140215-0120> - § 16 references coded [9.74% Coverage]**

**References 1-2 - 4.33% Coverage**

Or I guess you’ve said you’d preferred it if they would be able to continue seeing the polyclinic. But now have you ever encountered experiences where the patient has refused to go back to the polyclinic, or wants – pressures you to follow up, continue the medication?

PARTICIPANT: I must say so far actually (09:50) cause they may express some initial reluctance, but I think the ones they’ve encountered, there hasn’t been I think any serious kind of a…protest I think. About their going back. Because there may be lapses here and there. But usually they are kind of quite open to going back, and I think the crux of that is they know the rationale why they have to go back. So I find that that’s actually quite important. Because sometimes if they ask to just go back without the full realisation about the significance of that – why they’re doing it, then very often they may not be very keen to do so, or they may kind of slip in the middle. But usually I think after some kind of (10:32) slightly more prolonged explanation of why it’s in their best interest to basically be in the setting where they do this more regularly and they’re kind of more attuned to…the ranges and so on, and they could kind of react a bit more proactively. Usually there’s no problem. But sometimes we do do those tests, and we do do those screens over at our end. And we could kind of feedback to the primary physician about their blood monitoring parameters. Yeah, so yeah...i would say that’s been my experience thus far. (11:09)

**Reference 3 - 0.03% Coverage**

preferences

**Reference 4 - 2.64% Coverage**

I mean in the past I remembered when I was trained, I was told that I mean these negative conditions…because we are psychiatric facility we can treat to some extent – it’s kind of a…it’s almost kind of an advice that you know it’s better, and I can kind of understand the rationale for these monitoring to be done elsewhere where they see many more such cases. They are more sensitive and more tuned I think to thresholds whereby you need to really react so that was the context. But if the system is kind of slightly different whereby they are kind of mechanisms and support to basically then allow these cases to be seen here then I think then of course the practice I think can shift towards that and maybe it’s kind of more convenient for the patients as well. And that may also encourage greater adherence to their kind of follow up and then it will also kind of facilitate I think the monitoring the longer term as well.

**Reference 5 - 0.03% Coverage**

preference

**Reference 6 - 0.03% Coverage**

preference

**Reference 7 - 0.02% Coverage**

prefer

**Reference 8 - 0.02% Coverage**

prefer

**Reference 9 - 0.02% Coverage**

prefer

**Reference 10 - 0.03% Coverage**

preferred

**Reference 11 - 2.48% Coverage**

But it’s a kind of a natural process of care and perhaps the care relationships, that maybe over time they are familiar with the people. Perhaps that also kind of…persuade or kind of encouraged them to want to continue to see. I kind of remember a patient that I saw and I’m still seeing him. And he I think was at one point I think asked to go to a community clinic to continue his follow-up. But I think for various reasons I think he’s probably more familiar with the staff. Yes, so he kind of requested to come back, he has been happier I think coming back then…he always greet/scream/screen the staff, and myself of course (28:09). You know when he comes and…he doesn’t want to be referred out. So I think the familiarity and the process of having known the place for a long time. Probably kind of…added to that hospital-centricness I think, in some individuals.

**Reference 12 - 0.03% Coverage**

preference

**Reference 13 - 0.02% Coverage**

prefer

**Reference 14 - 0.02% Coverage**

prefer

**Reference 15 - 0.03% Coverage**

preferred

**Reference 16 - 0.03% Coverage**

preferences

**<Internals\\SP_151007-0097> - § 12 references coded [4.53% Coverage]**

**Reference 1 - 0.05% Coverage**

**preferences**

**Reference 2 - 1.38% Coverage**

for the comorbid conditions I don’t treat them, I … maintain the treatment if they are already on some treatment, very minor adjustments I may make, but I try to ensure that the treatment is continuing. Now if it is something like an incident problem, new problem, of course I refer them to a hospital or GP.

**Reference 3 - 2.78% Coverage**

INTERVIEWER: **ok, but at what level do you refer them? When it becomes serious?**

PARTICIPANT: the seriousness is determined by the blood tests.

INTERVIEWER: **so as much as you can you will try to treat, but you don’t actually go forward and start the treatment for these conditions**

PARTICIPANT: no the treatment cannot be started without having someone with the blood parameters indicating that there is a need for treatment

INTERVIEWER: **yes, but once they indicate that you need treatment for that?**

PARTICIPANT: obviously there is no doubt that I refer them to the

INTERVIEWER: **GP**

PARTICIPANT: gp or general hospital

**Reference 4 - 0.03% Coverage**

**prefer**

**Reference 5 - 0.03% Coverage**

**prefer**

**Reference 6 - 0.04% Coverage**

**preference**

**Reference 7 - 0.03% Coverage**

prefer

**Reference 8 - 0.04% Coverage**

preference

**Reference 9 - 0.03% Coverage**

prefer

**Reference 10 - 0.03% Coverage**

prefer

**Reference 11 - 0.04% Coverage**

preference

**Reference 12 - 0.05% Coverage**

**preferences**

**<Internals\\SP_151210-0132> - § 11 references coded [2.74% Coverage]**

**Reference 1 - 0.02% Coverage**

prefer

**References 2-3 - 2.53% Coverage**

So but the end of the day is to keep the patients within the system so we have a better sense, we still have to make some space for patients who would prefer to see their own family physicians, back home or in other polyclinics. For that I think if we want to have a good view of how all our patients are doing we have to tap on the electronic medical record. So the concept is to see all our patients as ours, the whole populations that’s one population. Stratify them , all the schizo patients who come to us as one population stratified into several groups, those seeing, those without metabolic medical problems, and those with into several groups, and those seen within IMH either the general psychiatric clinic or general medical clinic, and those seen outside.

**Reference 4 - 0.02% Coverage**

prefer

**Reference 5 - 0.02% Coverage**

prefer

**Reference 6 - 0.02% Coverage**

prefer

**Reference 7 - 0.02% Coverage**

prefer

**Reference 8 - 0.02% Coverage**

prefer

**Reference 9 - 0.02% Coverage**

prefer

**Reference 10 - 0.03% Coverage**

preference

**Reference 11 - 0.04% Coverage**

preferences
