# Supplementary material for: The Impact of Stakeholder Preferences on Service User Adherence to Treatments for Schizophrenia and Metabolic Comorbidities
Source: PLoS One. 2016 Nov 16;11(11):e0166171. doi: 10.1371/journal.pone.0166171 (PMC5112999; doi:10.1371/journal.pone.0166171)
Supplement: S1 File — This file contains the nodes used to construct the themes reported in the manuscript. Including advice to others; expertise; insight into illness; instructions; looking after kin; preferences; relapse; resistance to doctor’s orders; social factors; social support; stigma; therapeutic alliance; and uneasy about initiating treatment. (ZIP) [file pone.0166171.s001.zip › Qualitative data/Relapse.docx]

**Name:** relapse

**<Internals\\HDL interview 1 20151009144255082 no audio> - § 4 references coded [14.85% Coverage]**

**References 1-2 - 5.70% Coverage**

On aripiprazole, clozapine and haloperidol, and diazepam. Has been on these medications with 4-yearly reviews f0r 31 years. No relapse, no psychotic episode, no hospitalizations.

**References 3-4 - 9.15% Coverage**

-Taking 4 types of medication for Schizophrenia, claims that condition has gotten better, has not experienced relapse and is now able to sleep better as compared to before. Not taking insulin for diabetes. No change of medication over the years and has not experienced any major side-effects.

**<Internals\\HDL interview 2 20151111171452547 no audio> - § 3 references coded [6.91% Coverage]**

**References 1-2 - 4.15% Coverage**

Worries of relapse if he does not follow up the clozapine, so is very careful about following up with his visits. And was told he could come any day in emergency.

**Reference 3 - 2.76% Coverage**

Care givers play an important role monitoring the mental state of people with mental illness to spot relapses.

**<Internals\\HDL interview 3 20160217171017621 no audio> - § 1 reference coded [3.20% Coverage]**

**Reference 1 - 3.20% Coverage**

Had a recent relapse after the death of her sister. Lost her father shortly after.

**<Internals\\HDL study - service user HDL_151210-0153 (chinese)> - § 1 reference coded [2.00% Coverage]**

**Reference 1 - 2.00% Coverage**

Ｐ:　看医生、 看那边、等一下我又住院又来、又看。。又来这边的药咯。(see dr, see there, wait awhile and I was hospitalized, and see. And some here for medication)

I: 你又住院啊？(you stayed here again?)

P: 啊(yes)

I:你几时又住院的？(when you stayed here again?)

P: 去年啊(last year)

I: oh、 OK OK。 可以告诉我吗？ 还有什么东西。 (oh, ok ok. Can you tell me ? anything else)

P: 什么东西？ (what thing?)

I:你在这边住院了多久、 为什么会到这边来拿药、没有再回去polyclinic ？(you stayed here for how long, why you come here take medication and did not go back to polyclinic)

P: 住两个礼拜咯。 (stayed for 2 weeks)

I: 住两个礼拜啊(stayed for 2 weeks)

**<Internals\\HDL study - service user HDL_151222-0138> - § 2 references coded [2.46% Coverage]**

**Reference 1 - 1.37% Coverage**

Dr ask whether I want to get 3 months medicine or not, instead of every month come and work. Come and see Dr. I say cannot la. Like that, I tend to, after I forget everything. That’s what I don’t dare to try it. The Dr arrange us, ask me want to go for 3 months medicine. No no every time come, like that ah. But I said I cannot accept it. I scared the feeling come, maybe within 3 months I got relapse already.

**Reference 2 - 1.09% Coverage**

INTERVIEWER: and what are you worried about what happened if you have visits only every 3 months? What makes you worried about that?

PARTICIPANT: I scared I forget to take medicine. I scared my mood swing again. And then I scared I might forget. Last time I scared relapse ah. I… I tell myself, one month, one month better.

**<Internals\\HDL study -Service user HDL_140211-0114> - § 9 references coded [5.28% Coverage]**

**References 1-2 - 0.80% Coverage**

But actually at 1 point someone say actually should bring hospital. So they brought me here. But initially it was more like a day visit. I had some like a vitamin Cs, something you know. But I think my condition deteriorate over the years. So 3 times I had to be admitted cause of relapses. And then so that 3 times was spent over 10 years. And then last relapse was in 2011. Yeah, so

**References 3-5 - 0.90% Coverage**

PARTICIPANT: yah very difficult to say. Cause I was just telling her my last appointment, the dr was quoting me with research studies. Saying, ok. Like they have research studies is ok. Saying, in 10 out of 10 patients, 5 have a relapse after a certain period and 9 had a relapse after certain period. So in terms of duration. Even for me like 4 years was my last relapse is actually a very short term to the Dr la. Very minimal.

**References 6-7 - 2.35% Coverage**

INTERVIEWER: and what was the cause of the 2 relapses? Was it because you sort of have problems with medications or?

PARTICIPANT: not.. not… maybe partly because I was busy working. So not too cautious of taking it, you know. Even though I went for my appointments so forth. But also is actually the same cause of how I end up in first place actually developed again over the years, you know. So, that’s why Dr actually try to alert me, you know. Can you recognise when is the symptoms will.. when the symptoms will cause, you know. You… In the first place you were here because of stress, so over the years actually is.. stress also 1 factor, so on my 2nd and 3rd relapsed came about, it was still cause of stress. So he had asked me to actually kind of identify. If stress is one of the factor, try to reduce. Say you know to try and prevent. So stress and some other issues. Like relationships and then monetary problems, you know. And so over the years it has improved and I kind of recognise this is actually the cause, the symptoms, which I try to minimise to prevent the relapse from happening.

INTERVIEWER: very good

**Reference 8 - 1.09% Coverage**

INTERVIEWER: we are very curious what we can do for people don’t want to go and then have more relapses and then they don’t want to seek treatment. I’m wondering if there is anything, any advise you would like to tell someone. Lets say you can speak to yourself 10 years ago before you have your relapses before you understood what the condition was. What would you want to tell yourself?

PARTICIPANT: I think I would have.. I think awareness would have been great. I think back then I don’t even know what mental awareness

**Reference 9 - 0.14% Coverage**

treated is better than having relapse you know. Secretly hiding away

**<Internals\\HDL Study Service User HDL_151209-0145> - § 4 references coded [3.16% Coverage]**

**References 1-2 - 2.07% Coverage**

INTERVIEWER: and have you have relapses?

PARTICIPANT: yes

INTERVIEWER: when was your last relapse?

PARTICIPANT: that was about 10 years ago I think

INTERVIEWER: 10 years ago your last relapse, ok. Can you tell me a bit why you went from the.. Why were you warded? This last… this last time.

PARTICIPANT: that was not really due to my relapse. It was due to a girl stealing money from my purse. I left the purse on the table, and I just walk into the kitchen and within 2 minutes and she stole $2 off. I’m very sure it was the girl. But the SIMEI care said that it wasn’t her. I called the police, and the .. they came and they left. I said ok la, I can sleep and all. And then I called the police again. They came and bring me to IMH.

**References 3-4 - 1.09% Coverage**

INTERVIEWER: yes, ok. So how do you feel about having to come so regularly for appointment, with your Doctors?

PARTICIPANT: that ok.

INTERVIEWER: It’s ok?

PARTICIPANT: yah its ok.

INTERVIEWER: its ok, ok. cause some people don’t want to. So they skip appointments, so then they rather medications and they have relapse.

PARTICIPANT: yah, that will happen to me about 10 years ago.

**<Internals\\HDL study service users HDL_151209-0140> - § 17 references coded [14.59% Coverage]**

**References 1-2 - 1.11% Coverage**

PARTICIPANT: cleaner, fruit and beverage, restaurants. The longest I ever did was in a restaurant for 1 year. From a part timer to a full timer. Then after 1 year, I have a relapsed. That was my last relapse I think back in 2012. Until now I, currently no more relapse. It’s my 5th relapse. From… I think, from the age of 19, I was diagnosed as a depression. So at the age of 24, I have paranoid schizophrenia.

**References 3-4 - 3.29% Coverage**

INTERVIEWER: can you tell me about your last relapse?

PARTICIPANT: that was in 2012. That.. Is it ok that I.. because I that time I was taking… I took drugs. Laughs…

INTERVIEWER: its ok

PARTICIPANT: it got me… I mixed around with friends, you know. We party, we drinks but introduced me to this methamorphine. It’s ICE la. So I took it. So it’s been like kind like get.. it’s not because.. before that I was get paranoid schizophrenia. Then I took ICE.. the lucky thing is I can I not addicted to ICE. Right now I’m ok. With it. I… don’t take anymore. So the thing is I get paranoid. Sometimes I get sleep. At night I can’t sleep. You know when I take the drugs la. Then, my mum called the ambulance, you know. Feel like somebody spying on me, it get. Because I .. from what I know that when you take methamorphine, ICE, you get worse, worse. You get paranoid, you know. Sleepless nights, you know. Then I decide not to mix around with the.. all those bad influence, all that. So that.. it’s a good thing for me la, I don’t get hooked, addicted by the ICE. Yah. That’s what the last relapsed I have. Before that I don’t do, take drugs. Because of by working and schooling, you know. Jumble up, mix. A lot of stress la.

**Reference 5 - 0.46% Coverage**

INTERVIEWER: so we know a bit about how you sort of discover you have schizophrenia, and we know about your last relapse. How did you found out you have high cholesterol?

**References 6-7 - 2.67% Coverage**

I mean for.. I think for.. I think 15.. maybe 15 years I’ve been in IMH for treatments for schizophrenia. I think the progression since the first time I was diagnosed, I thought I was having AIDS, because I was in change hospital. Then I was studying in ITE. Then I was having these tablets but not knowing my diagnosis. The Dr didn’t say anything. Then I was exempted in national service. That one I was like wondering how come I take medication. I need to take medicine? because I was like… around 3 times I stopped medication. For schizophrenia. Then they come the relapse. For the 3rd time, then the 4th time I was like… it’s not skipping medicine, I mean some sort of argument between me and my family la. So the ambulance came in. you know. And… the progression is quite good, since I… way back 10 years ago and now. I adjust myself around 8 or 9 like that, around 10. The very best is 10. Yah. Because I got to know my condition, and then a lot of support group I attend, you know.

**References 8-9 - 2.38% Coverage**

INTERVIEWER: can you explained to me how that worked out? Because then you stopped taking the pills then you relapsed 3 times. Can we speak about sort of how your experiences were back then? Because that’s very interesting to us

PARTICIPANT: back then I was like feel confused. Because, I had a problem you know. A relationship problem between my girlfriend of mine. So, actually the problems starts with having a girlfriend you know. A relationship I have problem with relationships. So right now I don’t have any relationships because of the incident happened, you know. Because I get relapse, because of her, you know. Sometimes she.. you know. I’ve been maintaining myself as a single la. Obviously. I can’t have a relationship, only I have friends la. Yah. Because of my emotions, sometimes I have to… you can’t 100% know, you know. You have to give in. Then you break up, yah.

**References 10-11 - 1.12% Coverage**

How did you think your life changed when you sort of had the diagnosis.. understood that you had that mental illness?

PARTICIPANT: Think when I understand that.. when I got the last relapse of me..mine, in 2012. I went to stepping stone for the 2nd time. My first time was in 2010 and 12. Then they actually guide you along because the… they tell me about schizophrenia a lot. They tell a lot, teach a lot of things.

**Reference 12 - 0.98% Coverage**

So because of the SARS period, they shut down the clinic. So I need to go IMH. Hahaha. Because of that. So, besides that, there’s geylang east there, aljunied MRT there, there’s a clinic for psychiatric la. Geylang east polyclinic down there. I was refer first at geylang east, then after that I was got relapsed then I was.. you know. Until now I was with IMH.

**Reference 13 - 0.86% Coverage**

2nd INTERVIEWER: I don’t really understand that part. You mentioned that IMH is isolated right? In that..

PARTICIPANT: no I mean, treating patients like you can’t go out from the ward, you know. Just stay inside for isolation like.. I’ve been staying for 1 month in acute ward everything and all when I got relapsed.

**References 14-15 - 0.43% Coverage**

PARTICIPANT: I didn’t ask the Dr. because the Dr change when I got my relapse. They change into that old medicine. The one that I take before, not the new one.

**Reference 16 - 0.70% Coverage**

PARTICIPANT: I experience my neck stiff, very stiff, I can’t even chew bread, soft bread you know. Kind of like. When the they give me a high dose when I was like inside the ward, after went back to my house, you know. After after… what is that? After relapse.

**Reference 17 - 0.60% Coverage**

but does that influence the way that you want to comply with medication or you want to keep yourself fit? So does it come into play when you think about taking the medication , make sure that you don’t have relapses again.

**<Internals\\HDL study_ service user HDL_151023_0040> - § 2 references coded [1.66% Coverage]**

**References 1-2 - 1.66% Coverage**

INTERVIEWER: and how did you come to the conclusion that it was sort of you have to accept it? In the beginning, do you find it difficult to accept?

PARTICIPANT: In the beginning, I was in denial. I din accept that I had the illness. But everybody keeps pushing me back here. If I miss my appointment, I get a relapse. Come back here again.

**<Internals\\HDL Study_service user HDL_151023_0035> - § 7 references coded [6.31% Coverage]**

**Reference 1 - 1.66% Coverage**

PARTICIPANT: it was when I was a… during my re-service yah… then I was.. I heard voices, then I panicked. Then I talked to NUH. NUH I think. Then after that they refer me here.

INTERVIEWER: so when did you..

PARTICIPANT: ah.. they give me the medication. Then I did not really take the medication. Then it relapsed. That’s why I came here la.

**References 2-3 - 1.29% Coverage**

INTERVIEWER: ok. but ah initially at the beginning of the interview you actually said that you had a relapse, right?

PARTICIPANT: relapse. Yah yah.

INTERVIEWER: that was because you…

PARTICIPANT: I didn’t take the medication

INTERVIEWER: you didn’t take the medication

**References 4-7 - 3.36% Coverage**

INTERVIEWER: who actually ah… who actually admitted you at NUH? How did you end up?

PARTICIPANT: NUH I think is the.. NUH is send by the during my re-service. Yah, I was sent to NUH la, by the.. during military vehicle la. The ambulance ah.

INTERVIEWER: so you were doing your National Service then…

PARTICIPANT: no no. re-service.

INTERVIEWER: then they sent you to NUH because you had a relapse then.

PARTICIPANT: no no no. not relapse. Relapse is.. ah.. that one.. relapse is when I was at home, then after that I relapse. Then I come to IMH with my uncle, I think. Yah. That 1 is relapse.

INTERVIEWER: So you had a relapse and came here.

PARTICIPANT: yah, came here. But I didn’t go to NUH.

**<Internals\\HDL Study_service user HDL_151210-0137> - § 2 references coded [1.24% Coverage]**

**References 1-2 - 1.24% Coverage**

INTERVIEWER: last year you were warded because of..

PARTICIPANT: because of some family issues.

INTERVIEWER: ok.

PARTICIPANT: yah

INTERVIEWER: so you were admitted

PARTICIPANT: it’s not a relapse, family so. Family issue.

INTERVIEWER: family issue, ok.

**<Internals\\HDL Study-Service User 140208-0106> - § 2 references coded [4.21% Coverage]**

**Reference 1 - 1.84% Coverage**

PARTICIPANT: ya. They give me treatment and luckily everything is under control. And ya.

INTERVIEWER: have you, had relapses?

PARTICIPANT: Yes, ah. 2 times ah. I’ve been warded 2 times ah. One time in normal ward, one time in blk 7.

**Reference 2 - 2.37% Coverage**

INTERVIEWER: was your ward 7 admission because of medication problem? Or why did you end up in ward 7?

PARTICIPANT: because I some issues ah. So, I stopped taking the medication. I think that I that time stopped taking medication for a short period of time. That’s why, relapsed then warded in ward 7.

**<Internals\\HDL study-service user HDL_151209-0152> - § 2 references coded [1.46% Coverage]**

**References 1-2 - 1.46% Coverage**

PARTICIPANT: got people say come to IMH, the medicine cannot change. Is true ah. The medicine they give cannot change. I ask Dr, say can you change my ah.. can you change my medication? He say cannot change. Wait I scared you… you relapse ah.

INTERVIEWER: ah

PARTICIPANT: ah, so cannot change. Everything cannot touch.

INTERVIEWER: no. Have you ever have a relapse?

PARTICIPANT: huh?

INTERVIEWER: have you ever had a relapse?

PARTICIPANT: ah

INTERVIEWER: you have, yah?

PARTICIPANT: ah, 2 years ago

INTERVIEWER: 2 years ago, ok. and you went to the hospital then, yah?

PARTICIPANT: no Christmas, happy new year

**<Internals\\HDL Study-Service User_140113-0128> - § 7 references coded [6.07% Coverage]**

**Reference 1 - 0.95% Coverage**

ok, myself I suffer from . I was first diagnosed with psychosis back in 2009 or something like that. Then in 2012, I was suffer a relapse then I was diagnosed with schizophrenia. Yah. Er.. actually I graduated from NTU in the year of 2007, with a bachelor of science.

**References 2-4 - 1.31% Coverage**

PARTICIPANT: there was once I suffer a relapse early in the year and that is.. it’s still called a relapse but there were minor noises around. And it’s because of breakdown of friendship. Relationship I have with my friends. So I actually told him that I was.. that I was having that episode then he changed.. yeah. He changed, the injection medication for me. Yah.

**References 5-6 - 2.86% Coverage**

But, there was the point I had back when I was in Mt E. and, after.. when I was in Mt E, I come back here to IMH. My mindset was switched, as in changed. I was thinking that because during that episode, during the relapses right. Major relapse, I was thinking of like hurting harming myself. With the voices, because the voices keep on saying that I’m useless. I’m worthless. Might as well die and something like that. So I was thinking like harming myself. But, looking back on it. But I was afraid at the same time. As in if I’m gone, my loved ones. My family, my parents would suffer. I would come back to bring that kind of suffering to my parents so I… My mindset sort of changed. So I was thinking like instead of wallowing myself in pity, I might as well go out and do something greater. Yah.

**Reference 7 - 0.94% Coverage**

INTERVIEWER: Dr Leong for you to come once every 4 months? How do you feel about that?

PARTICIPANT: I’m ok with that. Because I have been diligently taking my medication very morning.

INTERVIEWER: and you haven’t have any relapsed recently.

PARTICIPANT: no.

**<Internals\\HDL Study-Service User_140209-0109> - § 2 references coded [1.59% Coverage]**

**References 1-2 - 1.59% Coverage**

INTERVIEWER: Do you ever have any more problems? Any more relapse or hospitalisation?

PARTICIPANT: no relapse ah. But, I think some doctors say is correct ah. This medicine is… this is a chronic illness, it cannot be healed, it only could be controlled. So since if it can be controlled, the condition is not so good la. Although not so bad, but also not so good.

**<Internals\\HDL Study-Service User_140210-0112> - § 1 reference coded [3.47% Coverage]**

**Reference 1 - 3.47% Coverage**

Cause you are well, you haven’t had a relapse and all in a while. You managed to take medications but you functioned well, yes?

PARTICIPANT: I’m slowly trying to get away from the stigama. Don’t think too much about it and just go on with the treatment as per normal. Like, as though I’m going to the polyclinic to go for my.. my medical conditions check up. Ya. Ya, I mean so as to prevent myself from over thinking that.. ah.. this place is .. a place where I have to be.. I mean I have to be seeking treatment from the doctor for the next how many years. So, I can’t say that I’m totally well because I’m still under treatment unless OI am not taking any treatment for the past 1 year and maybe. Ya.

**<Internals\\HDL Study-Service User_140214-0118> - § 5 references coded [2.05% Coverage]**

**References 1-3 - 0.79% Coverage**

INTERVIEWER: so you are aware that if you do have a relapse, we call it, it might be a big problem.

PARTICIPANT: yes correct. Because I know of people who have relapse and they have taken knife to hurt people. Or they have done things which is.. you know. Out of the ordinary. Yes.

**References 4-5 - 1.26% Coverage**

PARTICIPANT: at first it bothers me because of the transport charges, that we have to.. you know. Incur. But I always tell the DR I want to commit myself to this proper treatment. Complete treatment. So that when I get well, I can do what my next stage of life can do. If I don’t… If I do half way, that means my treatment is not complete I will like what you said. Relapse. And when I suffer relapse, nobody able to take care of me, cause I’m on my own.

**<Internals\\HDL study-service user_151210-0147> - § 20 references coded [20.95% Coverage]**

**References 1-2 - 1.81% Coverage**

INTERVIEWER: and why are you not working now?

PARTICIPANT: because of relapse.

INTERVIEWER: because of relapse, ok. Can you tell me a bit about... what symptoms you have and sort of what types of?

PARTICIPANT: last time when I was… in army. I was a bit suspicious and I hear voices. So enter to the hospital, later on. Currently on is the suspicious. I go Thailand to take medicine.

**References 3-4 - 2.12% Coverage**

PARTICIPANT: first I was taxi driving.

INTERVIEWER: ok. And then you stopped that because you had another relapse?

PARTICIPANT: no, because of the work itself

INTERVIEWER: oh, because of the work of taxi drivers. Ok. And can you tell me about… can you tell me about your last relapse?

PARTICIPANT: the last relapse is due to my consistent flu. I cannot get cured. So I start suspicious, people try to harm me. So, I decided to go to Thailand.

**Reference 5 - 1.45% Coverage**

PARTICIPANT: I… after I go there, spend a few weeks there. I cure itself.

INTERVIEWER: ah, ok. So, it went away on its own.

PARTICIPANT: yah.

INTERVIEWER: very good. (laughs). And then you came back?

PARTICIPANT: I came back.

INTERVIEWER: and that’s when you have your last relapse

PARTICIPANT: yes

**Reference 6 - 1.44% Coverage**

INTERVIEWER: here at IMH?

PARTICIPANT: yah, IMH.

INTERVIEWER: and how long ago was that?

PARTICIPANT: ah, 2 months.

INTERVIEWER: 2 months ago? Ok, and…

PARTICIPANT: no. not 2months ago. About 2 months for curing the relapse

INTERVIEWER: 2 months that you were here. For curing the relapse. Ok.

**References 7-8 - 1.13% Coverage**

INTERVIEWER: yah. But were they giving you… what medication were you taking to cure your relapse?

PARTICIPANT: haloperidol

INTERVIEWER: again

PARTICIPANT: yah

INTERVIEWER: yah, and now you are taking it regularly?

PARTICIPANT: yes

**Reference 9 - 0.68% Coverage**

INTERVIEWER: and how did you discover that you have high cholesterol?

PARTICIPANT: after I enter the … the last relapse, I enter the hospital.

**Reference 10 - 0.56% Coverage**

INTERVIEWER: ok. And before you were warded for your last relapse. Were you still taking haloperidol?

PARTICIPANT: yes

**References 11-12 - 1.82% Coverage**

PARTICIPANT: hmm. Don’t have change, but not too much la

INTERVIEWER: not too much?

PARTICIPANT: yah.

I; can you tell me how?

PARTICIPANT: only when once relapse, I have to come back here and stay for 2-3 months

INTERVIEWER: but so far, only a few relapses, not many.

PARTICIPANT: only few relapse.

INTERVIEWER: because you mentioned that you were very stable

PARTICIPANT: yah

**References 13-15 - 5.08% Coverage**

INTERVIEWER: do you know if your relapse is.. Happened because you stop taking your medications? Or was it simply because of the flu this time?

PARTICIPANT: because I stop my medications

INTERVIEWER: oh, ok. Because some people don’t like taking the medications.

PARTICIPANT: yah

INTERVIEWER: why did you stop? It’s ok if you did, its ok.

PARTICIPANT: I think I’m ok, so I stopped

INTERVIEWER: ahhh… ok. So you no more.. you didn’t hear voices anymore, so you stopped taking the medications and then it came back

PARTICIPANT: yah

INTERVIEWER: yah. I think that’s something a lot of people experience. Do you think that it’s going to be reason for you to stop your medication again?

PARTICIPANT: no.

INTERVIEWER: no, why not?

PARTICIPANT: dare not already. After I get relapse again. I never stop anymore.

INTERVIEWER: you don’t want to what?

PARTICIPANT: never stop anymore

INTERVIEWER: you don’t want stop your medication anymore.

PARTICIPANT: yah

INTERVIEWER: no. So you have a clear understanding that there is a …that it does affect.

PARTICIPANT: hm

**Reference 16 - 2.32% Coverage**

INTERVIEWER: ok. Any questions? No? we trying to do.. we trying to help people who are like you, but who have yet to understand that they have to continue their medications. Or they have yet to worry about the consequences of high blood pressure. So, because you have learned that stopping the medications may lead to more symptoms and relapse. You want to continue taking the medication, yah. And because you were worried about high blood pressure and stroke, you take care of yourself. Yes?

**References 17-18 - 1.22% Coverage**

INTERVIEWER: hmm, yah. What about looking for signs of relapse. Does he know or does she know?

PARTICIPANT: my roommate is same, schizophrenia patient.

INTERVIEWER: ok. So do you talk about your illnesses?

PARTICIPANT: no, we didn’t

INTERVIEWER: no, ok.

**References 19-20 - 1.32% Coverage**

INTERVIEWER: yah. So you never have relapse because you ran out of pills.

PARTICIPANT: yah

INTERVIEWER: no, ok. Do you think that your… your desire to take the medication comes from your experiences or comes from what you’ve been told by the Drs?

PARTICIPANT: my experience.

**<Internals\\HDL study-service user_151210-0148 (chinese with english)> - § 1 reference coded [4.81% Coverage]**

**Reference 1 - 4.81% Coverage**

2nd: really? 那你之前你不是有讲说你有进出医院咯。 (then previously you said that you in out hospital a lot of times?)

P: 对， 一直进出IMH医院。 (yes, in out IMH hospital).

2nd: 为什么？ (why?)

P: 因为听到声音。弄到我整个。。 那时候我整个很疯溃。 好像xiao cha bo 这样。 好像发神经这样。Then 不可以。 一直进出进出，进出很多次。 (because hear voices, ,make me totally… that time totally crazy. Like a xiao cha bo (chinese word for crazy girl). Like crazy. Then cannot. In out in out, in out a lot of times)

2nd: 是不是你没有吃药？ 所以才进出进出？( is it because you did not eat medications? That’s why in out?)

P: 对， 那时是没有吃药。 (yes, that time did not eat)

2nd: 为什么没有吃药？(why did not eat?)

P: 因为他讲不要吃比较好。(because he say don’t eat better)

2nd: 谁讲不要吃比较好？谁讲的？(who said don’t eat better? Who said?)

P: 我自己讲的。(I said)

2nd: 你自己讲的？(you said?)

P: 啊。 不要吃药。 (ah, don’t want to eat medications)

2nd: 那改次还敢吗？(and the next time do you still dare?)

P: 不敢了。不敢了。( don’t dare . don’t dare)

2nd: 不敢了啊？(don’t dare?)

**<Internals\\HDL_CG 151023_0036> - § 1 reference coded [4.47% Coverage]**

**Reference 1 - 4.47% Coverage**

INTERVIEWER: if possible you wouldn’t want someone who is close to you to receive treatment right? But you have not actually tried going elsewhere to seek treatment, right?

PARTICIPANT: we haven’t tried other place because this is the major, it is quite a big issue for him, like if without medication I am scared he may relapse, so I dare not try any other , unless the doctor here feels that he is ok to refer back to polyclinic and to try for a few months, that way then, I’ll just follow because I am very scared that he will relapse. Yeah. Actually quite sure it is [inaudible] for us

INTERVIEWER: in what way?

PARTICIPANT: mentally like hope that he is well, so afraid that at any time he will suddenly relapse or collapse, and yeah it will be very stressful.

**<Internals\\HDL_CG151209-0142> - § 1 reference coded [1.81% Coverage]**

**Reference 1 - 1.81% Coverage**

PARTICIPANT: and then in 2012 she admitted again for about 3 months 3 whole months, yeah, until today, I think 2012, the doctor say , he say he want to do ECT for her, so we say can you try oral, because it is not proven that ECT can help so we asked can you have oral sample then the doctor say we can try [incomprehensible] so from then until today

PARTICIPANT2: they admit her for that period because they said they want to observe, because we are lay people, we are not that good in giving the feedback, so they want to observe her, so while that period that she was here, I think she was a bit stabilized, then they send her down to the other ward, there is a ward that is teaching you to be independent

**<Internals\\SP_140120-0083> - § 3 references coded [1.87% Coverage]**

**Reference 1 - 0.65% Coverage**

the 3rd thing is about unfamiliarity with the condition and also erm inadequate training so they may not be able to I mean they may not be confident they may not kind of er be aware about how to pick up early relapse and things like that…and

**References 2-3 - 1.22% Coverage**

Not really right now I mean maybe for other conditions but for schizophrenia very few and the past few times like I told you there were some gps who were quite willing to provide care but yah I remember that 1 gp who fed back after… after the problematic experience with this patient especially when the patient relapse and will just sleep on the floor of her clinic and I think she felt it was not possible for her to continue providing the care yah

**<Internals\\SP_140126-0088> - § 2 references coded [1.97% Coverage]**

**References 1-2 - 1.97% Coverage**

my sense is that because once they drop out we have no way to monitor them, so we do not know how he has been, so we, we know however that those who stop the treatment have higher chance of getting a relapse.

INTERVIEWER: yes exactly.

PARTICIPANT: so we only see them when the condition gets much worst.

INTERVIEWER: yeah.

PARTICIPANT: so one way forward is if we, the hospital can consider enrolling a primary care physician in a psychiatric setting, to tackle this issue of metabolic syndrome and schizophrenia,

**<Internals\\SP_140202-0098> - § 3 references coded [2.62% Coverage]**

**Reference 1 - 1.67% Coverage**

Honestly I don’t know… honestly I really don’t know because both models could work in the sense that a gp who treats a psychiatric illness should have some understanding on what it means to treat psychiatric illness and when to detect early warning signs of relapse so currently we do have a graduate mental health diplomas for gps to actually upgrade the skills in the mental health arena and for psychiatrist if you do want to treat you know medical conditions as mentioned previously you could be undertreating it or over treating it so you do have to keep up your skills and your…in the medical aspects ..so for both models it really depends on how enthusiastic or how zealous the doctor

**References 2-3 - 0.96% Coverage**

Er so far thankfully I have had very good experiences with gps most of the time when I do send them over for what you call the gp partnership scheme you know a lot of times they are being given the regular follow-ups and they are treated well and whenever there is an early warning sign of relapse so far the gps have been quite prompt in actually referring back the patients to us for follow-up

**<Internals\\SP_140209_0110> - § 4 references coded [3.42% Coverage]**

**Reference 1 - 1.54% Coverage**

The stigma part of it comes in the initial stages when we try to get them into the program, we try to psycho educate them, so there are a few people who do not want us because it is from IMH. In those case we don’t even end up seeing the patient. We don’t end up following up with the patient because they decide “we don’t want IMH” . at times it requires another relapse for them to bring them in., so they might typically, I am talking about those who have declined our services, they might have another relapse , go to another hospital, either they are very agitated or aggressive or suicidal, and they cannot be admitted to those hospitals because they don’t have closed wards

**Reference 2 - 0.94% Coverage**

So they have several relapses they go to private hospitals and they find that by the time they have the third or fourth relapse the family can’t afford treatment, so then they come back. So in that case the feeling of being stigmatized is still there, the desire of not really coming back to IMH is still there, but their options are limited. So they do come back., it is more against their will that they come back.

**References 3-4 - 0.93% Coverage**

the second thing is every time they have a mini relapse, they send the person back here. I mean that’s the understanding as well, if they have a relapse, anything happens they find difficult to handle, then they would send back here. Which at times doesn’t really work very well, because the feedback from the patients I have received is that also: “what is the point, the GP sees me only when I am well ?!”2714

**<Internals\\SP_140215-0120> - § 4 references coded [4.22% Coverage]**

**Reference 1 - 1.40% Coverage**

And if they are on antipsychotic medications – and quite a number of them are actually on longer term kind of follow-up – they do return I think for their follow-up, and then some of them relapses for various reasons, and then they are put back on or their medications have changed and hence I think the monitoring of the side-effects including metabolic side effects or motor side effects then become part and parcel of the clinical care that we see either in the ward or in the outpatients

**References 2-3 - 0.59% Coverage**

Other kind of differences…but I mean outpatients if kind of they are in a state of relapse, then that may sometimes colour the picture that may influence I think their insight. So their willingness to go back

**Reference 4 - 2.23% Coverage**

Is one more optimal than the other? Any opinion?

PARTICIPANT: (29:00) Maybe not I mean the…it’s really what is best for the patient right? I mean ultimately, in…some of these sectors I think are also quite dynamic. I mean they change over time. If they are in an acute state of relapse, I mean if they cannot be managed in the community because of various reasons – aggression, you know agitated behaviour, the family cannot cope, community cannot cope, services have been garnered and they just cannot cope – I think it would be important basically to think about the right side, and one of the sides would be of course in a hospital is a little bit more restrictive. But hopefully quick stabilisation, quick kind of…settlement of their acute kind of relapse. And then the…(29:47

**<Internals\\SP_151210-0132> - § 2 references coded [0.71% Coverage]**

**References 1-2 - 0.71% Coverage**

, I worked with patients with chronic schizophrenia in inpatient and outpatient settings. I’ve had to look after them when they were in remission as well as when they were in relapse.

INTERVIEWER: ok, what about the
