# Supplementary material for: The Impact of Stakeholder Preferences on Service User Adherence to Treatments for Schizophrenia and Metabolic Comorbidities
Source: PLoS One. 2016 Nov 16;11(11):e0166171. doi: 10.1371/journal.pone.0166171 (PMC5112999; doi:10.1371/journal.pone.0166171)
Supplement: S1 File — This file contains the nodes used to construct the themes reported in the manuscript. Including advice to others; expertise; insight into illness; instructions; looking after kin; preferences; relapse; resistance to doctor’s orders; social factors; social support; stigma; therapeutic alliance; and uneasy about initiating treatment. (ZIP) [file pone.0166171.s001.zip › Qualitative data/Resistance to doctors orders.docx]

**Name:** Resistance to doctors orders

**<Internals\\HDL Study - service user HDL_151224-0139> - § 1 reference coded [1.28% Coverage]**

**Reference 1 - 1.28% Coverage**

ok. And do you know why they ask you to get tested at the polyclinic?

PARTICIPANT: because I din went to the polyclinic… polyclinic for long time. For how many years, 3 years. They then ask me to. The psychiatric here ask me to go to the polyclinic for blood test and take medications.

INTERVIEWER: Do you know when?

PARTICIPANT: ah… last month.

**<Internals\\HDL study -Service user HDL_140211-0114> - § 2 references coded [1.74% Coverage]**

**Reference 1 - 0.80% Coverage**

and they had your physicians, your psychiatrist, have they change your medication?

PARTICIPANT: no, in terms of quantity yes.

INTERVIEWER: yes.

PARTICIPANT: in terms of duration yes, the types specifically no.

INTERVIEWER: ok

PARTICIPANT: I think they actually had kind of targeted at my condition exactly. And I guess this proved to be effective. So, I just go along with it.

**Reference 2 - 0.94% Coverage**

Then one of the side effect was my cholesterol was up. It was actually detected here. Actually because of the medication, so they took a blood test here at IMH and they found it was a bit on the high side. So they actually got a referral for me to go to poly. So there was a linked up through the system. He was able to see and asking me questions. And yes, it’s through all these, part and parcel of the medication. That’s why I wanna be off it la.

**<Internals\\HDL study service users HDL_151209-0140> - § 2 references coded [3.45% Coverage]**

**Reference 1 - 1.48% Coverage**

INTERVIEWER: right. So, can we sort of look in more details on what happened like that. So who tested you at IMH? Was it under Dr Leong supervision?

PARTICIPANT: yah, Dr Leong supervision.

INTERVIEWER: and, so then he gave you a referral to the polyclinic.

PARTICIPANT: the… I think the nurse gave referral letter. The nurse called me up, so I need to go polyclinic la. So he.. posted me a letter. The… cholesterol letter. So he told me, the nurse told me to go to polyclinic to check cholesterol level. But I didn’t. I didn’t went for the check.

**Reference 2 - 1.96% Coverage**

INTERVIEWER: but when you received the letter from nurse, asking you to go to polyclinic. Why din you go? By the way.

PARTICIPANT: because I think I can maintain myself to be healthy la. But I smoke a bit la. But actually I did was every single day right now, I drink coffee I skip breakfast and lunch time I eat a bit. So, right now I only eat once for rice only. For one day. Except lunch rice, then after that dinner noodles or bread. Sliced. So take care of myself la. Watch carbohydrates, watch the sugar levels and I’m not addicted to sugar or chocolate. But I’m addicted to coffee and smoking la. That’s all. I drink a lot of plain water. So far, the olanzapine never gain me weight. Because of, I watch what I eat. Yah.

**<Internals\\HDL study-service user HDL_151209-0152> - § 1 reference coded [2.62% Coverage]**

**Reference 1 - 2.62% Coverage**

INTERVIEWER: laughs. Yah. But ah..and so when you wake up late, you don’t take the quetiapine?

PARTICIPANT: got got got, I got still take. But I ask the pharmacy for advice, they say cannot ah. If you if you never take on time, can skip. I don’t want to skip. I still take

INTERVIEWER: you don’t want to skip

PARTICIPANT: I don’t want to skip, I don’t listen to the pharmacy. I still take. I don’t listen to the pharmacy. They are trying to say if I skip hor hor can’t skip, I collect the medicine I should take ma. Where can skip? They say never mind, skip. Skip is what?

INTERVIEWER: not take

PARTICIPANT: yah, don’t take. They say if they tell me 8am take, I never take maybe I 11am wake up. They say 4hrs don’t take ah. I say I don’t listen. I still take. They say skip the medicine. Don’t want to take the medicine. how come? I skip the medicine? Then they give me medicine, I don’t want to take. So, I take ah.

INTERVIEWER: why is it important for you not to skip? Why is it important for you to take?

PARTICIPANT: I don’t listen to pharmacy. Pharmacy say ah… skip. How come can skip? Maybe I got problem!?

**<Internals\\HDL study-service user_151210-0148 (chinese with english)> - § 1 reference coded [5.15% Coverage]**

**Reference 1 - 5.15% Coverage**

2nd: ok. 那时候是医生没有叫你出去polyclinic 看啊？(ok. That time did the Dr tell you to go polyclinic)

P: 没有没有。 在这边验的。 我每次在这边验吗。 医生叫我去polyclinic 看， 我不要。 很多人。 很难等。 两三个小时。 我不可以等啦。 (no no. test here. Every time I test here. Dr ask me to go polyclinic see, I don’t want, a lot of people, very hard to wait. 2-3 hours. I cannot wait.)

2nd: 所以那时后你有去polyclinic la. (so you did went polyclinic that time.)

P: 有有. 很久以前。 我不懂是多久。 去一次而已。 ( yes yes. Very long ago. I don’t know how long. Went once only)

2nd: then 就不要去了再回来啦。 (then you didn’t go and come back.)

P: 再回来这边咯。(come back here)

2nd: ok.

I: If the polyclinic wasn’t crowded, would you consider going there?

P: no no no.

I: why not?

P: a lot of people, cannot wait. 2 or 3 hours. Must make an appointment. Then want to see a Dr.. 2-3 hour, many people cannot wait. Very long time. Then I tell my Dr, say I don’t want. I want taking here. I check la. Check the blood test. All here, check. Then Dr say one year check one time.

**<Internals\\HDL_CG151209-0142> - § 7 references coded [13.19% Coverage]**

**Reference 1 - 1.92% Coverage**

How did you find out about her high cholesterol?

PARTICIPANT: oh I send her to blood tests (doctors), then the IMH they test the blood she has cholesterol, high cholesterol.

INTERVIEWER: so it was IMH that did the test to monitor for high cholesterol?

PARTICIPANT2: the first time that we found out that she had high cholesterol is at IMH? Because I think

PARTICIPANT: oh before that I was in poly brining her to check then she was borderline

INTERVIEWER: in the polyclinic?

PARTICIPANT: borderline. And then high cholesterol the doctor give her the medication, 40mg, but I was “no la” I say too high, so I asked for 20? The doctor said, can give 20, and then until today I think 2 years.

INTERVIEWER: is that for the treatment of cholesterol?

**Reference 2 - 1.65% Coverage**

blood test, so I bring her, very hard to get her to go to polyclinic one day she helped me [pointing to his wife] to bring her, so we did the blood test there.

INTERVIEWER: why is it hard to get her to go to the polyclinic?

PARTICIPANT2: it is hard to bring her because she refuses, she is a little bit temperamental, her mood, and if you tell her that I am bringing you to see a doctor, she may agree and she may not depending on her mood, so she refuses to go sometimes, even coming here [IMH] so we have to sometimes think of ways to not tell her but just bring her here, so she has no option, but sometimes she is cooperative like today.

**Reference 3 - 1.17% Coverage**

every 6 months they do blood test at polyclinic?

PARTICIPANT: at polyclinic and here also. But the blood test she refuse to go for consultation, last two weeks she should have gone, but she didn’t want to go so I have no choice. I don’t force her.

INTERVIEWER: so have you ever done blood tests here at IMH?

PARTICIPANT: yes a few times,

PARTICIPANT2: for cholesterol?

PARTICIPANT: sometimes for cholesterol sometimes for the liver, I am not so sure.

**Reference 4 - 0.69% Coverage**

PARTICIPANT: because it is not so easy to get her to poly ,

PARTICIPANT2: getting her to see doctor is not easy too often she will refuse and say I am seeing doctor too often , I don’t want to see doctor

PARTICIPANT: won’t go and very hard to go an won’t want to go

**Reference 5 - 0.96% Coverage**

PARTICIPANT: this month, she did not go because she said she did not want to go. So I have no choice to not take her. So I go…

P1: she had the blood test but two days , one week later, to see the result she refused to go…

PARTICIPANT: so I went without her, and they said no, at the polyclinic they cannot, they need the patient to be present, otherwise cannot, so I leave it.

**Reference 6 - 3.77% Coverage**

, people know about things there, so I was[incomprehensible14.44] it was 2 3 or serious, and said ok she can go to school but in the end, to the end I follow up and see, one day one of the doctors consultants from IMH took trip to there, and he gave a talk at orchard hotel about the medicine risperidone

INTERVIEWER: yes risperidone

PARTICIPANT: and he say the presentation, he is called doctor [name] he said, no you just take the medicine it would be very easy, I hear the doctor. After that I bring her to NUH, to follow for I don’t know how many years, after that , before that we were in the [hospital] they do ECT I don’t know how many times 4 quite a number of times, also the symptoms also like this, not help much, so I bring her, I went to the orchard hotel to listen to the talk about the risperidone new drugs, so I told the doctor at nuh to continue until I don’t know how many years, and then her take risperidone for how many years, and here [above the chest] has swollen a lot of water coming out, so maybe because of the medication

INTERVIEWER: medication

PARTICIPANT: and then the medication also not effect already la, it lost effect after that, maybe not , maybe the time the doctor asked me, she said the [incomprehensible] so I asked the doctor because she looked tired whether the medicine can reduce the dose, the doctor asked to observe her I said can, at the time I did not know much [incomprehensible] the doctor said yeah he can review one the mg

**Reference 7 - 3.03% Coverage**

INTERVIEWER: ok another thin you mentioned was that sometimes when you had the blood tests done here at IMH if it was for the liver function test , you would ask them also to do the cholesterol test, since you were drawing blood, why do you prefer it done that way?

PARTICIPANT: because I want to get here to see the doctor is not easy, depending on mood

INTERVIEWER: so once again it is challenge of getting her to come and go

PARTICIPANT2: this is more the patient side

INTERVIEWER: yeas ok

PARTICIPANT: and then I even bring her to the police AnE, if she come here, or she don’t want to calm down , I ask police to help me, sometimes she will come after two weeks come, she don’t want to come for injection

PARTICIPANT2: she will say “you go for injection”

PARTICIPANT: I will ask for help, in the middle of the night I will ask her to come help me bring together to AE, very hard for the caregiver when the caregiver is me, I am not young, I am 60 years old, I also got angioplasty, so I am tired, I need help, that is why, I don’t have the strength, it is very , she make up her mind to don’t come, she don’t come.

PARTICIPANT2: strong willed

PARTICIPANT: strong willed yes

**<Internals\\SP 140131-0096> - § 3 references coded [8.08% Coverage]**

**Reference 1 - 1.73% Coverage**

one thing we have, there is a bit of resistance to taking the blood test in the first place, probably not all the patients have their fasting bloods done at the correct time, a lot of them don’t like taking bloods, they give excuses, scared of needles or they are not free to come back for the blood test, it is quite hard to track them as well to track their metabolic status, but the ones who do take the blood test and they do have abnormalities,

**Reference 2 - 4.47% Coverage**

Compounded to that is, some of them are coerced into taking their medication by their family or by us, by the treatment team, sometimes, so they even more don’t see the point and they don’t think they have a mental illness, and plus they are putting on weight and if you tell them about taking the blood test, thy don’t, they just say “I don’t want it anymore” so that kinda effects the treatment, moving forward.

INTERVIEWER: and what about for sort of, uhm, therapeutic alliance?

PARTICIPANT: therapeutic alliance?

INTERVIEWER: yeah

PARTICIPANT: its’ a bit hard, you mean maintain rapport with the patient while they are trying to build a therapeutic relationship, so I is quite hard to take on a more paternalistic role as in telling them what to do, we try as far as possible to agree on certain things trying to explain to them why they need the medication, we don’t want to force it on them because we know once they go home, we can’t control it once they are home. They can easily stop medication … so uhm we don’t try and force the medication on them so, it is more reaching an agreement as to what kind of medication they want with the minimal side effect

**Reference 3 - 1.88% Coverage**

I think it is more their motivation, some of them are more motivated to address their health concerns compared to others, particularly in the young ones, they are more up to date with the current knowledge, they basically are more internet savvy and they do their own internet research, and they know how it works, what they need to do to keep up with their appointments, their treatments, so I think that make a bit of a difference, the younger ones are a bit more informed on what to do

**<Internals\\SP_140109-0130> - § 5 references coded [10.58% Coverage]**

**Reference 1 - 2.66% Coverage**

the problem is that most when they come to me and if I have to say immediately that I am going to refer you back to a GP, usually it would not work , they say “no I don’t want” and that is exactly the reason why they have not been seeking treatment for years, yeah um, and and and there that comes my role where I treat them convince them they have this situation which has to be treated, and showing that treatment does help and improve on some of the parameters for example their blood glucose and all that. And when I have a better rapport that is when they will believe me and they are willing to take the referral to go to a polyclinic.

**Reference 2 - 1.88% Coverage**

why they disengage in the first place? Uhm, it is very difficult to say because I usually don’t go in depth and ask why they disengage, a number of them actually came, they have not been seeking treatment for years, that is at least what I know. Yeah, some of the reason they say is I don’t have a problem, I tell them your records show you have diabetes, you were taking medication, it was controlled for a while , and they say no I don’t have a problem

**Reference 3 - 1.53% Coverage**

problem uhm? Usually what I do is especially in the older people, I make it concert I show them their blood pressure, I show them their blood glucose, I show them their lab results, and uhm… yeah, these are some of the things I do to show them and work collaboratively with them rather than to tell them that they have this problem, and actually works most of the time.

**Reference 4 - 3.01% Coverage**

I suppose it is still better than not having the blood results, what happened is that you are just tolled that you have this condition, you are not told or even shown what or why how it come to this conclusion, what was the result like how bad are the results compared to normal person, and patients had blood pressure that was so high I actually show them and say the normal one is this value and yours is so far away, and with the rapport I have with them and then I can explain to them, that with this type of condition , if you continue for a year or two , you are going to get yourself into trouble, strokes and things like that, and usually , not just showing them, with the rapport actually that is how it works, yeah.

**Reference 5 - 1.49% Coverage**

usually I will work toward them going to a polyclinic, so generally most of them “ no” yes there are situations where I’ll have to do that, and this is more common in older population, and in the community setting in the sense that we have home service where we visit patients at home, especially the older patients, and a number of them couldn’t leave home to

**<Internals\\SP_140116-0079> - § 1 reference coded [0.57% Coverage]**

**Reference 1 - 0.57% Coverage**

**I have seen both kinds of patients in my clinic so I have had patients who know that they have a problem they just don’t want treatment they don’t want to acknowledge it so it’s more denial**

**<Internals\\SP_140120-0082> - § 1 reference coded [1.75% Coverage]**

**Reference 1 - 1.75% Coverage**

I don’t…I think some of them don’t see the importance of it. I mean, unless it’s a condition that would make them feel very uncomfortable, for some reason. But if it’s – many of the patients that I’m seeing, they are relatively young, they’re in their 20s, so you talk to them about obesity, hypertension, high cholesterol, diabetes…it doesn’t make a lot of sense to me, there isn’t an immediate risk. Because the risk that we’re talking about it’ll only occur to them 20, 30 years down the road. So then…and if it’s not at a convenient place, less reason they would want to get help for that particular condition.

**<Internals\\SP_140123-0084> - § 2 references coded [4.07% Coverage]**

**Reference 1 - 2.31% Coverage**

then the occasional one like er recent experience I had was someone who had gone to another specialist in a different hospital er…to do an ecg for whatever reason but erm…they appeared very reluctant to show me the copy of the ecg ah…which I couldn’t access on the system but I realized from the parents talking the father just telling the mother “oh don’t don’t it’s ok, don’t show it” er and er…eventually I managed to ask for a copy and checked that it was ok but again I think there’s a reluctance of not complicating the picture or they feel I didn’t get a chance to ask because they were talking in…in a different language erm…so..yes…I’m not entirely sure why some might withhold but I also think some prefer, don’t like so I’ve had patients who don’t like to be on more medication that’s the key thing I think for those who do withhold information. So I’ve had patients with very high cholesterol who I advised maybe we do need to start some medication and they would say no erm…just because they don’t want to be on too many tablets.

**Reference 2 - 1.76% Coverage**

it’s not entirely clear because the cholesterol would have been really high and then it turns out along the way as I find out that from the family that maybe the patient refused all medication therefore they were just discharged from follow-up care there. that is rare though most of them tend to say “well, come back in a few months we’ll repeat your blood tests, we’ll follow you up but occasionally they seem to just say “well then ok, come back when you need to which means that then they’re not having any monitoring unless I decide to do the blood test and monitor

Interviewer: So it’s possible that if er…a patient is not compliant with what the polyclinic or the gp prescribes they’re discharged or they’re no longer followed up

Participant: Occasionally I’ve come across that yes…yah

**<Internals\\SP_140123-0085> - § 1 reference coded [3.57% Coverage]**

**Reference 1 - 3.57% Coverage**

It’s quite common in the common pool patients lah – like the community pool patients, like they will just come back every month for the injections or to top up their medications – to top up their supply. But when you ask about “Why do you think you need to take the medications?” Often a time, they wouldn’t say that they have – it’s for the mental illness – but they would say that “It helps me to sleep better. It helps me to reduce all the disturbances that I’m having.” (05:23). So it’s the symptoms that is making them to take the medications, because the medication helps them to control the symptoms in a way. But then I guess for more for hypertension, and hyperlipidaemia and all these illness right – the chronic illness – because there isn’t like any life-destructing symptoms per se? So they don’t see the point of taking the medications like, high blood pressure they are asymptomatic – they have no headaches, they have no blurred vision anything right, so…to them, like look unwell, like don’t have any symptoms, “why do I have to take the extra pills?” That’s what I gather the feedback from most of the patients lah. And that’s the (06:07) reason I guess why, you know, they come back to take medication and injections from the mental health side, but not the chronic illness. Because to them, they are – you know the reading is high, but so what? I’m still well and asymptomatic.
