# Supplementary material for: The Impact of Stakeholder Preferences on Service User Adherence to Treatments for Schizophrenia and Metabolic Comorbidities
Source: PLoS One. 2016 Nov 16;11(11):e0166171. doi: 10.1371/journal.pone.0166171 (PMC5112999; doi:10.1371/journal.pone.0166171)
Supplement: S1 File — This file contains the nodes used to construct the themes reported in the manuscript. Including advice to others; expertise; insight into illness; instructions; looking after kin; preferences; relapse; resistance to doctor’s orders; social factors; social support; stigma; therapeutic alliance; and uneasy about initiating treatment. (ZIP) [file pone.0166171.s001.zip › Qualitative data/Social factors.docx]

**Name:** social factors

**<Internals\\HDL interview 3 20160217171017621 no audio> - § 1 reference coded [4.72% Coverage]**

**Reference 1 - 4.72% Coverage**

Began treatment at Mount Elizabeth, but due to cost of treatment, she now receives treatment at IMH because of Medifund.

**<Internals\\HDL Study - service user HDL_151224-0139> - § 1 reference coded [2.12% Coverage]**

**Reference 1 - 2.12% Coverage**

INTERVIEWER: why do you not.. why would you prefer there over IMH?

PARTICIPANT: because it is convenient and its cheaper.

INTERVIEWER: cheaper?

PARTICIPANT: yes. I show them my blue card. This card and I can get the discount.

INTERVIEWER: ah…

PARTICIPANT: $16 only.

INTERVIEWER: so its cheaper

PARTICIPANT: they ask around.. I can get $14 for that. Yah. Discount.

INTERVIEWER: ok.

PARTICIPANT: ya

INTERVIEWER: so makes it cheaper for you to go there.

PARTICIPANT: yes.

INTERVIEWER: do you know why its cheaper there than here?

PARTICIPANT: ah,.. I don’t know.

**<Internals\\HDL study - service user HDL_151222-0138> - § 1 reference coded [3.05% Coverage]**

**Reference 1 - 3.05% Coverage**

INTERVIEWER: do you have a general practitioner, GP, family GP?

PARTICIPANT: no, I don’t have, I don’t need this kind. Because I’m jobless. I.. got no bank account. I don’t go for this thing. Cannot la. Wait wait cause, wait must do a lot of paperwork. Like one, that one, everything.

INTERVIEWER: can you tell me about the paperwork? What do you mean? Cause we don’t know.

PARTICIPANT: because, I just now say already. I read the newspaper, read the book. Like “brr.. brr…” (sound). I cannot catch up.

INTERVIEWER: but what paper work do you have to see the GP for?

PARTICIPANT: for example, got no money, apply for medifund. For financial assistant. Something like that la.

INTERVIEWER: So because of that, you prefer seeing Dr Jimmy? Or because you don’t want to see GP? Or you can’t see a GP?

PARTICIPANT: because money problem la. Cannot Cannot go and see a psychiatrist. Money problem la, cannot.

**<Internals\\HDL study -Service user HDL_140211-0114> - § 7 references coded [10.63% Coverage]**

**Reference 1 - 0.59% Coverage**

INTERVIEWER: but did the hospital helped you in any other way to develop better understanding of your mental illness?

PARTICIPANT: ah, understanding is actually more to the contact of the Dr. because initially I was think like a.. over the years I was.. cost issues. So we slack.

**Reference 2 - 1.27% Coverage**

That’s the best thing my problem is now like twice a year, once in 6 months lah. But I was telling her also, because of the hours, I am working in the office. I work Monday to Friday office hour, so here is also the same thing. For the clinic, so its very difficult to take time off, have to take leave. And then I don’t really ah.. share that I have this condition, therefore I have to go for regular treatment so I take leave on my own. Kind of thing lah. So of course if possible, if Saturday like today is good you know? Able to accommodate. But for clinics is a bit difficult, I understand. That’s why..

**Reference 3 - 2.04% Coverage**

INTERVIEWER: So why do u feel that you have no choice to get IMH and polyclinic treatment.

PARTICIPANT: well, is suppose cost is a factor. Of course I have some help, but yeah. I think time also. And then but not only in terms of physical time you have to spend time here also. But also it take time away from your other things you want to do you know. Then .. and then of course .. the so call secrecy. Nobody wants to.. more of my immediate family. Friends 1 or 2. I think you can count your fingers who knows what my condition is. I think for blood pressure is kind of ok to share, but for mental not really you know? So that’s the side issues whereby of course if I have a … say I wouldn’t want to have all these things. Want to have a normal life, perfect, healthy body, you know? With perfect health, you know. So that’s why I mentioned about no choice lah. But I had to be treated. I have this condition. This is the only recourse I have. Just have to go through it lah.

**Reference 4 - 1.79% Coverage**

PARTICIPANT: I think I would have.. I think awareness would have been great. I think back then I don’t even know what mental awareness

INTERVIEWER: sorry?

PARTICIPANT: awareness. Yah. Even we back then just go out and don’t know what mental issues kind of thing. And then the symptoms. And the Dr was like as I say like stress was a factor. So I was doing a stressful job then so if I could have pinpoint that stress could lead to a mental. I mean sometimes you don’t really more of if you have families who, or friends who had it earlier on like you know. Perhaps you can be more mindful of it you know. So that you won’t bring it upon yourself. So the awareness thing is part of, if way back then I was aware of, I would have noticed it. Stressful, and plus all these other factors in you know that could actually lead to a mental condition. Yah, then erm..

**Reference 5 - 3.41% Coverage**

INTERVIEWER: by personal factors you mean relationships.

PARTICIPANT: yah. Relationships, stress, finance. All this kind of combination you know. Which can lead to not being treated. In a way to talk, to share with so it kind of deteriorate and that’s what I end up being here. So I think if you have maybe some.. now I do make an effort to read up a bit. If I had done that, but I guess then you don’t feel the need to, you know. So on hindsight it’s another thing. So that’s why if way back then if I know more, if I read up more, aware more I could have recognised it myself so that I don’t come to this point. Right now, yah.

INTERVIEWER: So, it would have been nice for you to have a better understanding so that you could recognise it in yourself what is going on.

PARTICIPANT: that’s right. I mean mental is not the running in my family. So there was no alarm bells, to like alert me this is possible. You know like breast cancer can be genetic and so forth. You go for check up. But how often do people go for mental check up? Not compared to normal medical screenings. You know.

INTERVIEWER: that’s true.

PARTICIPANT: Sometimes, even when you do survey you know. You ask questions, attend some professionals take away , and they think “ooh I see from the surveys you do, you are potential for mental thing you know. But we don’t have that kind of thing”. So its like.. so that’s actually no no ring bells, no indicator that you are actually going this way kind of thing. So if way back then, if I had all those things, perhaps I have had help. Yah then.. in terms of advice, some.. I think depends on some people you know.

**Reference 6 - 0.65% Coverage**

But.. sometimes when you hit it, where it hurts the most. In term of pocket you know. If you do it, if have to be admitted to hospital which cost money. Ok medication, come here, transport, all these cost money. So it works in that sense you know. You not trying to be calculative or what. Just to be practical.

**Reference 7 - 0.87% Coverage**

Are you hysterical, are you.. cause danger to other people, to the public, are you screaming and so forth. I say no, I don’t have all those kind of thing. And she was requesting a report from the hospital which I have to produce for her. So I had to come down and it’s also costing. It’s not cheap. It’s a hundred over. And then if you want to go through a subsidy, it takes longer and my employer wants it immediate.

**<Internals\\HDL Study Service User HDL_151209-0145> - § 4 references coded [8.60% Coverage]**

**Reference 1 - 0.83% Coverage**

INTERVIEWER: and now you are seeing a psychiatrist at in clinic B?

PARTICIPANT: Not one particular psychiatrist. I’m seeing a MO, because the… I saw one for one year. That’s a lot of money, you know. Then ahh, after the first time when I was a outpatient, he said that I didn’t need him anymore.

**Reference 2 - 2.03% Coverage**

INTERVIEWER: who sent you to the polyclinic?

PARTICIPANT: the Tan Tock Seng people.

INTERVIEWER: So they wanted to be followed up in the polyclinic.

PARTICIPANT: yah

INTERVIEWER: ok. And how did you like that?

PARTICIPANT: At first I was worried, in case my diabetes won’t be controlled. But.. I accepted it.

INTERVIEWER: you accepted it?

PARTICIPANT: yah.

INTERVIEWER: ok. Why were you worried that the polyclinic will not be able to manage your diabetes?

PARTICIPANT: Because I feel that they were not good enough, really.

INTERVIEWER: can you tell me a bit more that? What you mean about that.

PARTICIPANT: my diabetes level will always very high. So, I felt that they were not professional enough for me.

**Reference 3 - 1.04% Coverage**

INTERVIEWER: Doctor Bhanu recommended you to go back to Tan Tock Seng?

PARTICIPANT: yah

INTERVIEWER: ok. do you know i he ever reveal your diabetes condition or ?

PARTICIPANT: I don’t think he did. I don’t think he knows anything about diabetes.

INTERVIEWER: he doesn’t know anything about diabetes.

PARTICIPANT: yah

INTERVIEWER: ok. you are right there. (Laughs).

**Reference 4 - 4.70% Coverage**

INTERVIEWER: now one of the final things that I would like to ask you is if you ever experience trouble getting services?

PARTICIPANT: yah. From Simei care. First, the warden there was nasty to me, towards me. I think she was jealous of me. So, I found that she made trouble for me by complaining about me. I’ve done nothing wrong to her. In fact, I bought twice, I bought food for her. This special food. One was mutton. One was mutton curry, because I know she doesn’t know about mutton curry. You know, that’s Indian stuff. And I’m Sri Lankan, we are more of the South Indian. So, I bought mutton mutton curry for her. Maybe she didn’t like it. I don’t know. Second time was roasted chicken. Maybe she didn’t like it. She should have told me about it. Don’t buy stuffs for me, you know. Then she make trouble for me and she… I don’t know what happened.

INTERVIEWER: ok. What about getting care or getting services at Tan Tock Seng. Have you ever get any trouble there?

PARTICIPANT: no, no trouble.

INTERVIEWER: no trouble, ok. What about here, IMH? Any trouble here?

PARTICIPANT: no

INTERVIEWER: no, ok. what about at the polyclinic, any trouble? Any trouble at the polyclinic?

PARTICIPANT: the polyclinic depresses me. Because it’s so dirty.

INTERVIEWER: ohh…

PARTICIPANT: it’s so dirty. It depresses me. The one at clementi. The one at Jurong was a bit cleaner. A bit, but it still depresses you. Both places depresses me.

INTERVIEWER: ok

PARTICIPANT: Tan Tock Seng, no. IMH also no.

INTERVIEWER: what about the staff? Any trouble with Tan Tock Seng staff? So they.. do they know that you have a mental illness?

PARTICIPANT: the Doctor there knows, yes.

**<Internals\\HDL study service users HDL_151209-0140> - § 1 reference coded [0.91% Coverage]**

**Reference 1 - 0.91% Coverage**

Yah. I had a hard time to tell myself that. In my family members also treat me as..”ohh, where you working now?” every Hari Raya will ask me. My family la, my siblings all that. Ask me where I working now and all these. Why you haven’t get married, you know, all this. It’s kind of like put me down. Laughs… pretty hard on me. Laughs…

**<Internals\\HDL study service users HDL_151209-0149> - § 3 references coded [3.07% Coverage]**

**Reference 1 - 0.55% Coverage**

I: some people worry about the cost, that they can have medifund here but not elsewhere. Are you on medifund or is it out of pocket?

P: yeah medifund

**Reference 2 - 1.00% Coverage**

so there is some stigma, that if you go to IMH a lot , people will have a lot to say but I don’t find anything at the polyclinic because I don’t think they know, but even if I were to tell them, I am ok with it, because I am sure that they will keep it confidential. Yeah.

**Reference 3 - 1.53% Coverage**

I: don’t really have. But would you prefer if your GP could give you the treatment for schizophrenia, and monitor your cholesterol? So you would not have to come to IMH other than for the job club?

P: I think there is maybe the cost of the treatment; I think the GP is more expensive. Yeah so IMH is more, a better option for me.

I: what if the costs were equal?

P: then if it is close to my home I would go to my GP.

**<Internals\\HDL study_ service user HDL_151023_0040> - § 1 reference coded [2.42% Coverage]**

**Reference 1 - 2.42% Coverage**

INTERVIEWER: is there anything you like to change? About the way that you are getting services from the psychiatrist at the polyclinic?

PARTICIPANT: Maybe.. maybe the halfway house, they must employ a caregiver to bring people for their appointments at Tan Tock Seng, Koh poo huat [KPH], hougang polyclinic. Even here, the paper work, place you go, how much injection you take, they must have a caregiver there. Some of them don’t have caregiver. I don’t have a caregiver. So some of them don’t have.

**<Internals\\HDL Study_service user HDL_151023_0035> - § 1 reference coded [2.55% Coverage]**

**Reference 1 - 2.55% Coverage**

INTERVIEWER: besides that, cost wise? convenience?

PARTICIPANT: sorry?

INTERVIEWER: cost wise, convenience coming here.

PARTICIPANT: what you mean cost wise?

INTERVIEWER: cost wise…

PARTICIPANT: ohhh, ok ok. if I come alone ah. Without my finance then I think is..I will take bus la. Then because I come with her, I take taxi.

INTERVIEWER: ok. But convenience how is it? Near Your place or how is it?

PARTICIPANT: ah.. I’m.. it’s ok la, quite ok.

INTERVIEWER: quite ok

PARTICIPANT: yah, I don’t feel any trouble coming here.

**<Internals\\HDL Study_service user HDL_151210-0137> - § 1 reference coded [3.23% Coverage]**

**Reference 1 - 3.23% Coverage**

INTERVIEWER: ok, but hypothetically, let’s say that you are coming here for your psychiatric condition, right?

PARTICIPANT: yah

INTERVIEWER: and going to a polyclinic for your medical condition. Would you prefer that kind of arrangements?

PARTICIPANT: no choice lei.

INTERVIEWER: no choice.

PARTICIPANT: ah…

INTERVIEWER: ok

PARTICIPANT: I would like to, but it’s quite pretty far for me to travel.

INTERVIEWER: To go to 2 different places?

PARTICIPANT: no from.. let’s say buangkok green here and my house Jurong right, it’s very far apart. Yah…

INTERVIEWER: but besides the distance travel, are there any other reasons that you find it inconvenient?

PARTICIPANT: no.

**<Internals\\HDL Study_service user HDL_151218-0134> - § 1 reference coded [9.23% Coverage]**

**Reference 1 - 9.23% Coverage**

INTERVIEWER: can we speak about how your family feels about you coming to IMH?

PARTICIPANT: oh… the thing… I haven’t told my family about my condition

INTERVIEWER: so your family doesn’t know that you have schizophrenia?

PARTICIPANT: they don’t know

INTERVIEWER: they know about...

PARTICIPANT: yah.

INTERVIEWER: ok. er... can we speak abit more about that? If you feel comfortable of course. Because a lot of the time we find that some people who have your condition have trouble managing their condition on their own. So they rely on family to take care of certain things. Remember appointments or managing medications. But you manage on your own.

PARTICIPANT: ya.

INTERVIEWER: so can you tell me about what sort of have led you to keep your family or

PARTICIPANT: I think... basically... I think... I think family stress is more of the... one of the factors that trigger off my condition. Cause I don’t... have much family support la. As in... I face a lot of stress during my junior college. Studies er... cause the time... the time my father was out of job. He din went to look for a job. I depended on... my mum’s friend to support us financially and so… my relationship with my father was quite bad la. and I think that time I got an argument with him. Yah... and I think he has... he… he actually beated me... he actually hit me on my head la. Like… a few blows and … sort of like…. And I also feel like… sometimes I feel that this may be the cause of my condition. And… so my relationship with him is like not so good lo.

INTERVIEWER: you got a very complicated...

PARTICIPANT: yah

INTERVIEWER: very complicated life

PARTICIPANT: yah. And because my mother also. My family also don’t have plan. Proper financial plan. My mum like... she likes to gamble. My dad give her all his salary. She won’t plan for the family and she would gamble all away. The money and so ah... when I was studying in university, I take up 2 loans to pay for my tuition fee and my daily expenses. And... after I graduated I... faced a lot of stress also. Cause I need... need to pay my loans, and I also had difficulties getting a job la. Cause I got some... I got... I have a lack of confidence in myself. I… I’m afraid that I couldn’t do the job... people... my co-workers would like start to criticize me or talk on me. So i… have this lack of confidence and fear of... plus the stress. I got the stress of paying my loans also. So yah. I din tell my family because I don’t think they... I don’t think they actually... I don’t think they can actually help me la. Because... part of the stress comes from the family

**<Internals\\HDL Study-Service User 140208-0106> - § 1 reference coded [1.91% Coverage]**

**Reference 1 - 1.91% Coverage**

INTERVIEWER: do you think care would be better for you, if polyclinic and psychiatrist were in the same place?

PARTICIPANT: same place. Ah.. can also. Cause it will save my time also.. Save my time of travelling. I have more time to rest also.

**<Internals\\HDL Study-Service User HDL_151203_0061> - § 2 references coded [2.30% Coverage]**

**Reference 1 - 0.91% Coverage**

Interviewer: Only come here, ok. So when you were at the polyclinic before right? Did you have any problems?

Participant: No, no

Interviewer: With the doctor, queues

Participant: No

**Reference 2 - 1.39% Coverage**

Interviewer: But you would prefer one for convenience. Ok is there any other problems you face…

Participant: No

Interviewer: Getting to imh… like distance? cost?

Participant: No

Interviewer: No

Participant: No

Interviewer: It’s a convenient place for you?

Participant: Yah

**<Internals\\HDL Study-Service User_140113-0128> - § 3 references coded [3.04% Coverage]**

**Reference 1 - 1.00% Coverage**

INTERVIEWER: ok. how do you feel about getting treated here at imh? verus Mt Elizabeth.

PARTICIPANT: Mt Elizabeth was expensive. Like for abilify back here, one pill here it cost $8. But in Mt E, it cost around $13-16 dollars. So I unhappy with the costs. So I shifted to IMH.

**Reference 2 - 1.01% Coverage**

PARTICIPANT: not really. Because I was having debts at the bank. Cost of like.. one and a half month of being at Mt E, cost to around $1000+ and I take the medications daily. I’m not able to cough up with the cost. So the cost is actually one big factor that I decide to come to IMH.

**Reference 3 - 1.03% Coverage**

INTERVIEWER: yah. Ok. so have you ever experienced problems getting treatment?

PARTICIPANT: no.

INTERVIEWER: what about the polyclinic? You have never asked you anything that makes you uncomfortable? Do you feel uncomfortable at the polyclinic or anything like that?

PARTICIPANT: no.

**<Internals\\HDL Study-Service User_140209-0108> - § 3 references coded [6.94% Coverage]**

**Reference 1 - 1.80% Coverage**

INTERVIEWER: ya. And when you were on those medication that were making you very drowsy. Did the Dr ask you to go for blood test?

PARTICIPANT: no

INTERVIEWER: no?

PARTICIPANT: no, he keep on.. I think .. don’t … Dr should help to for patient to cure. But.. I find that he is like money making. Ask me come back very often. 2 weeks, every 2 weeks, after 2 weeks come back get injection and very time high bill I have to pay. Very high, private 1. Very costly. Very costly.

**Reference 2 - 1.21% Coverage**

INTERVIEWER: and.. so this imh is also convenient.

PARTICIPANT: yah yah yah. That’s why I say.. actually for me clementi is very far away. Clementi from bus 1, if traffic jam nearly 2 hours reach here. I reach my mother’s house 1hr 20 mins, use the bus. But only 1 bus trip, a take me here. To my mother’s place also.

**Reference 3 - 3.93% Coverage**

INTERVIEWER: yup, what I’m curious about is if the doctor at the polyclinic want to know what is going on with the Dr at IMH. I want to know if they speak to each other or they have any interest in what one of them does. So, do the Dr here know that you go to the polyclinic?

PARTICIPANT: Dr from IMH?

INTERVIEWER: ya.

PARTICIPANT: they ask if you go to the .. they only recently that.. sometimes, they don’t ask. They don’t ask.

INTERVIEWER: but have they recently been asking?

PARTICIPANT: except Dr Lum?

INTERVIEWER: Dr Lum. Yah, Dr Lum ask.

PARTICIPANT: ya.

INTERVIEWER: ok. But otherwise do you ever tell them? That you are treated for diabetes or you are treated for hypertension, high cholesterol?

PARTICIPANT: oh, when I have a operation. I went to NUH. They ask me about my history, my medical history.

INTERVIEWER: ok

PARTICIPANT: ya. They know.

INTERVIEWER: otherwise, the DR at polyclinic don’t ask about IMH.

PARTICIPANT: ya ya ya.

INTERVIEWER: and IMH don’t ask abt…

PARTICIPANT: yah yah yah.

**<Internals\\HDL Study-Service User_140209-0109> - § 6 references coded [9.85% Coverage]**

**Reference 1 - 1.14% Coverage**

PARTICIPANT: hmm.. nothing much la, actually. But because last time I see is Alexandra hospital. He said why don’t go woodbridge, its cheaper.

INTERVIEWER: its cheaper at woodbridge?

PARTICIPANT: ya. I don’t know its very expensive. So after that I go IMH. ya

**Reference 2 - 3.10% Coverage**

INTERVIEWER: ok. And what about if the psychiatrist here gave you prescription for high blood pressure?

PARTICIPANT: ah... how about the payment?

INTERVIEWER: payment? Hmm.. what do you think?

PARTICIPANT: because I taking there is.. I didn’t pay because of my CHAS.

INTERVIEWER: here? What do you mean? I don’t understand.

PARTICIPANT: I mean the medicine I take for high blood pressure in the GP there is.. I didn’t pay the payment is because of the CHAS. “CHAS” card.

INTERVIEWER: ok.

PARTICIPANT: here is… I need to pay or not?

INTERVIEWER: oh, I don’t know. But ah.. its more understand of making it more convenient for you. So you have the GP, you don’t pay for the medications.

PARTICIPANT: yay a.

**Reference 3 - 1.65% Coverage**

INTERVIEWER: every 2 months. Ok, and the blood test once a year?

PARTICIPANT: yes.

INTERVIEWER: yes, ok.

PARTICIPANT: last time I do it here.

INTERVIEWER: in the past you do it at IMH?

PARTICIPANT: ya.

INTERVIEWER: why did you change?

PARTICIPANT: because GP also can do. So.. also cheap la.

INTERVIEWER: oh, cheaper at GP. Is that also subsidised?

PARTICIPANT: ah, yes.

**Reference 4 - 1.86% Coverage**

INTERVIEWER: would you prefer to have a psychiatrist closer to you?

PARTICIPANT: yes. Yes. Provided the payment is the same.

INTERVIEWER: Provided the payment is the same. Yes. So very important to have equal cost. Ya? Does anything else factor into your decision to go to your GP or to come to IMH? Does any other.. Do you think of any other else? Other than cost when you choose to go to a GP or when you choose to see at IMH.

**Reference 5 - 0.96% Coverage**

INTERVIEWER: and if you were to get your hypertension medication here, you have to get too much at once. Because your… is that what you were saying?

PARTICIPANT: ya, too much at once. And another one thing is the cost la.

**Reference 6 - 1.14% Coverage**

INTERVIEWER: so do you have any other thoughts about why you prefer to see a psychiatrist versus a GP? For your mental illness? Is it just because of cost or is it because of anything else?

PARTICIPANT: cost

INTERVIEWER: cost

PARTICIPANT: cost and also near my..

**<Internals\\HDL Study-Service User_140210-0112> - § 2 references coded [1.77% Coverage]**

**Reference 1 - 0.79% Coverage**

PARTICIPANT: I mean.. sorry, I check-up at polyclinic. I work at GP at Eunos. Yah, my polyclinic is at tampines.So I live in tampines, so its quite nearby la.

**Reference 2 - 0.98% Coverage**

PARTICIPANT: so far no, I mean I’m ok with going to the polyclinic and also to IMH for treatment even though IMH is a bit far from my place. Ya, so far I’m ok with it la. With ..both schedule with me.

**<Internals\\HDL Study-Service User_140214-0118> - § 5 references coded [6.41% Coverage]**

**Reference 1 - 1.75% Coverage**

INTERVIEWER: at the polyclinics is not like that?

PARTICIPANT: yah, polyclinic they have to wait. But now they come up with a system whereby you can make appointment for certain more serious illness.

INTERVIEWER: (phone rings) sorry

PARTICIPANT: ok, never mind.

INTERVIEWER: so, no real trouble getting appointments at polyclinic.

PARTICIPANT: no, no real trouble there. Its fine, we can make appointment. They accept us to make appointment. But the problem is that the appointment line is difficult to get through. For IMH, the appointment line is better.

INTERVIEWER: oh, the telephone line.

PARTICIPANT: yah, yes.

**Reference 2 - 0.66% Coverage**

INTERVIEWER: and if we talk again about how often you come to IMH and the polyclinic. Does it bother you to have to come so often?

PARTICIPANT: at first it bothers me because of the transport charges, that we have to.. you know. Incur.

**Reference 3 - 1.63% Coverage**

But I always tell the DR I want to commit myself to this proper treatment. Complete treatment. So that when I get well, I can do what my next stage of life can do. If I don’t… If I do half way, that means my treatment is not complete I will like what you said. Relapse. And when I suffer relapse, nobody able to take care of me, cause I’m on my own. You see, I don’t have grandmothers, I don’t have you know grandfathers all these. Who can take care of me, I don’t. you see, I’m on my own. So I have to make sure that I am able to be independent as them, and stand up well fit. Yes.

**Reference 4 - 0.79% Coverage**

PARTICIPANT: I like about is because they help us with our financial problems also. Like we can get the medical officer to issue you a medical .. Medical certificate of sickness. They can help us on that. So we can go to the government and say that we need some help. That’s one thing.

**Reference 5 - 1.58% Coverage**

I:ok, I’m very glad that you can speak with us today. Hope you had… anything else that you want to tell us? Anything else that you think is..

PARTICIPANT: I think in the moment, just that. It’s just that for patients like us, we have.. we need financial support. Because when our families don’t give us financial support, we are at a loss. So we need the support, financially support, emotional support. And ah.. whatever they can help us. We are very happy to receive it. Yes. Because when they make problems for us. You know we can be hopeless anything. Yah, yes.

**<Internals\\HDL study-service user_151210-0147> - § 3 references coded [3.86% Coverage]**

**Reference 1 - 1.43% Coverage**

INTERVIEWER: no, ok. Do you think IMH is a suitable place for you to get your treatment for schizophrenia and your high cholesterol?

PARTICIPANT: yes.

INTERVIEWER: or would you prefer to see a specialist for both?

PARTICIPANT: no.

INTERVIEWER: no?

PARTICIPANT: no, financially I cannot support.

**Reference 2 - 1.14% Coverage**

INTERVIEWER: yah. And how did you feel about having to come to IMH for follow ups?

PARTICIPANT: it’s ok

INTERVIEWER: it’s ok?

PARTICIPANT: yah.

INTERVIEWER: But it’s difficult financially to go to 2 specialist.

PARTICIPANT: hmm, yes.

**Reference 3 - 1.29% Coverage**

INTERVIEWER: no, ok. What about your family. Does your family support you?

PARTICIPANT: yes

INTERVIEWER: yes, can we talk about that a bit?

PARTICIPANT: yah, they supporting financially.

INTERVIEWER: financially. Anywhere else?

PARTICIPANT: ah, one month about $150.

**<Internals\\SP 140130-0095> - § 1 reference coded [2.29% Coverage]**

**Reference 1 - 2.29% Coverage**

Now what about the group that does not come back, does not comply and we see them only when they become very ill? Does this group differ in terms of like characteristics compared to those that are willing to other than insight and…

Participant: Well absolutely they tend to beside the fact that they have less insight they tend…their conditions tends to be more severe in general in both… both, I think mostly for the psychiatric condition there is tiny bit of insight though they tend to be more severe they also tend to have poorer socio economic statuses that does tend to be something we see. Because…We now have such a robust tracking system that patients who are contactable, that means have a fixed abode, have family members we can be contact generally we can get them back; the ones who are most likely to be lost to follow-up are those that perhaps have… estranged with their family or no family, have no fixed abode and picked up by police when they are creating a scene…or if they are begging something like that and then they are brought in. There will systematic socioeconomic demographic characteristics as well as the severity of the illness I would speculate because I don’t evidence for this, they will probably be more severe, more likely to have schizophrenia, more likely to have poor social support, more likely to have a forensic record actually probably so anything else

**<Internals\\SP_140120-0083> - § 1 reference coded [0.87% Coverage]**

**Reference 1 - 0.87% Coverage**

And what role do you think social support (6:36) social support

Participant: Family members because erm because if they the family members they help them keep track of appointments, remind them to take medication and sort of things like that you see so sometimes it’s diff for them to remember multiple appointments yah
