# Supplementary material for: The Impact of Stakeholder Preferences on Service User Adherence to Treatments for Schizophrenia and Metabolic Comorbidities
Source: PLoS One. 2016 Nov 16;11(11):e0166171. doi: 10.1371/journal.pone.0166171 (PMC5112999; doi:10.1371/journal.pone.0166171)
Supplement: S1 File — This file contains the nodes used to construct the themes reported in the manuscript. Including advice to others; expertise; insight into illness; instructions; looking after kin; preferences; relapse; resistance to doctor’s orders; social factors; social support; stigma; therapeutic alliance; and uneasy about initiating treatment. (ZIP) [file pone.0166171.s001.zip › Qualitative data/Social Support.docx]

**Name:** Social Support

**<Internals\\HDL Study - service user HDL_151224-0139> - § 1 reference coded [0.33% Coverage]**

**Reference 1 - 0.33% Coverage**

And they don’t have to feel lonely at home, they can visit friends and talk to parents. Yah

**<Internals\\HDL study service users HDL_151209-0140> - § 7 references coded [9.45% Coverage]**

**Reference 1 - 1.62% Coverage**

And… the progression is quite good, since I… way back 10 years ago and now. I adjust myself around 8 or 9 like that, around 10. The very best is 10. Yah. Because I got to know my condition, and then a lot of support group I attend, you know. A lot of people I met with schizophrenia, bipolar, you know. Or depression. They came to me like, we sit around. Sometimes we chat. You know. You should do this, you should exercise. You should eat wisely and that. Besides, the Dr advise I got some of my friends la. Who are suffer from mental illness, we share together. we… just sit down you know. All these.

**References 2-3 - 1.68% Coverage**

INTERVIEWER: so you have a good social networks that also helps you.

PARTICIPANT: yes

INTERVIEWER: made up of people who also mental illness . how do you meet those people?

PARTICIPANT: every week, they have this .. like Saturday or Sunday, they have a meet up. You know. Just like in a beach, you know. Cycling, in the beach, or morning jogging or walk in the park you know.

INTERVIEWER: is it something like IMH or is it independent?

PARTICIPANT: its kind of not in IMH ah. It’s a social network outside. Like the SACS, SAMHS, we… we friends together then we plan outside, you know. Do activities together. You know

**Reference 4 - 0.84% Coverage**

Because I’m sure you understand you are not the only one who goes through this progression and if we understand how people like yourself experiences. We hopefully can help those who are now sort of developing the illness. And I’m sure in your circle of support, you must sort of tell sort of newly diagnosed people

**Reference 5 - 3.54% Coverage**

How do you think you changed to now sort of understand that is treatment that you sort of comply with. Answer, how did you think you changed? How did that happened?

PARTICIPANT: I changed because of social networks. Because you know before Julius. You met Julius Athanasius, I think Athanasius. Before him was Mr Harry Ng, eh… Harris Ng. I met him, outside la, nearby Pasir Ris. So I’ve been taking his advise sometimes, you know. Meet him, just for one hour. Because he suffering, think more than 40 over years. 50 over years. Schizophrenia you know. 40 over years. Right now, he’s off his medicine. So, kind of like mentoring thing la. When I’m free, or he’s free. Go anywhere sit down at coffeebean or starbucks. You know, have a coffee for one hour session. Just ask him what are your fears, what are your paranoia, what are your conditions and what.. what can you.. what can I gain from him. The knowledge, basically. He’s off his medications, he’s doing well you know. Doing tuition, you know. He’s married but don’t have children. Yah.

INTERVIEWER: ok. and that social support helped you understand.

PARTICIPANT: and social support some of my friends outside, SAMH , SACS, club heal, you know. All this, yah.

INTERVIEWER: so they help you sort of progress to understanding the illness.

PARTICIPANT: yes

**Reference 6 - 0.50% Coverage**

I slept, I take this medication, but I can help people change their life ah. Without this suicidal, without this loneliness, you know. Yah. Have this circle of friends, you know. Yah.

**Reference 7 - 1.25% Coverage**

INTERVIEWER: cause it’s sort of recovery. It’s the recovery of the patients. So is it that you meet people here that have more to teach you, to help with your recovery?

PARTICIPANT: I think base on association like SACS, Club Heal. You know. Over there, I can have this circle of friend who have this support system la. yah, if not.. not basically here la. Yah.

INTERVIEWER: so they are not those circle of supports are not from IMH

PARTICIPANT: no. not at all.

**<Internals\\HDL_CG151209-0142> - § 3 references coded [4.85% Coverage]**

**Reference 1 - 1.95% Coverage**

I tell you the CAL the team meetings, 20 hours about the mental problem people, all the caregiver went together, so I saw people all different all mental but different problem, so all are very different

PARTICIPANT2: there are other support for caregivers la

INTERVIEWER: yes , do you find those through IMH? or

PARTICIPANT2: through IMH, right?

PARTICIPANT: yes

PARTICIPANT2: I think IMH has quite comprehensive care, it depends on how willing the patient and caregiver goes forward to find take up you know and how much we can understand la , not everybody has that education level to understand certain tings, that is why many people will not join, it is beyond them and handling the daily requirements of the needs of the patient is already very taxing.

**Reference 2 - 1.98% Coverage**

PARTICIPANT2: that is why the fame club not bad , though it is run by the church, I think maybe it is also sponsored by government a little but, a voluntary kind of, it is quite good, but they can do more if it is a whole day, and even more funding, they can do more, because the few person running now they have the empathy, they really love those people, they are not nurses, not trained, but social worker, they have the heart, and that day I went to the family day, because of Chinese new year, so the client the patient asked the family member some of them came. I see a lot of them, 5019 [daughter] looks nicer than the others, not nice, but you can tell, but they are happy, they are happy because they are accepted there, because they are of the same group, they enjoy

**Reference 3 - 0.93% Coverage**

PARTICIPANT2: a place something like this, and I think these patients demands are not so high, but it is just that their symptoms, and these people are very understanding, if they don’t want they don’t force them. They teach us not to expect too much from the patients. Then these patients are happy. Family members are happy and I think the country is also happy.

**<Internals\\HDL_CG151210-0163> - § 1 reference coded [3.66% Coverage]**

**Reference 1 - 3.66% Coverage**

Because sometimes when I bring my mother out and then the way she walks, she takes time entering the lift, and then a lot of the people at the back, are upset, making those types of remarks … you know… I know her, she is walking like this because of her condition, but you need to be more patient for the public I mean. So yeah those are the things, patience understanding about the person’s health condition, and the support from family and the community, it really helps.

INTERVIEWER: do you feel that you have enough support from the community?

PARTICIPANT: for my family yes, for my family siblings father as well as both my father and mother side of the family, my uncles my aunts, they know my mother’s condition, so, yeah they do a bit of contribution financially, and then when they come for some gathering they will ask her “how are you have you been taking medicine” so there is that form of support, and they call her or she calls them to talk. So I, for my family I notice that kind of support, but for other families whereby they may have lost touch, or maybe just one son or one daughter, and do not have other siblings, I believe it is very taxing on that son to take care of someone with that condition as compared with someone who has, like I am quite grateful because I have two siblings to rely upon, and my uncles and aunties are all concerned, they know of my mom’s condition and they call her , she calls them back, so it is ok. It is, I have been receiving support.
