# Supplementary material for: The Impact of Stakeholder Preferences on Service User Adherence to Treatments for Schizophrenia and Metabolic Comorbidities
Source: PLoS One. 2016 Nov 16;11(11):e0166171. doi: 10.1371/journal.pone.0166171 (PMC5112999; doi:10.1371/journal.pone.0166171)
Supplement: S1 File — This file contains the nodes used to construct the themes reported in the manuscript. Including advice to others; expertise; insight into illness; instructions; looking after kin; preferences; relapse; resistance to doctor’s orders; social factors; social support; stigma; therapeutic alliance; and uneasy about initiating treatment. (ZIP) [file pone.0166171.s001.zip › Qualitative data/Stigma.docx]

**Name:** stigma

**<Internals\\HDL interview 2 20151111171452547 no audio> - § 5 references coded [31.28% Coverage]**

**Reference 1 - 7.45% Coverage**

At first noted that polyclinic never had a problem with treating him, but then noted that he had bad experience when a physician stigmatized him by pressuring him to say where he had a test done and felt she tried to get him to admit he had been to IMH, though he thinks that most staff do not know.

**References 2-5 - 23.83% Coverage**

Feels that society has undergone a maturation to accept medication and treated people with mental illness. But maybe less so for mental illness itself. Sees that Singapore has much western influence to inform its treatments for SMI, and that this has helped with stigma in the professional medical world. But still finds stigma in the work place:

Noted that when he was first diagnosed, he was naïve about stigma, and disclosed having a mental illness on job applications, but found that he was unsuccessful until he stopped disclosing and no longer tells employers that he has a mental illness and “fears being found out” . Which is upsetting since he can do what others do. Now has been in stable employment for a few years, approx. 5 .

Has not experienced stigma from medical professionals nurses of physicians at IMH, but has found administrative staff, especially the new ones to be “condescending”, “they talk to you like you are lower than them” .

**<Internals\\HDL interview 3 20160217171017621 no audio> - § 2 references coded [4.68% Coverage]**

**References 1-2 - 4.68% Coverage**

Feels that returning to work is hard because of stigma on job applications.

Believes there is stigma to going to IMH

**<Internals\\HDL study - service user HDL_151222-0138> - § 1 reference coded [1.25% Coverage]**

**Reference 1 - 1.25% Coverage**

INTERVIEWER: you were in school. Ok.do you think that coming to IMH is better so that there is less bullying here? Or where do you think that you are going to be bullied?

PARTICIPANT: I don’t know

INTERVIEWER: cause we called that stigma. People who are worried or afraid of people who have mental illness. Cause they don’t understand it.

PARTICIPANT: maybe its true la.

**<Internals\\HDL study -Service user HDL_140211-0114> - § 13 references coded [7.62% Coverage]**

**References 1-2 - 1.27% Coverage**

PARTICIPANT: also partly because of the hospital help lah. Well you know, initial I was very er… receptive to it. In fact, I was with my brother in law, my brother brought me here. i wasn’t very like.. where am I going? You know. It was like in the middle of the night you know? And then I realise later on I was in here actually. In hospital, mental hospital because somehow the connotation is still there you know? Even when you take taxi,driver is is ohh… you mean woodbridge. That mental hospital that kind of thing, you know? Even if they re-named the thing. But still that stigma is there. And then erm

**Reference 3 - 0.90% Coverage**

Then .. and then of course .. the so call secrecy. Nobody wants to.. more of my immediate family. Friends 1 or 2. I think you can count your fingers who knows what my condition is. I think for blood pressure is kind of ok to share, but for mental not really you know? So that’s the side issues whereby of course if I have a … say I wouldn’t want to have all these things. Want to have a normal life, perfect, healthy body, you know?

**References 4-5 - 0.60% Coverage**

But I guess so your practical message. But also depends on the.. on the individual lah. Some is can like connect to you more, the emotion level. You know, can see your family don’t like it, relative in front of you, stigma and so forth you know. So avoid all it, stay on course medications

**References 6-7 - 0.91% Coverage**

PARTICIPANT: yah, eventually also la. So that’s why my experience through employers sharing about the.. the stigma you know. Sometimes when you want to advice to a person, like an individual for those who are career minded who are here because of stress, work related. You can highlight these issues to them you know. About erm.. to be treated is better than having relapse you know. Secretly hiding away, you know. Kind of thing, yah.

**References 8-9 - 1.09% Coverage**

INTERVIEWER: no, I don’t.. I think because sometimes the… as I say the stigma the acceptance of it is still long way to go. You know. So and anyway to s hare you also see people whether they are really receptive to you sharing your condition. Some even minor things like blood pressure people might take issue even, you know. What more, serious things like mental. Cause what more is mental you can’t.. you can’t see it you know. It’s all hidden inside. You know. So, sometimes.. it’s a bit difficult. That kind of thing.

**References 10-11 - 2.32% Coverage**

PARTICIPANT: not.. I guess not really. I.. I don’t know whether you all do the stigma thing, you know. The acceptance. People even up to this day, even like modern also go to the.. belief that all these. Because for mental, as I say is not physical. You know, like leg cut off, kind of. Its more like actions, your behaviour kind of thing. People would said it is like.. for us. I think for Asians la, cholesterol it’s more like a. oh, people do you [curse you], you know, kind of thing. Especially the elderly. Oh ah, spirits and so forth you know. So sometimes, that is the natural reactions, you know. Bring her here, bring her there. Kind of thing. It’s only some those sane people say ok, this is not this, bring to hospital, ok. not any hospital. IMH hospital she should. I guess that person encounter similar. Cause I was brought to a mosque and you can hear screaming inside the room. Don’t know what the.. talking nonsense. Yah. I’m thinking, ok. its scary because of the episode, you know. Yah, and then. So this is the.. I mean. I guess you.. the to the… to.. the awareness to create the acceptance level.

**References 12-13 - 0.53% Coverage**

And.. if normal hospital, the acceptance level is there. Compared to here. Ultimately is the acceptance thing. The awareness. The stigma issue. With mental. So that’s the thing. If you can do something about it, which I really don’t know possible or not.

**<Internals\\HDL Study Service User HDL_151209-0145> - § 1 reference coded [1.16% Coverage]**

**Reference 1 - 1.16% Coverage**

INTERVIEWER: yah. Because sometimes we found that some people don’t like going outside of IMH because other people don’t understand what a mental illness is.

PARTICIPANT: Oh

INTERVIEWER: So they worry that they are not treated as well. Or they have problems like that.

PARTICIPANT: oh

INTERVIEWER: because people don’t understand mental illness.

PARTICIPANT: I don’t have such problem.

INTERVIEWER: ok, ok.

**<Internals\\HDL study service users HDL_151209-0140> - § 5 references coded [5.61% Coverage]**

**References 1-3 - 4.43% Coverage**

INTERVIEWER: ok. do you think you ever have trouble getting treatment here? Or any trouble in IMH? In terms of coming here or …

PARTICIPANT: no. don’t have any trouble.

INTERVIEWER: cause some people worry about the stigma of coming to..

PARTICIPANT: my worry.. I’m not worried la. But the 15 years I’ve been diagnosed as schizophrenia I mean. I always.. the perception of Singaporeans, of stigma. They can’t change like US or in UK, Europe or US place. you know if you have this diagnose, people can accept you. Down here in Singapore, it’s very hard. Seriously. Because I’m a malay also. But, inside the malay community, let’s say one or some of them knows that I’m diagnosed. I seek treatment in IMH, they would say that you are crazy. Laughs… that’s what Singapore stigma is la. They can’t change the way, you know… like in US or other countries, you know. It’s community.. accepting people. Yah. I had a hard time to tell myself that. In my family members also treat me as..”ohh, where you working now?” every Hari Raya will ask me. My family la, my siblings all that. Ask me where I working now and all these. Why you haven’t get married, you know, all this. It’s kind of like put me down. Laughs… pretty hard on me. Laughs…

INTERVIEWER: I understand, yah yah. It’s a very difficult thing for people to understand. And I think we take such a long time to learn. And if people are not willing to learn then it’s very difficult to change their minds. I’m glad that you are working with peers, it’s a very important thing that have people like you who do this sort of help with that. Cause it’s the step towards getting rid of the stigma.

**References 4-5 - 1.18% Coverage**

PARTICIPANT: I just don’t want because I found out that let’s say if I love that girl. Then after that she doesn’t know my diagnosis, you know. I can’t.. you know.. simply.. the girl says she found out that I was from IMH, you know. Diagnosed, maybe she would left me la. That’s why I can’t have a relationship, only friends la. Like normal friends.

2nd INTERVIEWER: so there’s still this stigma being attached that you feel la. Ok.

**<Internals\\HDL study service users HDL_151209-0149> - § 4 references coded [11.28% Coverage]**

**Reference 1 - 3.38% Coverage**

I: because some people experience problems when they go to the polyclinic, they do not feel comfortable, thy feel that there is stigma against the mental illness, and they worry about going there, and they only want to come here, or some people worry about coming to IMH because there is stigma about coming to IMH, do you feel that there is anything like that that plays into why you choose one place or the other?

P: hum… I think… coming here is more comfortable, but also , like my mum doesn’t like it when we come to IMH for too many times, like when I come here for vocational training she is a bit upset because I have to come here 3 times a week , so there is some stigma, that if you go to IMH a lot , people will have a lot to say but I don’t find anything at the polyclinic because I don’t think they know, but even if I were to tell them, I am ok with it, because I am sure that they will keep it confidential. Yeah.

**Reference 2 - 4.12% Coverage**

I: ok, so the days you have vocational training you come and go on your own, and so she doesn’t like it when you come too often?

P: yeah

I: so it is not because it is an inconvenience to her to have to bring you, but it is because of the stigma of going too often?

P: yes

I: just to be clear, I wasn’t too clear on that, thanks.

P: yeah

I: so can we talk more about what exactly she is expecting of that type of stigma? Is it because she is worried that your friends are going to think something or is she worried that the neighbors are going to think something? The family is going to think something? Where does she think or where do you think the stigma is going to come from?

P: I think from her family, and also our neighbors, yeah, I think like she didn’t tell her family, her sisters about my condition, only my immediate family knows, then I think I told one or two of my close friends, I think she just worries about like people judging us, because some people they don’t like people with mental illness, or they think bad things about them and they say bad things, and I think she just wants to protect me from that.

**Reference 3 - 1.82% Coverage**

I: and how does that make you feel?

P: I feel…I feel happy that she wants to protect me, but also I feel like… there will come a time that I will, that someone will be prejudice against me because of my mental illness and I have to deal with it, and what is important is not what they think but my mental health, that is the important thing, so I think that maybe if she just don’t care about what other people think and focus on my mental health, that is the important thing. It is important to me.

**Reference 4 - 1.96% Coverage**

I: that is very interesting I like that a lot. Can we talk more about that, what do you mean when you said that your mother didn’t want you to take the medication because she did not see you as mentally ill?

P: because I guess it is the stigma, she doesn’t want people to think that I am a crazy person, that I will stay in the ward, and she just and she was in denial, she didn’t want to believe that I was sick, and also it has to do with religious aspects, she just things that I am troubled because I did not go to church or something.

**<Internals\\HDL study_ service user HDL_151023_0040> - § 1 reference coded [5.41% Coverage]**

**Reference 1 - 5.41% Coverage**

do think that your life has changed since your diagnosis with schizophrenia?

PARTICIPANT: that’s a.. I still cannot accept it.

INTERVIEWER: it is still very difficult, yah.

PARTICIPANT: hmm?

INTERVIEWER: it is very difficult to accept

PARTICIPANT: yah, difficult to accept

INTERVIEWER: can you tell me a bit more about what is difficult. Why don’t..

PARTICIPANT: there is a stigma about this schizophrenia.. that’s the difficult part ah. People think you are mad.

INTERVIEWER: have you ever have trouble with Drs thinking that you are mad?

PARTICIPANT: no. Drs don’t treat me like I’m mad. They treat me with as if I have an illness. They try to cure the illness. That’s different ah. They show that kind of attitude. So ok la, it has happened to me. I just take the medicine. I’m already 58 years old. How long more am I going to live? So just accept it.

INTERVIEWER: where did you.. have you ever experienced stigma? Because people found out you have schizophrenia.

PARTICIPANT: yah, my friends know about it. Sometimes they make fun of me. They call me mad and all that. So I stayed away from them la.

**<Internals\\HDL Study_service user HDL_151023_0035> - § 2 references coded [3.24% Coverage]**

**References 1-2 - 3.24% Coverage**

INTERVIEWER: ok. ok, some people.. we actually found out that some people don’t want to come IMH for treatment because of stigma and stuffs like that.

PARTICIPANT: sorry?

INTERVIEWER: because of stigma.

PARTICIPANT: huh? Whats that?

INTERVIEWER: stigma is like.. like some people find IMH has a reputation for crazy people

PARTICIPANT: I don’t feel that way.

INTERVIEWER: yah, but some people do feel that way. So, for these people right. Is there anything that you like to… in your experience.. is there anything you like to tell them?

PARTICIPANT: because I don’t really know what is crazy.. maybe crazy is.. only you are crazy, you are normal. Maybe it is that way.

**<Internals\\HDL Study_service user HDL_151210-0137> - § 3 references coded [4.94% Coverage]**

**Reference 1 - 2.16% Coverage**

but was there a particular reason why you go to mount Elizabeth? Or who brought you there?

PARTICIPANT: my aunty.

INTERVIEWER: ok. but.. could you tell me more about that? As in why did she bring you there?

PARTICIPANT: because… my family members quite discrete at that time. They don’t like me to go IMH because last time IMH is very old and many people very complicated. So they decided me to see Dr through Mount Elizabeth’s private psychiatrist. Yah.

**References 2-3 - 2.78% Coverage**

INTERVIEWER: So.. when you go to Queenstown to see for your condition right, did you faced any issues, stigma or anything like that?

PARTICIPANT: no.

INTERVIEWER: no. How about when you are coming here for treatment? Did you faced any such..

PARTICIPANT: yes

INTERVIEWER: you did.

PARTICIPANT: yah

INTERVIEWER: from who? Can you tell me abit about that.

PARTICIPANT: ah… its like. Quite embarrassed.

INTERVIEWER: ok

PARTICIPANT: yah.

INTERVIEWER: but how about people around you?

PARTICIPANT: er.. some is friendly but some is.. er.. they curse and swear behind your back.

**<Internals\\HDL Study-Service User HDL_151203_0061> - § 2 references coded [1.19% Coverage]**

**References 1-2 - 1.19% Coverage**

Interviewer: Erm ok some people tell us that they find it difficult to come here for treatment because of stigma and stuff, do you have such …?

Participant: No

Interviewer: So you find it ok to come here, you are happy

Participant: Yah

**<Internals\\HDL study-service user HDL_151209-0152> - § 1 reference coded [3.16% Coverage]**

**Reference 1 - 3.16% Coverage**

Nobody want me. In Singapore, not enough, the market. So people outside want to come here, they also scared. Because some of the patient ah. Last time got people call me, I walk down there. then people call me.

INTERVIEWER: ah.. from the ward they called.

PARTICIPANT: hmm?

INTERVIEWER: from the ward, they call you?

PARTICIPANT: ah

INTERVIEWER: when you walk outside

PARTICIPANT: when I walk… above. Got people call me. Say come come.

INTERVIEWER: then what do you do?

PARTICIPANT: huh?

INTERVIEWER: then what do you do?

PARTICIPANT: I just ignore

INTERVIEWER: ignore, ok.

PARTICIPANT: they say come come. So, they feel that coming to IMH is not a safety place ah. Not a safety. They… The heart is pumping ah

INTERVIEWER: oh, you have

PARTICIPANT: fear fear fear. Fear whether if.. like not safety place ah. Fear, maybe the police also not at the bothered.. bothered to serve the… help the.. anything what happen. Like terrorist like that.

INTERVIEWER: so do you think the polyclinic is safer?

PARTICIPANT: polyclinic safer

INTERVIEWER: polyclinic safer, ok.

PARTICIPANT: because people name IMH as a… you know, lunatic

INTERVIEWER: hmm?

PARTICIPANT: L.U.

INTERVIEWER: Lunatic

PARTICIPANT: ah

INTERVIEWER: Lunatic, ok. yah.

PARTICIPANT: cause they got the bad reputation ah. Ah, so people scared la, scared and fear.

**<Internals\\HDL Study-Service User_140113-0128> - § 8 references coded [15.72% Coverage]**

**References 1-4 - 8.43% Coverage**

INTERVIEWER: and some people have reservations about coming IMH. Because it is hospital that has a history. Did that factor into your decisions?

PARTICIPANT: not really. Because I was having debts at the bank. Cost of like.. one and a half month of being at Mt E, cost to around $1000+ and I take the medications daily. I’m not able to cough up with the cost. So the cost is actually one big factor that I decide to come to IMH. But then.. my parents always come with me. As in right now my dad drove me here. They always come with me whenever I come here. Because I was thinking basically it Is the stigma of like.. what the mental hospital will have. Especially when I come over for consultations with Dr Leong. I was quite.. I won’t say I was scared or afraid. I was quite uncomfortable with the people all around me. As in the patients around me. Yah, sometimes. But. I told.. because Dr Leong actually advise me to go on the jobs club. And sometimes what courses what I can actually take part in IMH. And I told him that but my parents would have to come with me. And he was saying why my parents will have to come with me. I can come here myself alone. I just told him that it is the stigma I have. A slight stigma that I have and what my parents have towards the hospital. As in patients environment around. Yah.

INTERVIEWER: Can you tell me a bit more? Because I’m not.. I don’t know if I understand.

PARTICIPANT: as in, the patients.. they are very.. as in.. erm, how do I say?

INTERVIEWER: is that in acutely ill?

PARTICIPANT: hmm?

INTERVIEWER: is that they are acutely ill?

PARTICIPANT: yah. Sometimes it’s a bit uncomfortable. I know that wasn’t ... I have schizophrenia but I am … but it doesn’t.. If I wasn’t.. wasn’t having any episode rite? I.. If… Ok? I cannot find a correct word for it. As in… the patients would shout and then say things out of the blue that is not really happening. It really scares me. Yah, so my parents have the same thinking that I have. So, whenever I come here, they will have to follow me. As in they will have to drive me here and then come with me. As in. such as, as in just to protect me from the patients themselves. I told Dr Leong about it, and then he said that IMH is actually very safe. Yah, but I can’t get to change that stigma. About that kind of thinking, about the fellow patients here. Yah.

**Reference 5 - 3.38% Coverage**

INTERVIEWER: so you worry and your parents are worried about you coming alone.

PARTICIPANT: yah

INTERVIEWER: because of the risks of other patients?

PARTICIPANT: yah.

INTERVIEWER: ok. Do you know what sort of led this to develop? What contributed to this type of fear?

PARTICIPANT: as in the actions of those patients itself?

INTERVIEWER: yah, sort of. Cause we are curious is to know if people don’t want to come IMH because they have this fear. And we wanna know where this fear is coming from. Because it is quite safe and they are very few incidences with patients. Sort of wondering if history, and people just remember that it was a mental hospital. An asylum.

PARTICIPANT: its not really the history. It’s the actions of those patients that put me in fear.

INTERVIEWER: so you are in clinic B?

PARTICIPANT: yes

INTERVIEWER: so when somebody talk to themselves or acting strangely makes you uncomfortable.

PARTICIPANT: yah.

**References 6-7 - 0.91% Coverage**

INTERVIEWER: what do you think would make you safer?

PARTICIPANT: that’s a difficult question… I don’t know.

INTERVIEWER: yah… cause we want to see if it is possible to sort of change that stigma. No ideas of how..? so your father dropped you here?

**Reference 8 - 3.00% Coverage**

INTERVIEWER: no. when you went Mt Elizabeth, did you also have to wait in waiting rooms with people? Like clinic B?

PARTICIPANT: no. no. as in the clinic in Mt E they don’t have such patients that are like.. what you do in clinic B. yah. As in they act normal. Yah, then there is no strange actions or strange things coming out from them. Over the patients at Mt E. So, I was quite apprehensive when I come to IMH clinic B. And I see things like that happening around me. I get uncomfortable. Hmm. Cause this is exactly opposite of what I experienced back in Mt E.

INTERVIEWER: And has this type of fear sort of change? Does it always the same? Or?

PARTICIPANT: it has lessen. Over the times I had, I here in clinic B. It has lessen. But my parents were still worried, so whenever I came here, I come here with my dad will follow me. Yah

**<Internals\\HDL Study-Service User_140209-0108> - § 2 references coded [7.29% Coverage]**

**Reference 1 - 5.07% Coverage**

PARTICIPANT: they .. Dr don’t ask much. They just.., then the last one., the last one they change Dr each time. They won’t same Dr. maybe the same system like here. So they .. the female Dr ask me did you go follow up with your IMH.. actually she say somewhere like.. because I go with my friend, my friend follow me in. I don’t want to let her hear that I go to a psychiatrist at IMH. I don’t. Because usually they find me very normal, only my close. My close friend all don’t know that I got treatment for IMH only like my family. My immediate family they know this. Then I don’t want my friend to hear. She very helpful, she follow me in the Dr. ask the Dr about the milk powder, the brand. She got diabetes also. Is it too sweet to take. Then the doctor ask me, you go to Aljunied or where? Which Dr? I said no, I just say no. I din correct her that I go IMH. Cos I dun want to let my friend hear, that this is personal. Confidential. So I don’t let her hear, so I din correct back the Dr. So I kept quiet only. Then my friend came out and say you see psychiatrist? For your treatment? The Dr ask me got go for treatment? I said yah, sometimes, got. She say where? I say private, I dun tell her I come to IMH to get. Wait they may regard me as not normal. If they find out. I’m not a normal person. But I’m very well. I’m very well.

**Reference 2 - 2.22% Coverage**

INTERVIEWER: ok. What about why you are worried that your friends find out that you are come to IMH.

PARTICIPANT: oh, because… I don’t want tell them. Maybe they think… but.. I find a friend in need is a friend obvious indeed right? That they help, I help them. They want company, I accompany them to like go to certain places to the children block safe, safe for keeping their jewelry, the cisco there. I mean they trust me, maybe they found out that I.. I go to IMH, I’m not a normal person.

INTERVIEWER: so you worry that they may not trust you as much.

PARTICIPANT: yah yah.

**<Internals\\HDL Study-Service User_140210-0112> - § 9 references coded [11.79% Coverage]**

**References 1-2 - 1.98% Coverage**

INTERVIEWER: so, you are ok with coming here for treatment? Or ?

PARTICIPANT: ah..

INTERVIEWER: at first how was it?

PARTICIPANT: At first I don’t because of the stigma. Having.. being a mental patient. Yah, and being.. you know go to IMH is like.. something embarrassing la. I would say. Ya. Like, if people know about it, they would either laugh or they will like shun you away… that kind of thing.

**References 3-7 - 8.50% Coverage**

INTERVIEWER: no? because when we began our conversation, you mentioned that at first you were worried coming to IMH for

PARTICIPANT: ya.

INTERVIEWER: stigma reason and something like that.

PARTICIPANT: ya., that is one of mine .. ah, what you called it? Reasons ah, to come to IMH. I mean I’m afraid of the stigma that people will have for those people with IMH. Because they.. people always think that, oh. People who seek treatment in IMH are usually those mad people and .. they don’t get well. That’s why they have to go regularly . regularly for check ups and all that such things. Ya.. and even maybe. Even if you go out to work, if you declare that you are from IMH, they might not take you in., ya, that’s the stigma I have for going IMH. But then, I have no choice but to go. Cause I don’t want to have any future attacks. Ya.

INTERVIEWER: so do you think that the experiences of coming here have changed your belief with the stigma? Or do you still think that it’s a place for people who are not.. not well. Cause you are well, you haven’t had a relapse and all in a while. You managed to take medications but you functioned well, yes?

PARTICIPANT: I’m slowly trying to get away from the stigama. Don’t think too much about it and just go on with the treatment as per normal. Like, as though I’m going to the polyclinic to go for my.. my medical conditions check up. Ya. Ya, I mean so as to prevent myself from over thinking that.. ah.. this place is .. a place where I have to be.. I mean I have to be seeking treatment from the doctor for the next how many years. So, I can’t say that I’m totally well because I’m still under treatment unless OI am not taking any treatment for the past 1 year and maybe. Ya.

**References 8-9 - 1.31% Coverage**

INTERVIEWER: so.. do you think that there are any delay in you seeking treatment from IMH cause you are worried about the stigma or .. sort of .. you are brought here. Or when you came when it was necessary and then you have been able to follow up successfully with..

**<Internals\\HDL Study-Service User_140214-0118> - § 1 reference coded [1.91% Coverage]**

**Reference 1 - 1.91% Coverage**

Schizophrenia is a very broad name for mental illness. It is a very broad term. In the past when you have schizophrenia, you will be thrown, you know into some where. People don’t want to see you, you know. Now in the modern society, its different. People are seeking treatment and people are able to accept schizophrenia, they able to understand schizophrenia is a illness which can be treated. In the past, people maybe think that schizophrenia cannot be treated. So the patient, schizophrenia patient thrown into some where. In the forest or you know in the.. other countries the more traditional country, throw into the forest and then you know. Nobody will go near them like this.

**<Internals\\HDL_CG 151023_0036> - § 4 references coded [16.66% Coverage]**

**Reference 1 - 3.08% Coverage**

PARTICIPANT: I feel it is all about medication, other than medication I don’t feel that the doctor did, like advise him, ask him “how is your life” like, how to say, because he doesn’t even aware of his conditions very deeply, I could say. He just think I need to take medication, and he don’t mind to come here, because “if a normal person wouldn’t mind that this is IMH I want to be normal, I want to get well soon” I hope that he could, the doctor could help him more in mentality ,telling him he needs to get well and stuff.

**Reference 2 - 1.21% Coverage**

PARTICIPANT: uhm if me, I would prefer elsewhere because I might be like you said, those people who hear that IMH is a bit like you know, yeah, so I prefer elsewhere actually if possible, like at polyclinic.

**Reference 3 - 7.90% Coverage**

so you said that a normal person would not actually come here for treatment?

PARTICIPANT: no, they might, some people might have that opinion, so some of them might feel it is ok to come here, but to me, if me, I don’t think I would like to be here because it is IMH and how my relatives see, and friends and stuff, so I hope as normal as possible if possible

INTERVIEWER: as normal as possible?

PARTICIPANT: like polyclinic is ok, and someplace that is nearby the house. It is much convenient also.

INTERVIEWER: ok. Besides the stigma, right,

PARTICIPANT: sorry?

INTERVIEWER: stigma?

PARTICIPANT: what is that again?

INTERVIEWER: stigma is perceiving that IMH is for crazy people.

PARTICIPANT: ok

INTERVIEWER: so besides that , is there any other reason why you would not actually want your relative or someone close to you to seek treatment here?

PARTICIPANT: because I hope to see them normal, frankly speaking. I just hope for them to get well soon. , I don’t really like coming here too, frankly speaking , but I have no choice because I need to know his condition. So at times I’ll come here once in a while with him to see how is it going, and I will ask the doctor because I just realize the doctor doesn’t even tell much, just every time, “normal” kind of checkup medication, that’s it. How is he going to get well in this concept? Yeah

**Reference 4 - 4.47% Coverage**

INTERVIEWER: if possible you wouldn’t want someone who is close to you to receive treatment right? But you have not actually tried going elsewhere to seek treatment, right?

PARTICIPANT: we haven’t tried other place because this is the major, it is quite a big issue for him, like if without medication I am scared he may relapse, so I dare not try any other , unless the doctor here feels that he is ok to refer back to polyclinic and to try for a few months, that way then, I’ll just follow because I am very scared that he will relapse. Yeah. Actually quite sure it is [inaudible] for us

INTERVIEWER: in what way?

PARTICIPANT: mentally like hope that he is well, so afraid that at any time he will suddenly relapse or collapse, and yeah it will be very stressful.

**<Internals\\HDL_CG 20151105 notes, no audio> - § 1 reference coded [5.94% Coverage]**

**Reference 1 - 5.94% Coverage**

physicians.

Caregiver participant aware of stigma, the public still has low understanding of SMI, still had a “fear factor”

**<Internals\\HDL_CG 20160426 notes, no audio> - § 1 reference coded [7.70% Coverage]**

**Reference 1 - 7.70% Coverage**

Has not experienced any major problems while getting treatment at IMH. Not particularly worried about stigma as she is quite used to seeing patients when she goes too other hospitals (referring to patients without mental illness) so does not regard them as dangerous and is not uncomfortable around them.

**<Internals\\HDL_CG151209-0142> - § 3 references coded [4.31% Coverage]**

**Reference 1 - 1.16% Coverage**

PARTICIPANT2: I think she just feels that she wants to be normal la, but she is unable to, so somehow sometimes, that is why , she shared with the doctor just now that , sometimes she is just talking to somebody, but when we ask her she will say no, nothing she is not talking, then somehow she gave a new perspective to what she is talking about, she told the doctor she is talking about how to improve herself, talking to herself about improving herself

**Reference 2 - 1.98% Coverage**

PARTICIPANT2: that is why the fame club not bad , though it is run by the church, I think maybe it is also sponsored by government a little but, a voluntary kind of, it is quite good, but they can do more if it is a whole day, and even more funding, they can do more, because the few person running now they have the empathy, they really love those people, they are not nurses, not trained, but social worker, they have the heart, and that day I went to the family day, because of Chinese new year, so the client the patient asked the family member some of them came. I see a lot of them, 5019 [daughter] looks nicer than the others, not nice, but you can tell, but they are happy, they are happy because they are accepted there, because they are of the same group, they enjoy

**Reference 3 - 1.17% Coverage**

these people also like social, some of them may be bother, [incomprehensive 5304] most people like this, normalized. [talks about the stigma of the local culture toward these patients]

INTERVIEWER: yeah that is maybe where the US is a bit better, normalizing the types of behaviors, as here it is still a bit stigmatized

PARTICIPANT2: yea stigma

PARTICIPANT: they still don’t learn, the customs officer in US are better

PARTICIPANT2: moving towards that.

**<Internals\\HDL_CG151209-0158> - § 2 references coded [4.06% Coverage]**

**Reference 1 - 0.23% Coverage**

IMH because they worry about stigma, that it would give a

**Reference 2 - 3.83% Coverage**

Does he have to go to a polyclinic on a regular basis?

PARTICIPANT: I tell you , before he was referred to polyclinic, but after that he make problem, then start to come back to here [IMH] . Before he was here then they refer to polyclinic, and then for a few years, ok over there. But then the problem start and we rushed here and then it becomes a permanent appointment with the IMH doctor, but there is no difference actually.

INTERVIEWER: there is no difference actually?

PARTICIPANT: I mean no difference, over there they only give him medicine, but here, after they make a problem, they decide to make injection, that is the difference, over there they only talk talk talk, and said ok, but after he make problem, then I rushed here, then they started giving him injections, and I found him more obedient, I mean no more problem, he doesn’t create trouble with the neighbor. Personally he is very obedient since his injections.

**<Internals\\SP 140130-0095> - § 2 references coded [1.84% Coverage]**

**Reference 1 - 0.40% Coverage**

the polyclinic itself may push back abit if you’re a patient with...”oh, you’re a imh patient” then oh, they want these kind of patients, there is a strong stigma there that they will disturb the other patients some is valid and some not so.

**Reference 2 - 1.44% Coverage**

So in certain cases where insight might be not the best that might take some persuading on the part of the physician to sort of explain why these treatments are necessary and to the person’s benefit.

Participant: That’s true.

Interviewer: And if the person comes from imh, the gp may not be as interested in making that effort to sort of push that

Participant: Possibly yes, certainly private gp the last thing they need is “oh god, why am I attracting patients with schizophrenia in this ward?” and they will sit there and look funny, they will scare the patients away, it’s a honest, it’s a real pragmatic issue, they will all be in the waiting area, the patient’s got…he looks funny, he’s odd, he’s weird, his mouth is moving, twitching in a funny way, let’s not go there, let’s go to the other gp, it’s a real concern, the polyclinic as well are concerned about this

**<Internals\\SP 140131-0096> - § 3 references coded [3.64% Coverage]**

**References 1-2 - 1.82% Coverage**

yes, I think there is this bias amongst, out there in the medical community about patients with mental illness, they sometimes think they make up their symptoms, their physical symptoms especially, and just because they have a mental illness, they think their physical illness is not as important, so I think, I am concerned that some patients get seen in the community, their physical illnesses are not take seriously enough. So that’ the danger of missing something important

**Reference 3 - 1.82% Coverage**

they are seen as a mental illness, unfortunately it still happens that they have stigma. I think among the primary care physicians, they may not know that antipsychotics may be related to metabolic syndrome, they need the awareness as well. That this is a risk factor. Yeah I have a feeling they may not be up to date in that aspect, so that may be a barrier to them receiving the proper care. Investigation and care in regards to HYPERTENSION, DIABETES, AND HYPERLIPIDEMIA

**<Internals\\SP_140116-0079> - § 4 references coded [5.04% Coverage]**

**References 1-2 - 1.11% Coverage**

**I think some of them just their comfort level they are used to coming over here but as polyclinic is something especially erm those who have chronic illnesses like they don’t feel comfortable in an unfamilaiar place. It could be that they have faced stigma in going to these places so this is like more familiar… they don’t feel, they feel comfortable coming over here.**

**References 3-4 - 3.93% Coverage**

**Interviewer: Do you think that there are any policies at IMH or policies in polyclinics that might in part be responsible for the stigma or any sort of negative experiences that they have or here for er less than optimal care for those HDL metabolic syndrome?**

**PARTICIPANT: Hmm…I think over here I think it’s basically that we are just recently becoming aware and then I think , well not really aware, but awareness of the need to treat and perhaps integrate our physical and medical care together at some…for some patients erm…polyclinic there is some… there is… because I have heard it’s not just for management of HDL but I’ve heard that because we’ve tried for many years to get our patients treated in polyclinics for a follow-up, and polyclinics…no we won’t be able to manage we don’t see psychiatric cases erm there’s a di…I think again is it stigma? is it lack of awareness? Is there some fear of the unknown? things like that**

**Interviewer: So it’s been repeated that they’re not willing to do that type of follow-up**

**PARTICIPANT: Yes… and then again I was talking to the doctor, she was saying that as it is they find it difficult for patients to…like their normal patients to follow-up with the treatment plan for chronic conditions, so with people with mental illness it becomes even more challenging**

**<Internals\\SP_140120-0082> - § 7 references coded [10.53% Coverage]**

**Reference 1 - 1.77% Coverage**

people don’t… that the people in the community, the general practitioners and all – they (25:22) seem to brush aside our patients when they do have medical complaint. So they may go there for headache, for example, but the family doctor will then – might just say that, “Oh, you’re a mental patient, maybe you should just get the help from the IMH.” (25:42) So I’m not sure how much of that is still happening now, but there’s always been like one reason we always cite like why our patients are not getting optimal medical care. Because I think no one – because of their mental health conditions – treat them very seriously.

**References 2-3 - 1.72% Coverage**

If I were to have to choose like, hospital…you know, because now for a fact is that our family doctor they don’t really manage mental health conditions. If they were to have to choose a hospital, I’m guessing it won’t be IMH. (29:23) I think the stigma is an issue, it’s not exactly convenient geographically speaking. And also think about if they were ever to need an admission, I know this place, I know the wards; would I want someone I know - if they were to need an admission – would I want them admitted here? I guess not. So probably I would advise that they get the help from a general hospital first…

**References 4-5 - 3.59% Coverage**

What about IMH – I understand that it has a past, a very strong past in the local psyche, but what about it do you think continues the idea of stigma, or continues the sort of, beliefs in the community? (30:11)

PARTICIPANT: I think it’s already an advantage here, because the press don’t really talk about it very much. But we do know of cases, where you know, somebody kills somebody else, then in the news, “Oh this person has got a past psychiatric illness.” So I think that’s even worse. But here although we don’t talk about it much, I think mental illness although it’s the illness of the mind, but you know in the behaviour you can still, I mean for some patients, it’s still something that…it’s observable. You know something is not quite right with the person. Don’t need mental health training in that…so if that person is not normal, I think it’s only natural for (31:00) the rest to then say that, “Hey, something is not quite right.” And that’s maybe an area where the stigma comes from. I mean we had a intern with our team recently, who talked about – who did a thesis about stigma having a survival benefit to the entire species. So I thought I mean, it’s not quite right to think about it that way, but maybe there’s some truth to it as well. (31:33)

**Reference 6 - 0.17% Coverage**

call it a discrimination or stigma. Now we’ve spoken quite a

**Reference 7 - 3.28% Coverage**

I think in Singapore it’s still about the volume of patients that you see that seems to matter, unfortunately. So if you’ve got the person who sees you for half an hour, you could have used that time to see another five patients and make five times as much money. We can’t expect family doctors to change the way of thinking. I mean you look at polyclinics – they are usually booked full. (36:36) How can they spare the time to, so much time spend with a particular patient just talk about their family issue? It’s not realistic either. So I think in the private practice sector, it’s not realistic because the GPs – most of them have got a waiting list – waiting time…and I don’t think they would be very keen to spend the extra time. They could use that time to just manage a couple of the more easy to treat, their medical conditions where they could earn more money. But in the polyclinic setting, they are – it’s already a very packed clinic, and you can’t expect the polyclinic doctor to spend extra time either. So still I think at the end of the day, you know, for it to work, maybe then it’ll still be a psychiatrist, but in the regional hospital.

**<Internals\\SP_140120-0083> - § 1 reference coded [1.22% Coverage]**

**Reference 1 - 1.22% Coverage**

Not really right now I mean maybe for other conditions but for schizophrenia very few and the past few times like I told you there were some gps who were quite willing to provide care but yah I remember that 1 gp who fed back after… after the problematic experience with this patient especially when the patient relapse and will just sleep on the floor of her clinic and I think she felt it was not possible for her to continue providing the care yah

**<Internals\\SP_140123-0081> - § 4 references coded [3.11% Coverage]**

**References 1-3 - 2.05% Coverage**

If I’m wearing the shoes of a…a…a patient or caregiver definitely I think I want a service that is convenient, that impose less hassle for me so er there’s still a strong degree of stigma in Singapore in fact, this morning the paper, the today paper, the free paper if they’re coming to imh in fact I should…it says something about affect the dignity

Interviewer: Affect what?

Participant: Dignity of the patient.

Interviewer: Dignity of the patient

Participant: Yah, not only stigma but dignity of patient (43:05) coming to imh

Interviewer: So the dignity of somebody coming to imh is compromised

Participant: Yes yes

Interviewer: Wow.

Participant: The paper here. I was reading it earlier on and I caught...yah. The last paragraph, (43:28) you can have this, the last paragraph. I read

Interviewer: Wow. I would add that patients and family members would choose the institute of mental health as a last resort because of the stigma and lost of dignity involved. so this is in the paper?

Participant: Today…the today paper

**Reference 4 - 1.07% Coverage**

Yah and again we hope if for example I’m a paid (?49:03) caregiver i hope the neighbors everyone will not stigmatize my family members seeing a psychiatrist but I think we do know that ‘this is not in my backyard syndrome’ is not something only available in Singapore, everywhere you know that you do not want to associate with people who are taking drugs as in whether illegal or legal, don’t want to associate with people who are deemed to be violent so I think er er educating the community is something that is a multi-pronged approach

**<Internals\\SP_140123-0084> - § 10 references coded [12.63% Coverage]**

**References 1-2 - 1.02% Coverage**

Erm…Challenges…I guess main challenges I would say is one… a certain proportion of them engaging them in treatment and er that’s related to either not not…having poor insight or the stigma issues ah…and secondly, in terms I think in epip now we have a lot of focus on physical health erm…and that being one of the reason why a lot young people don’t want treatment because of the physical comorbidities and er…so that’s another challenge we are facing right now.

**References 3-4 - 0.72% Coverage**

I wouldn’t say for everyone but I think er I wouldn’t know numbers erm but I would say certainly from having worked in epip at least I would say a quarter if not more would prefer to be followed up elsewhere er because there is a stigma issue er associated with imh so I do get patients requesting to be followed up elsewhere

**References 5-6 - 6.48% Coverage**

Would it sort of be a very similar explanation for the reason that some people prefer to be treated in the polyclinic versus those that prefer to be treated here?…have you experienced people who rather say “I’ll stick with…I’ll rather be seeing here…rather it’s…

Participant: No I don’t think that makes a difference er because then we are talking about stigma and I don’t think that makes a difference erm…in terms of whether they go to a polyclinic or gp for their medical comorbidities because either stigma is an issue and they just want follow-up elsewhere and they are comfortable with that or the issue is that if they’re comfortable with coming to imh it’s a matter of convenience meaning i… I’m working, I can’t do monday to friday therefore what are the other options for doing a blood test but they are comfortable in terms of going to see a polyclinic doctor so I don’t think the stigma issues per say comes… so if the stigma is there for the psychiatric illness, er…it’s it’s just purely that they don’t ask for follow-up for psychiatric issues at a polyclinic or gp generally because er we do have the gp partnership program where we transfer patients after a few years of care to the gps er…in the program that I find a lot of patients are okay with coming back here if the stigma issue isn’t er… if the stigma isn’t an issue for them so they don’t necessarily prefer to go to polyclinic or gp

Interviewer: Do you have a sense of what might be contributing to that sort of stigma experience or that sort of er…behavior that results from being… from perceiving stigma?

Participant: Erm…we had I think when we first started in epip before I joined epip I think they had done a survey and erm… so generally I think patients said one, imh has had a history from its woodbridge days, so it’s just a very long history of being associated as as you know…people who are crazy come here so therefore that’s still kind of has lingered on erm..I do think it’s improved because in epip we did a lot of er reducing stigma increasing awareness for psychosis outreach yah programs erm…so I do think it’s one, tied in with the spiritual belief system which is quite strong in asia and then it’s tied to the history that imh has in terms so it’s not really about…it’s about coming to a place and being seen by other people who perceive, who will then judge this person to have a mental illness, or might er so it’s a lot of losing face which again I think it’s a very Asian concept so they tell me “oh what if I come here and my friend sees me or I know my friend sees a psychiatrist here but if I get seen then everyone at school will know or at work they might know erm…or if my mc will say imh I mean there are ways around it but er if my mc says imh my employer is going to think I have a serious mental illness versus just maybe depression or that I’m already stable now. Erm…So there’s a lot of concerns attached to that.

**References 7-8 - 2.63% Coverage**

Do you think that if they have experiences that perpetuate the stigma cause these…these sort of experiences we’ve spoken about now are perceptions right?…they fear the and the history of imh is sort of something that lives on in the community psyche, but do you think the experiences that people have sort of on personal level or er… or interpersonal level explain why the stigma perpetuate?

Participant: Oh, Absolutely, I think NS, guys serving national service who when word…they have told me when word has gotten out that they’ve been to imh to see a psychiatrist...word gets out… and they have been bullied erm…or watched more closely, and every unusual maybe if they just have you know… display abit of agitation then immediately ppl are teasing them and…and…or catastrophizing the behavior…so yes… and in schools as well so for young people especially I think at least with work there is some degree of they may not know unless they inform their supervisor or employer er but in terms of school settings, NS especially they’re handing in mcs from imh then word does seem to sometimes get out and …so yes, it is not just based on their perception they do seem to have experiences…

**References 9-10 - 1.77% Coverage**

We don’t do too badly because our groups we run for epip patients only er we have something called club epip where we run groups erm depending on the group it doesn’t do too badly, like sports group isn’t so bad, yoga group may might be less well attended but I do think erm particularly two reasons why patients may…a lot of patients… some patients they don’t want to come to imh. One is the distance,patients who live in the west, south find it too far to travel, and secondly as the other part is the stigma who are already turning down all services because they would prefer to come here you know much lesser than they need to so…yah. We have tried running a group er at cwc, Queenstown so our occupational therapist tried that er but again we couldn’t justify because the numbers were pretty small

**<Internals\\SP_140123-0085> - § 4 references coded [2.14% Coverage]**

**References 1-2 - 1.42% Coverage**

And do you think those (20:02) people would prefer to be seen at the satellite clinics?

PARTICIPANT: Oh you’re talking about the stigma ? Yeah some patients – yeah they do think that they don’t want to be seen in IMH, cos you know of the label. It’s a mental health institute and they prefer to be seen in Queenstown or Geylang and they are – that’s why we have such clinics in the first place.

INTERVIEWER: Okay. And do you think the same type of issue of stigma might – I mean the people would rather be seen in the polyclinics?

PARTICIPANT: Yeah.

**References 3-4 - 0.71% Coverage**

social status…yeah of course, by large the higher social status you are, you are a higher social, like family background, of course you are worried a lot about the stigma, and then you prefer to be seen not only in CWC, but maybe some other private psychiatrist would see…yeah.

**<Internals\\SP_140125-0086> - § 8 references coded [6.97% Coverage]**

**Reference 1 - 1.42% Coverage**

a lot of times I think that this is a incidental like a patient seeing a GP for diabetes for example or the polyclinic they find out that er how come your blood sugar is always not under control? and they realize then oh you are on olanzapine or something like that then it is incidental but to us GPs to deliberately ask the patient all of them whether they are seeing this this this probably not and patients may not even be willing to say to the GP that they are actually seeing IMH I mean I’m using the example of olanzapine but there’s mirtazapine and other drugs that cause similar problems yah there’s still a stigma I think

**References 2-3 - 1.26% Coverage**

Ok so there’s a whole premise of the community health thing that we try to make it convenient for patients to come forward to get help, some of the few things will be accessibility, we try to have clinics in the community where patients can get both psychiatric care and medical care we need to reduce stigma, then coming forward is not a problem … for them I think education is important, motivating them for treatment is important so these things all need to be tied together it’s not something that one institution can do it by itself it is this whole community

**Reference 4 - 1.78% Coverage**

I think psychiatric patients are stigmatized against by other doctors as well if you come in and they know you are a IMH patient they may not take you so seriously or they would assume that you are not going to have poor…you are not going to have good care anyway I will just ok lah I will give you some medication, I will give you an appointment in 6months time, you come back and see me, whereas if the patient was someone who is educated, well-read high likelihood of complaining against you if you don’t give them good care maybe they will tcu 1 month to review medication and things like that I think this is unfortunately that is the case I think this kind of disparate treatment of patients exists everywhere either by condition, by skin color all these reasons but it happens everywhere

**References 5-6 - 1.70% Coverage**

Again it’s how we have been taught we are being taught that one way to reduce suicide rate is to treat the mental illness and it is correct if it is correct that by treating the mental illness we are reducing suicide risk however er most people don’t come forward there’s a stigma and erm and treating a mental illness probably only reduces a small part of it er I have a separate suicide prevention talk that I am going to give to the trainees, it’s about looking at it from a whole systems approach so whose job is it to reduce suicide I suspect again I am generalizing that if you ask this to other agencies people are going to say that this is a health problem but suicide is not a health problem suicide is a problem is everyone’s problem society’s problem

**References 7-8 - 0.81% Coverage**

And they are human they are expected to have at least the same amount of cancers in a normal population but focusing on that is a very low priority

Participant: Yah so again I don’t know whether this is again part of stigma that well it’s ok you know they already have schizophrenia they are so poor thing maybe it is alright it’s life you know, god is fair

**<Internals\\SP_140125-0087> - § 3 references coded [3.27% Coverage]**

**Reference 1 - 0.85% Coverage**

various reasons, firstly they are very comfortable with coming to IMH, they don’t want to have to see a doctor in IMH and at the same time go and see a doctor elsewhere, it is more troublesome for them, that is the first thing.

**References 2-3 - 2.43% Coverage**

INTERVIEWER: any sort of barriers?

PARTICIPANT: um hum barriers in patients receiving care from IMH or under …

INTERVIEWER: for example in IMH it may be stigma or something like that.

PARTICIPANT: oh ok, I see what you mean. Uhm. I guess to some extent if we do have patients who are cognitively impaired, like for example chronic schizophrenic patients, sometimes when they see other physicians they may have difficulty expressing themselves, and so sometimes their complaints are less , not taken as seriously, like non psychiatric patients. 1121 other barriers… I guess financial barriers, yeah like I was saying the medifund is not translated.

**<Internals\\SP_140202-0098> - § 4 references coded [5.69% Coverage]**

**References 1-2 - 4.23% Coverage**

Well I think those that er voice concerns over being seen at imh by and large personally I do feel that there is a huge stigma issue that comes across lah because I mean if you are being seen in a mental institution, psychiatric hospital I mean some of them are afraid that it would impact upon their jobs the way people see them so it is not so much that they are unhappy with the service you provide but rather there’s is a sense of er I mean they wonder whether or not this could impact subsequently on their livelihoods so by and large those people who do prefer to see us have come accepting that you know this is a condition that you need to take seriously that they are currently receiving good care with and they feel that you know going to a polyclinic may not be that helpful because there you they may not be given the time you know in the consult and also I guess if they are going to see the polyclinic they rather see the polyclinic is also probably because of their other medical conditional that they are already seeing for and they don’t really want to you know they are already on maintenance psychiatric medication and they don’t need any adjustments in the dose so it will be the ease of time for them if they go to see polyclinic doctor alone so i think er I think it’s moving… things are progressing in the sense that the stigma will be there but er it’s I mean I understand correctly it’s actually coming down because there various efforts to promote mental health awareness I mean if something is so common as to affect one in 10 people for a minor to a severe mental illness then…then it is necessary to get across the idea that we are some an institution that promotes mental health rather than just treating mental illnesses

**References 3-4 - 1.46% Coverage**

the other barrier is probably that of as mentioned previously the stigma associated whilst we can tell ourselves that you know we are trying our best to outreach to people you know to make people aware that mental health issues are you know quite common and it is nothing to be embarrassed about but stigma is something that is always a work in progress so it will never be off the shelf completely so er one of which being in the community might you know help to relive that stigma and er that is probably a barrier for patients who want to see us but feel that it might influence them in some way yah

**<Internals\\SP_140203-0100> - § 7 references coded [6.83% Coverage]**

**References 1-2 - 3.18% Coverage**

I think the main thing is how you sort of how the services are designed, traditionally they were designed to work in Silos, in one way or another, I mean there were asylums as recent as 40 years ago, people with mental illness were locked up, not really part of society as such, to change that mindset to bring it in the mainstream is going to take some time, there is still stigma attached, people would not… they there is whether you want to work with people with mental illness, it is not the easiest population to work with , and second, uhm, you know having a problem in terms of discharging people back to the polyclinics and GPs because they don’t want to take our patients, and that, that is a big barrier as such. A lot of our patients would be better off, we see too many people who are stable who are within the clinic, they could have been discharged back to the GP or polyclinic, and the polyclinic could have definitely continued to follow up or the GP could follow up, but we can’t do that because they would not accept, even when they are stable for a long time, so those organizational barriers are there,

**References 3-4 - 1.39% Coverage**

what do you think has led to that reluctance to see people with SMI>?

PARTICIPANT: well it is more difficult and more time consuming, it is not easy to explain things and the likelihood that they will follow up in terms of what they are supposed to do is less., and of course there is the stigma and the mindset that this person with mental illness will not understand or be willing to sort of do the same things, although that is not true in most cases, that is the mindset people have.

**References 5-7 - 2.25% Coverage**

if we go back you mentioned that there was a worry that the person would not adhere to the TX, can you tell me more? So the GP would be reluctant to take somebody on if they feel that they would not comply?

PARTICIPANT: that is just one of the factors, the general feeling is that the person with SMI would not understand the need, and that is sort of the stigma, the mind frame people come up with when they see someone with SMI they think that the person is less likely to adhere comply and understand their condition. It is true that they may require more time, especially people with chronic schizophrenia, and intellectual disability, they mare need more time to explain the situation, so it is not an efficient use of their time, and perhaps their resources within the current setting.

**<Internals\\SP_140209_0110> - § 12 references coded [11.03% Coverage]**

**References 1-2 - 1.15% Coverage**

I know there are some people who tried, who we also referred to external parties for example certain hospitals with weight management programs and we referred some of our patients there, few , but several of them, and they faced some stigma there as well, you know “mentally ill patients” we don’t really want you in my gym , that sort of thing. Some of it was patient’s thoughts or… it is kind of poor self-esteem , so they did not want to go out there and do things where a lot of people were involved

**References 3-4 - 1.62% Coverage**

How did you learn about the experience of stigma, that might be deterring?

PARTICIPANT: some of the patients came and told us.

INTERVIEWER: ok

PARTICIPANT: you know “ they were looking at us in a strange manner”. Part of it could be the illness itself, that the illness hasn’t gone so they had ideas of reference, but I think most of it was true, quite genuine. There is a lot of body image issues as well, it is not only their mental illness which was the stigma, it was also body image issues, because it was many of them were already quite obese or big size to begin with, so they felt a bit out of place. And you know some of them did get quite negative comments. SO I think most of it was quite genuine.

**References 5-7 - 1.55% Coverage**

The other thing is the third thing is the stigma that they look at a mental illness themselves, the patient self-stigma, and the stigma they have experienced from other people. I am talking about non-patients, so when they go to an external fitness facility, they are looked upon with some, you know some disrespect, or whatever term you use to call it. So these factors get in the way of them doing things.

INTERVIEWER: and that would be the same for going to the polyclinic as well for the polyclinic, the schedule…

PARTICIPANT: uhm , yes going to the polyclinic I think the walking the timings yes, would be true, possible the stigma that is associated with a mental illness.

**References 8-9 - 2.53% Coverage**

INTERVIEWER: of course. And do you think the families who are willing to go through that sort of step differ from those that may be more reluctant because of stigma to involve themselves because of stigma with IMH?

PARTICIPANT: uhm I think it is more of ,… well if someone has come here to IMH and they are willing to take treatment for their family member , and they have decided, in EPIP it is a three year program, so if they are willing to kind of subscribe to the three year program then it is not about the stigma when the families start to disengage, it is more because of their caregiver stress, or they feel that you know things are not really changing, some disillusionment sets in, so we find that families disengage because of these reasons not because of the stigma. The stigma part of it comes in the initial stages when we try to get them into the program, we try to psycho educate them, so there are a few people who do not want us because it is from IMH. In those case we don’t even end up seeing the patient. We don’t end up following up with the patient because they decide “we don’t want IMH” .

**References 10-12 - 4.19% Coverage**

and those that decline services because of stigma, do they go elsewhere to receive psychiatric care?

PARTICIPANT: they may, depending on how bad their psychiatric condition is , I can remember , yes, because it happened yesterday. This young boy who came in , he had brief psychotic disorder, and the whole thing resolved within two weeks of which it took about a week’s medication and then he stopped it because , the family stopped it because he was well. Initially they said yes they would follow up with us, when they heard about EPIP but then they realized the label of IMH would be there so they made an appointment to go the Changi to see the psychiatrist, I don’t know if they will go because their appointment is at the end of the month , but here in this case it was more the IMH label , and you know “stigmatizing my son, and since he is already well he doesn’t really need a long follow up so it is ok if I take him somewhere else”. So we do have some patients who choose to go to other hospitals maybe other restructured hospitals, some private psychiatrists.

INTERVIEWER: ok

PARTICIPANT: and it is primarily because of the stigma, which is associated with IMH. At times they do come back, one as I mentioned, they can’t be handled at other hospitals, and the second is the cost. So they have several relapses they go to private hospitals and they find that by the time they have the third or fourth relapse the family can’t afford treatment, so then they come back. So in that case the feeling of being stigmatized is still there, the desire of not really coming back to IMH is still there, but their options are limited. So they do come back., it is more against their will that they come back.

INTERVIEWER: it is less a preference and…

PARTICIPANT: yes less of a preference and more because there is no other option left.

**<Internals\\SP_140214-0116> - § 3 references coded [7.86% Coverage]**

**References 1-3 - 7.86% Coverage**

INTERVIEWER: are there any other barriers?

PARTICIPANT: another one is stigma. That the uhm IMH that there is kind of a stigma for the patient. Most of the patients I think or family members prefer to bring their family members to see a GP. Even for mental health problem.

INTERVIEWER: so even if the condition is the same if the condition is psychiatric, having the condition is less important to stigma than the location you have it treated? Is that right?

PARTICIPANT: sorry, what?

INTERVIEWER: if people, family members and people with schizophrenia with psychiatric illnesses prefer to not come to IMH because IMH has stigma of metal hospital, does that mean the stigma surrounds the location and not the mental illness?

PARTICIPANT: … around the location… the stigma? Around the location, what exactly…

INTERVIEWER: I am not explaining myself properly; you mentioned that family members would prefer to see

PARTICIPANT: polyclinic

INTERVIEWER: elsewhere to avoid the stigma, but the person still has a psychiatric condition, but treated elsewhere? Right? So does that mean the stigma of coming from IMH is simply about the location? About coming to IMH or is there stigma around having a mental illness regardless of where it is treated?

PARTICIPANT: oh I see, I think both, both have the, that the, but the more with the location, you know, IMH has the reputation of the place for crazy people. So yeah. It is kind of the stigma that may prevent them from coming here for treatment.

INTERVIEWER: so they want to avoid it not so much because of embarrassment of having a mental illness, but because it is a place where people have notoriously been …

PARTICIPANT: this is just my imagination

**<Internals\\SP_151007-0097> - § 7 references coded [10.90% Coverage]**

**References 1-2 - 4.06% Coverage**

There are a lot of barriers if we talk about treatment for everything, barriers first of all considering that nobody wants to come to IMH generally speaking, however people who have been coming for many many years they have been used to coming and taking treatment from here. But because of the stigma, the discrimination, it is basically a mental psychiatric hospital, that is why, there are patients who still don’t prefer to come here, for any kind of treatment what so ever

INTERVIEWER: **so for these patients what do you usually do**

PARTICIPANT: of them there are case managers to track them, there are case managers to bring them back to the and of intervention the programs going on, CMHT support is there, community treatment support. Basically multi disciplinary uhm team of management is going on for those patients, but still, that’s in many cases that fails to bring them back to the treatment.

**Reference 3 - 2.86% Coverage**

So these are the situations where I can refer my patient to our medical in house colleague if they are working here. So that will definitely take care of a lot of things. And I think also help take the things forward to DE stigmatize this center.

INTERVIEWER: **how do you think it may DE stigmatize the center?**

**PARTICIPANT: DE stigmatize in the sense that it is not just a psych hospital, but also, if necessary my medical problem I can get from the same hospital.**

**INTERVIEWER: ok so that way it is not simply mental health , it is just a regular, but a hospital that has specialty in mental health rather than a mental health hospital**

**References 4-5 - 3.37% Coverage**

I don’t think there is really an issue of manpower, in fact the patient doctor ration or patient specialist ration I think in most of the general hospitals is better healthier in other hospitals than in IMH, that I am sure. But at the same time ironically the, they don’t see or like to treat people with severe mental illness, these kind of practice must change,

INTERVIEWER: yeah, and do you think it is down to stigma or understanding?

PARTICIPANT: no not exactly, maybe that, but more than that, and much more on paper is that anybody who needs to go with risky behavior , they are immediately sent to IMH. Because the IMH is the only government commission center where the patient can be formalized under mental health law for risky behavior

**References 6-7 - 0.61% Coverage**

PARTICIPANT: so these kind of things are difficult to change, this is the problem, nothing to do with stigma, it is a problem of policy.
