# Supplementary material for: The Impact of Stakeholder Preferences on Service User Adherence to Treatments for Schizophrenia and Metabolic Comorbidities
Source: PLoS One. 2016 Nov 16;11(11):e0166171. doi: 10.1371/journal.pone.0166171 (PMC5112999; doi:10.1371/journal.pone.0166171)
Supplement: S1 File — This file contains the nodes used to construct the themes reported in the manuscript. Including advice to others; expertise; insight into illness; instructions; looking after kin; preferences; relapse; resistance to doctor’s orders; social factors; social support; stigma; therapeutic alliance; and uneasy about initiating treatment. (ZIP) [file pone.0166171.s001.zip › Qualitative data/Therapeutic Alliance.docx]

**Name:** Therapeutic Alliance

<Internals\\HDL interview 2 20151111171452547 no audio> - § 1 reference coded [4.60% Coverage]

Reference 1 - 4.60% Coverage

Also pleased about the doctors at IMH who have been respectful and been able to produce rapport with him. They know how to handle him here. Prefers IMH for treatment of mental illness.

<Internals\\HDL study -Service user HDL_140211-0114> - § 3 references coded [4.73% Coverage]

Reference 1 - 0.80% Coverage

and they had your physicians, your psychiatrist, have they change your medication?

PARTICIPANT: no, in terms of quantity yes.

INTERVIEWER: yes.

PARTICIPANT: in terms of duration yes, the types specifically no.

INTERVIEWER: ok

PARTICIPANT: I think they actually had kind of targeted at my condition exactly. And I guess this proved to be effective. So, I just go along with it.

Reference 2 - 2.27% Coverage

and what was the cause of the 2 relapses? Was it because you sort of have problems with medications or?

PARTICIPANT: not.. not… maybe partly because I was busy working. So not too cautious of taking it, you know. Even though I went for my appointments so forth. But also is actually the same cause of how I end up in first place actually developed again over the years, you know. So, that’s why Dr actually try to alert me, you know. Can you recognise when is the symptoms will.. when the symptoms will cause, you know. You… In the first place you were here because of stress, so over the years actually is.. stress also 1 factor, so on my 2nd and 3rd relapsed came about, it was still cause of stress. So he had asked me to actually kind of identify. If stress is one of the factor, try to reduce. Say you know to try and prevent. So stress and some other issues. Like relationships and then monetary problems, you know. And so over the years it has improved and I kind of recognise this is actually the cause, the symptoms, which I try to minimise to prevent the relapse from happening.

Reference 3 - 1.66% Coverage

PARTICIPANT: ahh.. because sometimes that. It can be very fast. The waiting period can be longer than the actual consultation you know. Sometimes its like you in, you out already. You know. So erm.. its like. Dr ask you, are you ok? yes. Ok, raise you hands, see the trembling. How’s your more days, did you sleep well? Did you eat well? Ok everything fine you know. So they ask some questions. some depends. These are the general questions. some very fast, 2-3 questions and out you go. But some is ah.. take a minute, look through your records. Ok, you reducing weight, ok. then from there through the... I man more sharing, so I ask can I reduce for the.. oh, reduce as it is, you know. They not comfortable, then some share the statistics. The research study. So.. it depends on the Dr also.

<Internals\\HDL Study_service user HDL_151210-0137> - § 1 reference coded [2.02% Coverage]

Reference 1 - 2.02% Coverage

ok. have you have any major problems since you started taking medication or started your treatments?

PARTICIPANT: some problems I encountered. Dizziness, drooling, ?? (18:07 )here and there. The whole.. the whole body is very lethargic. So I .. I push the Dr to.. 3 years back. the Dr quite friendly to me. Is a Japanese DR. yah. Then he slowly, he change my medication, then I get treated. He then.. they refer me back to CWC.

<Internals\\HDL Study_service user HDL_151218-0134> - § 1 reference coded [1.98% Coverage]

Reference 1 - 1.98% Coverage

INTERVIEWER: but now if your GP can follow you. Would you prefer to see the GP? And only go to the polyclinic and not bother coming to IMH. Or is it

PARTICIPANT: GP… I think the... don’t know. because I’ve been seeing the psychiatrist here for 13 over years. So, I’m quite used to the... yah. The environment

INTERVIEWER: and its always the same psychiatrist?

PARTICIPANT: yah, Dr Johnny.

INTERVIEWER: so it must be a good relationship then. to have with a

PARTICIPANT: not sure. I think is ah... comfortable la. Yah, I have been seeing him for 13 years.

<Internals\\HDL Study-Service User HDL_151203_0061> - § 1 reference coded [1.51% Coverage]

Reference 1 - 1.51% Coverage

Doctors change

Interviewer: How often?

Participant: I don’t know the doctord not the same everytime, keep changing…the doctors keep changing, it’s not the same doctor every two weeks, every 4 weeks, every 4 weeks the doctor keep changing and I don’t know which doctor, I can’t remember the doctor also

<Internals\\HDL study-service user HDL_151209-0152> - § 3 references coded [2.63% Coverage]

Reference 1 - 1.23% Coverage

They change the medication in the ward. So, don’t know why. Everything the Dr on the ward control my decision want to leave the ward or not. very hard for me to take the decision I want to leave. Later I go to the ward, I want to go out, but they lock me up. It’s totally.. in the …the hands, the hands is totally in the Dr decision. If the Dr decision not really happy… (Laughs). If the Dr not feeling happy today, they will not give you to see him. Then say ok, he want me I feel not very happy. Second I will not discharge you.

Reference 2 - 0.61% Coverage

INTERVIEWER: why? Because remember you said that MOs sometimes not very professional. Yah? At IMH

PARTICIPANT: the MO never made any decision because ah.. he can’t make the decision by.., he say next time he want to talk to me, this one no need to talk to me.

Reference 3 - 0.79% Coverage

PARTICIPANT: he keep on talking to me. Tell me I don’t know him like that. See Dr or not, then he come at me and say. Aiyah, so long. He say. You know why so long? They see Dr Yeo Sui lin. Cause Dr Yeo Sui lin like to listen to other people stories.

INTERVIEWER: so he spends more time with you? likes to listen to other people stories

<Internals\\HDL Study-Service User_140113-0128> - § 2 references coded [6.89% Coverage]

Reference 1 - 4.36% Coverage

INTERVIEWER: is that only because he’s an expert or is there anything else? So what about the support that he gives you is important?

PARTICIPANT: actually he is the first psychiatrist I came across that actually advise me on developing my own personal medication. As in medicine. Personal medicine. And he is the first psychiatrist that I came across as he doesn’t want me to be too reliant on the medication itself. And he has actually introduced peer support to me. All these changes is the first that I have ever encounter. Cause back in Mt E, the Dr only give me the medication and then he doesn’t really care about how I was coping. He just. Every time I see him I have to wait for about an hour to see him and then I was inside his room for about less than 5 mins and I was out. So he was basically like seeing whether I was well and then he go on to prescribe the medication and he wasn’t talking to me. So that kind of treatment is totally different with what I experience with Dr Leong.

INTERVIEWER: so he’s.. here you have gotten more connection with the Dr. He understand you better.

PARTICIPANT: yes.

INTERVIEWER: and he’s taking the time to sort of follow up with what’s going on.

PARTICIPANT: yah.

Reference 2 - 2.53% Coverage

INTERVIEWER: ok. what about what you like the most about the care you getting now?

PARTICIPANT: there is a listening ear and then the support as well as..The thing I like about.. not sure about the other doctors but.. the thing I like about Dr Leong is that he actually empower us and… he once said that.. yah, he was joking with me. He once said that he give me the tools. The tools to the illness management self-management and recovery. He actually pushes us to.. pushes me to actually think on my own and.. he sort of pushes me forward. Yah, and doesn’t want me to rely too much on the medication. And he changed my mindset to become a positive one. He doesn’t… doesn’t feel uncomfortable with him. Yah

<Internals\\HDL Study-Service User_140209-0109> - § 1 reference coded [6.57% Coverage]

Reference 1 - 6.57% Coverage

PARTICIPANT: I think the other thing is cost. Another thing is maybe looking for the doctor can listen to what I say. Yes, that’s the other one important.

INTERVIEWER: can you tell me more about that?

PARTICIPANT: to see my problem la. Telling my problem may works in other place. The things I encounter during my works la. And all that la.

INTERVIEWER: so you like bring able to speak to somebody else about the difficulties you have at work?

PARTICIPANT: ya ya.

INTERVIEWER: and finding somebody who is able to listen is difficult.

PARTICIPANT: ya.

INTERVIEWER: does your psychiatrist here listen to that?

PARTICIPANT: I guess.

INTERVIEWER: ya?

PARTICIPANT: ya.

INTERVIEWER: do you see one or is it always different?

PARTICIPANT: different.

INTERVIEWER: so why is that important to you?

PARTICIPANT: to gives.. to seek advice la. Whether is important or not important. Ya.

INTERVIEWER: and so being able to speak to somebody helps to make those choices?

PARTICIPANT: ya

INTERVIEWER: ya.

PARTICIPANT: opinion and all that la.

INTERVIEWER: and does.. can you do that with your GP?

PARTICIPANT: ah, yes.

INTERVIEWER: so you can also ask advice from your GP

PARTICIPANT: ya

INTERVIEWER: ya.

PARTICIPANT: but GP I think.. because their job is not psychiatrist. They won’t be able to listen to you for.. every time la. A long … after some.. you got some other patients and all that. He’s not specialist in this area. His job is not specialist in this area. So, he may not very time la. Ya

<Internals\\SP 140130-0095> - § 2 references coded [3.17% Coverage]

Reference 1 - 2.09% Coverage

we are really not the best at diabetes, hypertension and …er hypercholesterolemia are not simple conditions you have an entire specialty devoted to that sort of thing endocrinology...and even gps do it my wife is a gp she knows what…her field, their knowledge of these 3 conditions, she does so much of it in the polyclinics it’s really different from me my questions are what medicine should I give them? And she’s asking what is a metabolic risk factor? What’s your family history like? What’s your diet like? what is your weight profile? Er… I don’t know, what medicine do I give them? So I like when people speak psychiatrist, What’s the best medicine for depression? What type of depression so it’s different when I speak to my wife about this and she always sighs when I talk to her about what is the best thing to do…what I do…because I don’t ask the right questions, I don’t have the right information same situation when we see patients with mental disorders being treated by gps sometimes they do well, those that don’t often it’s an issue of lack of knowledge and training that’s all. They can give exactly the same medication that we can sometime their therapeutic relationship is better than we have, but it’s often an issue of knowledge and training and to…

Reference 2 - 1.08% Coverage

It’s selection bias the ones that i see and I do have patients who I treat their metabolic conditions I repeat their metformin, their diabetic, hypertensive medicine there are a small subset of patients that do this they want me to give it to them they don’t want the inconvenience of going anywhere else, they feel fine taking it this way, they want to treat their metabolic conditions and they don’t really see the point of seeing another doctor in the polyclinic whom they don’t know, there’ s no personal relationship and they get in their mind exactly the same care which just equates to medicine so the patients whom I am doing it for they like it.

<Internals\\SP 140131-0096> - § 1 reference coded [3.93% Coverage]

Reference 1 - 3.93% Coverage

therapeutic alliance?

INTERVIEWER: yeah

PARTICIPANT: its’ a bit hard, you mean maintain rapport with the patient while they are trying to build a therapeutic relationship, so I is quite hard to take on a more paternalistic role as in telling them what to do, we try as far as possible to agree on certain things trying to explain to them why they need the medication, we don’t want to force it on them because we know once they go home, we can’t control it once they are home. They can easily stop medication … so uhm we don’t try and force the medication on them so, it is more reaching an agreement as to what kind of medication they want with the minimal side effect , so often we do explain to them that with the medication they can put on weight, it does affect their glucose and lipids eventually, so having that conversation early on does help in our choice of antipsychotics, so we may choose one that may probably be less likely to induce this, these metabolic syndromes, so that is how we try and circumvent it

<Internals\\SP_140109-0130> - § 3 references coded [8.07% Coverage]

Reference 1 - 3.03% Coverage

the fact that you have to stabilize them first. what do you mean by that.?

PARTICIPANT: the problem is that most when they come to me and if I have to say immediately that I am going to refer you back to a GP, usually it would not work , they say “no I don’t want” and that is exactly the reason why they have not been seeking treatment for years, yeah um, and and and there that comes my role where I treat them convince them they have this situation which has to be treated, and showing that treatment does help and improve on some of the parameters for example their blood glucose and all that. And when I have a better rapport that is when they will believe me and they are willing to take the referral to go to a polyclinic.

Reference 2 - 2.94% Coverage

and they have no trouble sort of accepting that? What is curious to me is why they would disengage with the polyclinic in the first place and then why would they, how do you get them to listen to you?

PARTICIPANT: I suppose the difference is being a psychiatrist, trained me in the sense of building rapport, and knowing what the patients wants and addressing their concerns, and actually it helps a lot, compared to if I am allowed to compare to a polyclinic doctor where uhm , they do not have a lot of these experiences in communications or even in the even limited by their time as well, all these factors come in as well, yeah, so I believe that is the reason I can usually engage them with treatment.

Reference 3 - 2.10% Coverage

so you don’t think that the polyclinic physicians have the same type of ability to produce a rapport?

PARTICIPANT: let me put it this way. I would not say they don’t have but they probably been trained in the field of mental health I am more of this, this is especially true for people with mental illness, so you probably have to, the way we manage them, or the way we communicate with them has to be a bit different, and the polyclinic doctors may not always have the full experience in that sense. yeah

<Internals\\SP_140117-0080> - § 4 references coded [3.50% Coverage]

Reference 1 - 0.81% Coverage

so then after they see the doctor then it depends on really seeing the doctor, whether you like the doctor, whether the relationship with doctor…right if you think the doctor just wants to get you out of the room, a lot of times you can’t share what is actually bothering you and er diabetes, hypertension, and high cholesterol may not be something that is bothering them at all because all these conditions are silent they don’t give you problem, there’s no pain, there’s no discomfort from diabetes

Reference 2 - 0.74% Coverage

if the person the service user the patient coming here is difficult for them the most convenient is the gp downstairs shouldn’t the gp be the one that you know looks after most of the things? Why go to the polyclinic right? Rather than come here and (14:00-14:01) psychiatrist but if the patient say yes I will like to work with my psychiatrist just because i trust my psychiatrist we have a good relationship and the person is engaged yah then I think we…

Reference 3 - 0.97% Coverage

I think er individuals have different preferences some is convenience in terms of the location, the time, the effort, you know how to get all the treatment done right? So it’s the convenience some it’s actually because they have the relationship they only trust you so they will come because they only trust you and trust what you say they don’ trust anybody else so I think there is a whole spectrum so one is actually the convenience the accessibility but the other part is actually the rapport or the relationship you know and how we actually do it whether we do it efficiently and effectively

Reference 4 - 0.98% Coverage

usually when we don’t feel comfortable to treat we always refer out so I think today I actually made a statement we shouldn’t be keep referring people out because I can become a postman what? keep posting people out then where’s the treatment and where’s the relationship, there’s no relationship but if you’re the trusted advisor, you’re the trusted person basically it is to liaise rather than to refer yah right? so I tend not to use the word referral I tend to use request (19:30) request for this person right so who can i link this person with so that this person can get the most appropriate care?

<Internals\\SP_140120-0083> - § 2 references coded [2.08% Coverage]

Reference 1 - 0.59% Coverage

I think gp now their business fairly competitive they need to see patients fast you know and with high volume to make their business viable whereas sometimes our patients need time to talk yah to maintain the trust

Reference 2 - 1.49% Coverage

the patient I think the gp can be someone I think er to co-ordinate the care and can be someone who that the patient feels er you know er that he trusts him yah and the gp can actually can also probably some of our gps er you know previously whom I’ve spoken to they know the family members the extended family members er some of them have watched the patients grow up from young you know the more elderly gp…and so there’s actually a lot of trust and rapport there so I think you know that’s really important for compliance and ..yah follow-up yah

<Internals\\SP_140123-0081> - § 1 reference coded [2.02% Coverage]

Reference 1 - 2.02% Coverage

I think personally it all depends again on whether you’re running what we call a medical clinic I mean a medical officer clinic (26:50) or personal clinic doctor. For some of the specialist I know my patient well enough, I build rapport with my patient so i think you are, you may be able to convince the patient much strongly in that sense but if you’re only seeing the patient like a medical officer just see once or twice then the patient may not really have a good rapport with you and may not accept your recommendation so you ask me whether this hospital is moving forward, I think we are moving towards what we call (27:29)…and also the the er same doctor seeing the same patient or at least the same team is seeing the same patient I think that may be probably be good…er but knowing the patient well enough you may not have to spend half an hour or 20 minutes it may even be a limited period of time 10-15 minutes(27:44-27:45), you’ll still be able to cover a fair bit of the updates of the patient I think they

<Internals\\SP_140125-0086> - § 2 references coded [3.42% Coverage]

Reference 1 - 1.93% Coverage

if patients have been seeing us for a long time perhaps they will assume that doctors will listen to their trouble, if they bring up their trouble to the GPs for example, if they are dismissed, it doesn’t form that rapport I think the same problem I will say that the flip side is also true a lot of my GP friends and seniors I have met they are extremely good they probably do more counseling than psychiatrists so they get the treatment when they come to see us and they bring up a concern they have physically and we dismissed it ourselves so I think we stigmatize sometimes as well patient complain about headache, giddiness we will assume that it is due to a panic attack, it is due to a psychiatric illness, and we will say no it’s nothing serious and it turn out to be a physical problem and then this will cause them to lose confidence in us too as well

Reference 2 - 1.49% Coverage

you see even for example my mum stays with me now, she still goes back to her own doctor many…er quite far away I mean in Singapore of course there is nowhere that’s really far, she doesn’t see the doctor around my area, she goes back to her own doctor

Interviewer: So if she has that rapport, that bond, that sort of…

Participant: Yah and I find that whatever the doctor say is probably more powerful than what I as a doctor tell my mum, and I’m fine with that and I know that the doctor is pretty reasonable so it’s not a problem as well, so she manages everything and I know she talks to my mum about a variety of things my mum goes for all the screening that

<Internals\\SP_140126-0088> - § 1 reference coded [1.20% Coverage]

Reference 1 - 1.20% Coverage

For those who don’t mind , generally they already have a relationship with the polyclinic for example i have already been seeing this polyclinic for my diabetes, and i am here to see you because this is a specialist in psychiatric care which i need to see, so they may have prior relationships with IMH and polyclinics.

<Internals\\SP_140202-0098> - § 3 references coded [4.46% Coverage]

Reference 1 - 1.27% Coverage

no matter how many years of treating their psychiatric illness they may not develop the insight that they have an illness but then why do they come to see you is because they have kind of formed a friendship, a rapport with you so they believe that you are doing something so in that sense the belief that has taken place over all these years would help them to see er you and perhaps to enable you to give them treatment for other conditions something which a polyclinic doctor who is very busy may not have the ease of time

Reference 2 - 0.39% Coverage

it’s someone you have grown up with…you’ve seen, communication maybe fairly straight forward relatives can probably accompany them more easily since it’s nearby

Reference 3 - 2.81% Coverage

unfortunately patients are seen in what we call common pool so a common pool meaning if their conditions are so well managed it’s actually being treated by non-specialist as these are medical officers in training so it may be a different doctor each time it may be a fairly new experience with each consult because you are talking to somebody you’ve… I guess from a patient’s perspective it may come across as I’m going through my condition with somebody totally new and I may feel that it is this doctor really aware of what I have been through or is it just er..just because my condition is so stable therefore there is no need to know what my condition is like so I guess in the sense if your condition is really very stable then probably it’s gonna be better for the for the er perspective of seeing a family physician who knows you and is probably able to give you more dedicated time and treatment that you need as opposed to coming back to a restructured hospital and seeing a general pool clinic where every time they see a different doctor so this is one of the barriers to care I can foresee in a patient’s perspective when they come back to a hospital,

<Internals\\SP_140203-0100> - § 1 reference coded [0.75% Coverage]

Reference 1 - 0.75% Coverage

They have a level of rapport here, and also that we know how to deal with people with mental illness, so the communication skills over the years they improve, so that is one of the big factors why people prefer to have their physical health also managed within IMH.

<Internals\\SP_140209_0110> - § 1 reference coded [0.56% Coverage]

Reference 1 - 0.56% Coverage

it could be the rapport they already have with our with the treating team, so instead of going and disclosing everything to someone else, and then thereby repeating the whole story, they already know us , so they hope we can treat them as well.

<Internals\\SP_140215-0120> - § 2 references coded [3.84% Coverage]

Reference 1 - 0.82% Coverage

Well I mean that is usually the context of the…established kind of therapeutic relationship. So I think there’s already enough trusts and (16:03) nearly to the point you can actually tell them not so good news, and that they need to kind of do certain things. So I find that’s importance.

Reference 2 - 3.01% Coverage

I actually don’t think so…although I think in those instances…you had to set up, or you have to establish a rapport and that of course I think takes time. So in the context where it’s kind of a…not as regular kind of clinics where you have not seen that you are a regular patient, I think there is still some room. I think as long as we kind of…we talk about the same things, the really impressing upon them that this is important for them, that they need to be basically on the lookout – their risks, benefits, and we kind of need to really monitor early and prevent further (?) (18:40) or complications. I think once that’s laid out in the context of trusting…relationship, even in the short session I think once the patient sees that you’re genuinely concerned about the how this is important for them, then I think they will be able to understand. So I don’t think it necessarily just in the sole province of the longer term regular kind of a consulting relationship. It can be done in a…even in a one-off session, telling them why this is important.

<Internals\\SP_151210-0132> - § 1 reference coded [3.49% Coverage]

Reference 1 - 3.49% Coverage

I think those that prefer to be seen in the outside of IMH usually I think have , they may be working so they prefer to see GPs after office hours, they may have fairly mild psychiatric disorders or very stable ones that they have, they have not developed a strong relationship with us, for example they , at some point come quite regularly for stabilization of their mental disorder , but maybe now they come once every 6 months to get a refill, and they haven’t had any major psychiatric issue, therefore, and when they come they see different doctors in our clinic, so once the relationship becomes less strong it is not person-based, and they have the same time developed a better relationship with the GP because they may be seeing the GP once every month or two, for cholesterol or problems with hypertension, then they would prefer… I think really the difference in the patient is the patient’s relationship with the primary care provider. If it is a good relationship with the external provider I think they would want to go see that provider more,
