# Supplementary material for: The Impact of Stakeholder Preferences on Service User Adherence to Treatments for Schizophrenia and Metabolic Comorbidities
Source: PLoS One. 2016 Nov 16;11(11):e0166171. doi: 10.1371/journal.pone.0166171 (PMC5112999; doi:10.1371/journal.pone.0166171)
Supplement: S1 File — This file contains the nodes used to construct the themes reported in the manuscript. Including advice to others; expertise; insight into illness; instructions; looking after kin; preferences; relapse; resistance to doctor’s orders; social factors; social support; stigma; therapeutic alliance; and uneasy about initiating treatment. (ZIP) [file pone.0166171.s001.zip › Qualitative data/Uneasy about initiating treatment.docx]

**Name:** uneasy about initiating treatment

**<Internals\\SP_140116-0079> - § 2 references coded [3.46% Coverage]**

**Reference 1 - 2.14% Coverage**

**Because most of our patients in first episode they don’t present with diabetes or they don’t develop very severe hyperlipidemia so you can see the levels creeping up I think why I’m telling s in the past when I have referred patients they have come back they have said “oh the polyclinic told me that the blood level is not that abnormal, I just need to do exercise so I’ll be ok .” so I’m not sure at what time when is the time to rightly refer cases or when I can just advise them about diet, exercise and continue to monitor**

**Interviewer: Hmm. So do you think that sort of uncertainty explains why there was a need for Alvin to produce an algorithm in the first place?**

**PARTICIPANT: Yes yes definitely. Yah**

**Reference 2 - 1.32% Coverage**

**Okay..are you the expert in this area so you you’ll be looking at the GPs to provide with the expertise to understand the metabolic syndrome …what needs to be done**

**PARTICIPANT:Yes**

**Interviewer: Ok**

**PARTICIPANT: Yeah. I think so. I don’t feel comfortable if I pick up and start treatment although I have done it a few times. But for the lipids I have done it but for glucose I don’t feel comfortable starting them on any hypoglycemic agents**

**<Internals\\SP_140120-0082> - § 1 reference coded [1.10% Coverage]**

**Reference 1 - 1.10% Coverage**

I think if there is to a certain extent, an element of risk there. I mean if you’re not familiar with the condition, you don’t even know how it’s been diagnosed, you’re not familiar with the latest drugs, then chances are you just leave it. You’re not doing anything…very safe, then doing something, end up patient getting, y’know, not feeling very well (08:42) and ending in complication.

**<Internals\\SP_140123-0084> - § 1 reference coded [0.90% Coverage]**

**Reference 1 - 0.90% Coverage**

I guess the challenge is that I don’t feel to a certain extent to start medication for diabetes, or high cholesterol, hypertension the basic level I’m comfortable but when it, they are not responding or they need maybe multiple medication er to manage that illness erm then I start I don’t I’m not that confident so would have liked the support of a gp or a specialistwho we could work closely with then

**<Internals\\SP_140125-0087> - § 2 references coded [1.62% Coverage]**

**Reference 1 - 1.23% Coverage**

I think generally there is an apprehension about treating medical issues, medical conditions, medical comorbidities, because I think the more senior you are the further you are away from medical school and sometimes the skills and knowledge is not updated. In that medical aspect., so maybe the confidence to manage is a bit less

**Reference 2 - 0.39% Coverage**

the other reason could be just a fear of you know not knowing what to do with an abnormal blood result.

**<Internals\\SP_140203-0100> - § 1 reference coded [1.18% Coverage]**

**Reference 1 - 1.18% Coverage**

we are working within our GP partnership scheme, to train the GP to manage psychiatric conditions, and they are quite happy to see some of our patients and manage both the physical and mental health part , it is possible , but they have the same, you know the same comfort level, it is easier for them to sort of see someone with the flu or prescribe hypertensive, rather than deal with the mental health part as well.
